# Ligand Design Criteria for the Stability of High Oxidation State Praseodymium Complexes

**DOI:** 10.1021/acs.inorgchem.5c05024

**Published:** 2026-01-13

**Authors:** Tyler-Rayne Nero, Chad M. Studvick, Andrew C. Boggiano, Maximilian G. Bernbeck, Ivan A. Popov, Henry S. La Pierre

**Affiliations:** † School of Chemistry and Biochemistry, 1372 Georgia Institute of Technology, Atlanta, Georgia 30332-0400, United States; ‡ Department of Chemistry, 1076 The University of Akron, Akron, Ohio 44325-3601, United States; § Department of Chemistry, 6760 Washington State University, Pullman, Washington 99164-4630, United States; ∥ Nuclear and Radiological Engineering and Medical Physics Program in the School of Mechanical Engineering, Georgia Institute of Technology, Atlanta, Georgia 30332-0400, United States

## Abstract

The isolation of high-oxidation-state lanthanide complexes requires a balance of electron-donating ligand environment, steric protection, and ligand redox stability. Herein, we report the synthesis of a new imidophosphorane ligand, **NPC** ^**2**^ ([NP­(^*t*^Bu)_2_(pyrr)]^−^; pyrr = pyrrolidinyl), and its ability to support homoleptic Ce^3+^ and Pr^3+^ complexes that afford access to Ce^4+^ and electrochemically observable Pr^4+^ and Pr^5+^. The structures, electrochemistry, and computational analyses of tetrahomoleptic **NPC** ^**2**^ complexes of Ce^3+^, Ce^4+^, and Pr^3+^ are compared with previously reported analogues supported by **NP** ^*^ ([NP­(1,2-bis-^*t*^Bu-diamidoethane)­(NEt_2_)]^−^), **NPC** ^**1**^ ([NP­(^*t*^Bu)­(pyrr)_2_]^−^), and **NPC** ^**3**^ ([NP^*t*^Bu_3_]^−^; ^*t*^Bu = *tert*-butyl) ligands. Across the **NPC** ^***x***^ (*x* = 1–3) series, ligand substitution results in modest changes in redox potentials, consistent with minimal perturbation of the *f*-orbital manifold. Despite similar electronic donor properties, **NPC** ^**3**^ provides increased stabilization of Pr^4+^ and Pr^5+^ complexes due to enhanced steric protection, leading to improved electrochemical reversibility and chemical stability relative to complexes of **NPC** ^**1**^ and **NPC** ^**2**^. Systematic density functional theory calculations on both experimentally isolated and nonisolated complexes rationalize the experimentally observed insensitivity of the Pr^4+/3+^ and Pr^5+/4+^ redox couples to ligand substitution across the **NPC** ^***x***^ series, while identifying steric encumbrance, electron-donating ability, and counterion-retention as key factors governing the accessibility and stabilization of high-oxidation-state lanthanide complexes.

## Graphical abstract

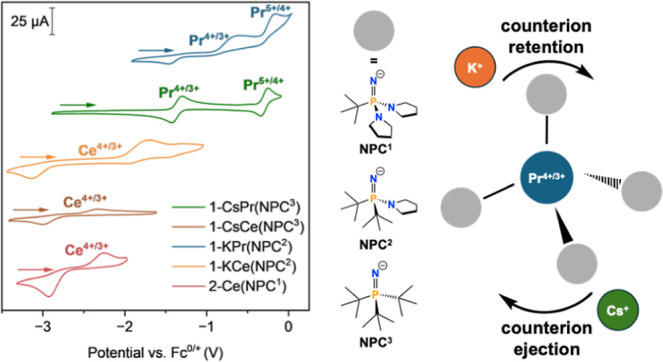

## Introduction

Understanding the ligand features that govern the stability of high-oxidation-state lanthanide complexes is essential for accessing unusual valence electronic structures in the early lanthanides. The recent isolation of a molecular Pr^5+^ complex^1^ represents a rare case where molecular lanthanide redox behavior mirrors that of group 5 transition metals (V, Nb, and Ta) and its actinide congener (Pa). Stabilizing the highly oxidizing Pr^5+^ ion enables access to redox trends predicted by the periodic table. Prior to this, Pr^5+^ had only been detected as a transient species in the gas phase or under matrix-isolation conditions in solid noble gases, rather than as isolable extended-solid compounds or discrete molecular complexes.^2–5^ However, with only one reported example, it is critical to parse the ligand characteristics that facilitated its characterization. As discussed in a recent theoretical review, the limited accessibility of high-oxidation-states within the 4*f* series arises from compact and deeply bound 4*f* orbitals, yielding the attainable oxidation state maximum within the lanthanides at Pr^5+^.^6^ Although high oxidation states are accessible in early lanthanides (Ce–Pr), Tb^4+^ is also well-known in solid state oxides and fluorides, and has recently been realized in well-defined molecular complexes.^7–13^

In condensed phases, the lanthanides primarily occur in the 3+ oxidation state.^13^ Accessing higher oxidation states can afford novel reactivity,^12,14^ coordination chemistry,^8,9,11,12,15–17^ separations,^11,13^ and unique electronic structures that may find applications in quantum and magnetocaloric material design, as well as component ions for applications in quantum information science.^18–21^ Recent advances in the La Pierre and Mazzanti groups have identified two classes of ligands capable of stabilizing molecular lanthanide complexes in the 4+ oxidation state: the imidophosphorane and siloxide ligand frameworks.^7,8,10–12,15–17,22–24^ Despite these developments, isolating molecular complexes of 4+ and 5+ lanthanide ions remains a significant challenge. Expanding this class of complexes requires understanding the physical basis of their observed stability to guide the development of novel ligand frameworks to modulate electronic features and chemical reactivity.

Among the ligand classes established so far, imidophosphorane scaffolds have proven particularly effective in stabilizing high-oxidation-state lanthanide complexes and significantly shifting their redox potentials to more cathodic potentials, owing to their tunable electronic and steric properties.^1,7,13–15,22,25–28^ Imidophosphorane ligands are monoanionic 1σ/2π donors with zwitterionic character. Substituent variation at phosphorus provides access to steric control, facilitating stabilization of high-oxidation-state lanthanides and actinides. The ligand **NP** ^*^ ([NP­(1,2-bis-^*t*^Bu-diamidoethane)­(NEt_2_)]^−^), was designed for its steric protection and electronic effects that enhance σ/π basicity;^7^ **NPC** ^**1**^ ([NP­(^*t*^Bu)­(pyrr)_2_]^−^) for its ability to lower symmetry and reduce structural disorder;^29^ and **NPC** ^**3**^ ([NP^*t*^Bu_3_]^−^; ^*t*^Bu = *tert*-butyl) for its steric hindrance and potentially increased electron donation with respect to **NPC** ^**1**^.^1,14,15,30^ The effects of ligand donors have been noted by the electrochemical differences in redox potentials, with **NPC** ^**3**^ achieving the most negatively reported Ce^4+/3+^ *E*_pc_ potential (−3.01 V vs. Fc^0/+^), and facilitating the isolation of Pr^4+^ and Pr^5+^ complexes, and underscoring the exceptional stability conferred by this ligand framework.^1,14,15,30^

Previous studies have demonstrated that the redox potentials of coordination complexes can be tuned through steric modification and incorporation of intercalated counterions.^27,31–38^ In particular, alkali metal identity influences redox behavior across multiple ligand platforms.^36–39^ It has been shown in our earlier work that the *E*_pa_ potentials of tetrahomoleptic Ce^4+/3+^ complexes supported by the **NP** ^*^ ligand shift the *E*_pa_ by ∼600 mV, depending on the identity of the alkali metal counterion incorporated into the ligand framework.^27^ The thermodynamic drive for counterion ejection upon Ce^4+/3+^ oxidation increases with counterion size, from Li^+^, which resides closer to the Ce center, to Cs^+^, located at the periphery of the secondary coordination sphere. In this prior work, computational modeling of electrochemical data showed that oxidized structures retaining the counterion more accurately reproduced the experimental *E*_pa_ values for complexes with smaller cations (Li^+^, Na^+^, K^+^) compared to those with larger cations (Rb^+^, Cs^+^, K^+^([2.2.2]-cryptand), and K^+^(18-crown-6)_2_).^27^ This trend reflects the greater structural reorganization required upon oxidation when smaller counterions are present. These findings indicate that both the counterion retention and ligand steric profile are important considerations in determining the oxidation potential of the anionic Ln^3+^ complexes.

In this work, we investigate how variations in ligand architecture influence the stability of high-oxidation-state praseodymium complexes, with direct comparison to their cerium analogs (Figure 1). To extend our understanding of the donor characteristics of the imidophosphorane ligand, we synthesized a new series of tetrahomoleptic compounds introducing the [NP^*t*^Bu_2_pyrr]^−^ ligand (**NPC** ^**2**^) including, [KPr^3+^(NPC^2^)_4_] (**1-KPr­(NPC** ^**2**^ **)**), [KCe^3+^(NPC^2^)_4_] (**1-KCe­(NPC** ^**2**^ **)**), and [Ce^4+^(NPC^2^)_4_] (**2-Ce­(NPC** ^**2**^ **)**) (Figure 2B–D). These complexes were compared to previously reported tetrahomoleptic Ce and Pr complexes supported by the **NPC** ^**1**^ and **NPC** ^**3**^ ligands: [K­(2.2.2.-cryptand)]­[Ce^3+^(NPC^1^)_4_] (K222 = K­(2.2.2.-cryptand) (**1-K222Ce­(NPC** ^**1**^ **)**), [Ce^4+^(NPC^1^)_4_] (**2-Ce­(NPC** ^**1**^ **)**), [CsPr^3+^(NPC^3^)_4_] (**1-CsPr­(NPC** ^**3**^ **)**), [Pr^4+^(NPC^3^)_4_] (**2-Pr­(NPC** ^**3**^ **)**), [Pr^5+^(NP^*t*^Bu_3_)_4_]­[X^–^] (X^–^ = tetrakis­(pentafluorophenyl)­borate or hexafluorophosphate) (**3-Pr­(NPC** ^**3**^ **)**), [CsCe^3+^(NPC^3^)_4_] (**1-CsCe­(NPC** ^**3**^ **)**), and [Ce^4+^(NPC^3^)_3_] (**2-Ce­(NPC** ^**3**^ **)**).

**Figure 1:**
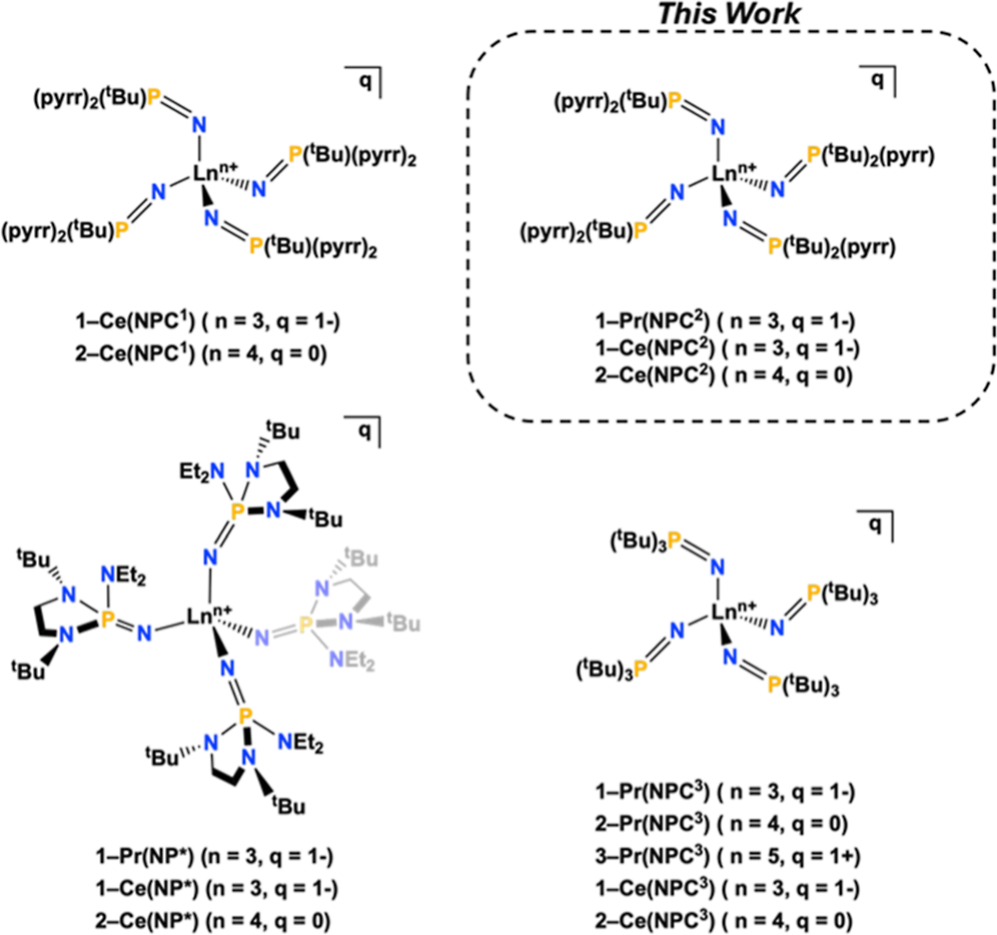
Homoleptic imidophosphorane complexes of Pr and Ce, highlighting variations in ligand architecture.^1,15,22,25,26^

**Figure 2:**
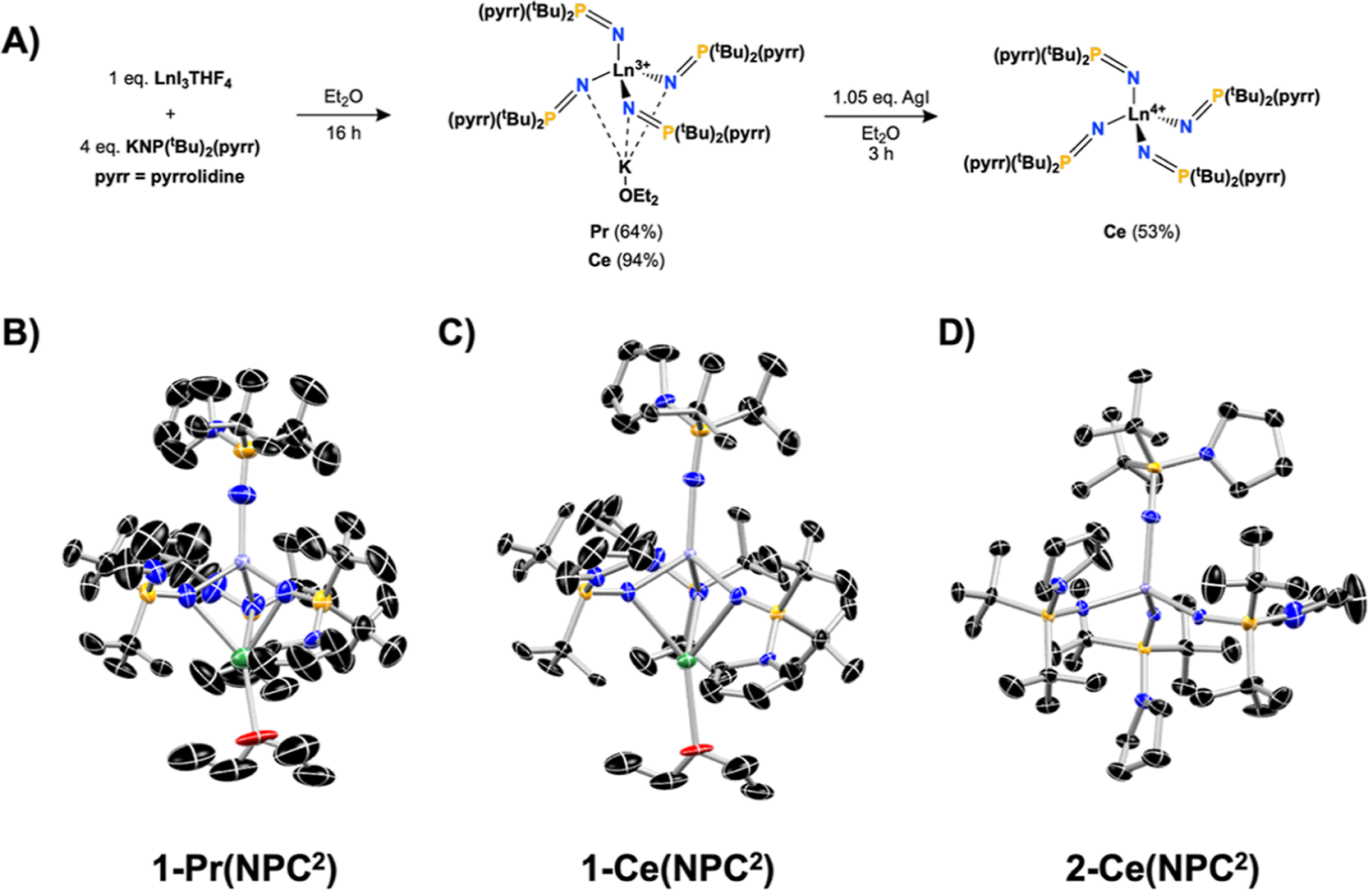
(A) Synthetic route to **1-Pr­(NPC** ^**2**^ **)**, **1-Ce­(NPC** ^**2**^ **)**, and **2-Ce­(NPC** ^**2**^ **)**. Truncated molecular structures determined by SC-XRD of (B) **1-Ce­(NPC** ^**2**^ **)**, (C) **1-Pr­(NPC** ^**2**^ **)**, and (D) **2-Ce­(NPC** ^**2**^ **)** with thermal ellipsoids (C = black, N = blue, P = orange, K = green, O = red, Ce/Pr = purple) shown at 50% probability. All hydrogens have been omitted for clarity.

Due to synthetic considerations, not all ligand–counterion combinations within the **NPC** ^***x***^ (*x* = 1, 2, 3) series could be isolated in all oxidation states. However, computational modeling enabled a complete survey across all ligand variants and oxidation states. Accordingly, density functional theory (DFT) calculations were performed on both isolated and nonisolated species across the Ce^4+/3+^ and Pr^5+/4+/3+^ systems to systematically evaluate the donor properties of **NPC** ^***x***^ ligands and assess the impact of intercalated counterions on structural and redox properties.

## Results and Discussion

### Synthesis

The novel ligand **NPC** ^**2**^ was prepared from the literature-reported di-*tert*-butyl­(pyrrolidinyl)­phosphine precursor,^40^ followed by conversion to the imidophosphorane using established methods (Scheme 1).^7,14,28,29^ The 3+ complexes, **1-KPr­(NPC** ^**2**^ **)** and **1-KCe­(NPC** ^**2**^ **)**, were prepared via salt metathesis between LnI_3_THF_4_ (Ln = Pr, Ce) and four equivalents of the potassium salt of the ligand, **KNPC** ^**2**^, in diethyl ether, yielding crystalline solids in 64% and 94%, respectively. Oxidation of **1-KCe­(NPC** ^**2**^ **)** with AgI in diethyl ether affords **2-Ce­(NPC** ^**2**^ **)** as a crystalline solid in 53% yield (Figure 2A). See the Supporting Information for detailed experimental conditions and characterization (Figures S1–S32) of all compounds.

**Scheme 1:**
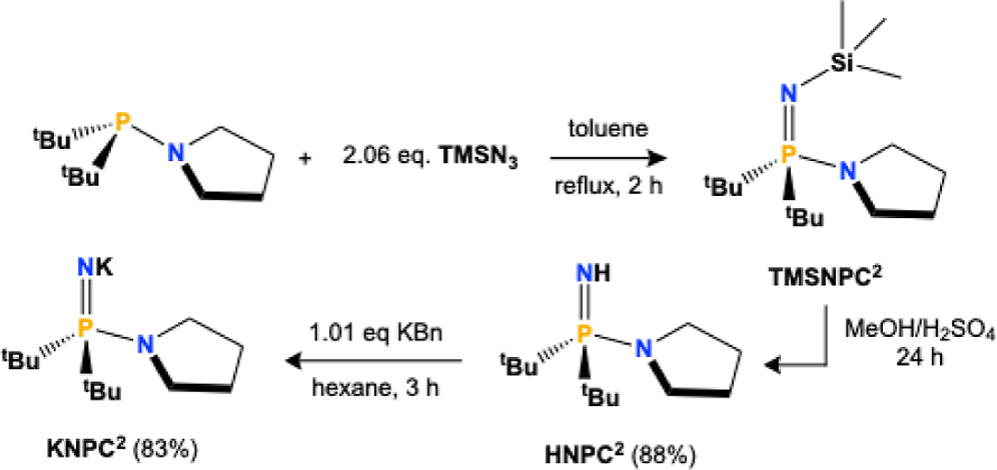
Synthesis of the **HNPC^2^** and **KNPC^2^**

### Structural Analysis

The structures of **1-KPr­(NPC** ^**2**^ **)**, **1-KCe­(NPC** ^**2**^ **)**, and **2-Ce­(NPC** ^**2**^ **)** were crystallographically characterized by single crystal X-ray diffraction (SC-XRD) (Figure S2B–D and Table S2). To quantify deviations from idealized four-coordinate geometries, two complementary metrics were used: (1) the τ_4_ parameter provides a measure of four coordinate geometry, where τ_4_ = 1.00 corresponds to an ideal tetrahedron and τ_4_ = 0.85 to a perfect trigonal pyramidal geometry,^41^ and (2) the Σ_Δ109.5_ parameter quantifies the degree of distortion from ideal tetrahedral geometry using the secondary P coordination sphere.^42^ For trivalent **1-KPr­(NPC** ^**2**^ **)** and **1-KCe­(NPC** ^**2**^ **)**, the τ_4_ values (0.97 and 0.92, respectively) indicate a pseudotetrahedral geometry, while the Σ_Δ109.5_ values (12(3)° and 24(5)°, respectively) reveal additional distortion arising from the incorporation of the potassium cation in the secondary P coordination sphere (Table 1). The potassium cation binds to the imido nitrogen atoms and is further supported by a coordinated diethyl ether molecule. The binding of the potassium cation leads to a bent Ln–N–P linkage with angles ranging from 137.3(2)° to 175.7(3)°. A comparable coordination environment is observed in **1-CsPr­(NPC** ^**3**^ **)**,^15^ whereas **1-KPr­(NP** ^*^ **)** and **1-KCe­(NP** ^*^ **)** exhibit two-coordinate potassium ions bridged by imido nitrogen atoms.^22,25^ The Ln–N bonds fall into two distinct categories: Ln–N_capped_, bound to the alkali metal, and Ln–N_terminal_, coordinated solely to the metal center. For example, in **1-KCe­(NPC** ^**2**^ **)**, the average bond length of Ln–N_capped_ = 2.34(5) Å and Ln–N_terminal_ = 2.32(4) Å. The P–N_imido_ bond length averages of 1.54(2) Å are consistent with values observed in other 3+ lanthanide imidophosphorane complexes.^7,15,22,25,28^ In contrast to the Ln^3+^ complexes, the Ce^4+^ complex, **2-Ce­(NPC** ^**2**^ **)**, approaches ideal tetrahedral geometry with τ_4_ = 0.96 (Table 1). Notably, the Ce–N–P angles remain comparable to those in **1-KCe­(NPC** ^**2**^ **)**, suggesting minimal structural rearrangement upon oxidation.^7^ The P–N_imide_ bond lengths in **1-KCe­(NPC** ^**2**^ **)** and **2-Ce­(NPC** ^**2**^ **)** are within experimental uncertainty (1.57(4) Å vs. 1.55(1) Å, respectively), indicating no statistically significant structural change upon oxidation.^7^ The mean Ce–N bond length contracts by ∼0.17 Å, consistent with the difference in Shannon ionic radii between Ce^3+^ and Ce^4+^ (i.e., 1.01 Å vs. 0.87 Å for 6-coordinate molecules).^43^

**Table 1:** Comparison of Average Metrical Parameters of **1-KPr­(NPC** ^**2**^ **)**, **1-CsPr­(NPC** ^**3**^ **)**, **2-Pr­(NPC** ^**3**^ **)**, **3-Pr­(NPC** ^**3**^ **)**, **1-KCe­(NPC** ^**2**^ **)**, **2-Ce­(NPC** ^**2**^ **)**, **1-CsCe­(NPC** ^**3**^ **)**, and **2-Ce­(NPC** ^**3**^ **)** Determined by SC-XRD^1,15^

	1-KPr(NPC^2^)	1-CsPr(NPC^3^)	2-Pr(NPC^3^)	3-Pr(NPC^3^)	1-KCe(NPC^2^)	2-Ce(NPC^2^)	1-CsCe(NPC^3^)	2-Ce(NPC^3^)
Ln–N_capped_ (Å)	2.31(1)	2.33(1)	-	-	2.34(5)	-	2.333(2)	-
Ln–N_term_ (Å)	2.29(6)	2.313(2)	-	-	2.32(4)	-	2.35(1)	-
Ln–N (Å)	2.31(2)	2.33(1)	2.179(3)	2.25(5)	2.33(4)	2.16(4)	2.34(1)	2.176(4)
Ln–P (Å)	3.75(5)	3.81(3)	3.69(1)	3.80(3)	3.73(10)	3.67(5)	3.82(4)	3.70(1)
N–P (Å)	1.53(2)	1.552(1)	1.561(1)	1.57(1)	1.55(1)	1.57(4)	1.552(2)	1.562(3)
N–Ln–N (°)	109.4(4)	109(11)	109.5(21)	109(6)	108(15)	109(2)	109(11)	109.5(16)
Ln–N–P (°)	158(1)	160(1)	162(1)	166(3)	152(18)	158(7)	160(12)	163(1)
τ_4_	0.97	0.98	0.98	1.00	0.92	0.96	0.99	1.00
∑_109.5_Θ_P–Ln–P_(°)	12(3)	5(1)	9(2)	8(2)	24(5)	19(4)	5(1)	6(2)

The binding of alkali metals in Ln^3+^ imidophosphorane complexes significantly impacts oxidation potential (vide infra). **NPC** ^**3**^ complexes were crystallographically characterized as cesium salts, whereas **NPC** ^**2**^ complexes were directly obtained as potassium adducts from the ligand salt. Both systems feature a three-coordinate alkali cation, a motif observed in other Ln^3+^ imidophosphorane complexes, where the three-coordinate bridged alkali metal reduces electron donation to the metal, mitigating destabilization of the *f*-orbital manifold.^7^ We propose that the steric bulk, as well as the high symmetry, of **NPC** ^**3**^ reduces structural reorganization upon oxidation, reflected by the minimal structural changes observed in geometry between the Ln^3+^ and Ln^4+^ complexes.^15^ Additionally, in Ce^3+^ and Pr^3+^ **NPC** ^**3**^ complexes, the Cs^+^ counterion resides further from the metal and primarily functions as an outer sphere ion, in contrast to K^+^ in the corresponding **NPC** ^**2**^ complexes, resulting in weaker electrostatic interactions between the counterion and the anionic ligand framework.

### Electrochemical Analysis

Cyclic voltammetry (CV) measurements were performed in 0.1 M [^*n*^Bu_4_N]­[BPh_4_] in THF and referenced to Fc^0/+^ (see Supporting Information for experimental considerations). The redox potentials of complexes **1-KPr­(NPC** ^**2**^ **)**, **1-KCe­(NPC** ^**2**^ **)**, and **2-Ce­(NPC** ^**2**^ **)** were measured using CV (Figures S27–S29). The Ce^4+/3+^ couple of **2-Ce­(NPC** ^**2**^ **)** exhibits irreversible behavior, similar to **2-Ce­(NPC** ^**3**^ **)** (Figure 3). The redox potentials for imidophosphorane Ce^4+^ compounds (Table S1) all occur at cathodic potentials (−0.16 V to −3.14 V). However, the Ce complexes supported by the NP* ligands exhibit the most positive potentials, reflecting their distinctly less electron-donating character. The oxidation and reduction potentials of Ce^4+^ complexes, in Table S1, supported by **NPC** ^**1**^, **NPC** ^**2**^, and **NPC** ^**3**^ ligands exist within a narrow range (*E*_pa_ = −2.28 V to −2.33 V; *E*_pc_ = −2.91 V to −3.22 V) and show no linear correlation with the number of ^*t*^Bu substituents, indicating that such variations exert only a minor influence on the relative energy of the *f*-orbital energies. These effects remain ambiguous because the Ce^3+^ analogs feature different counterions, which, as noted earlier, can greatly impact electrochemical potenials.

**Figure 3:**
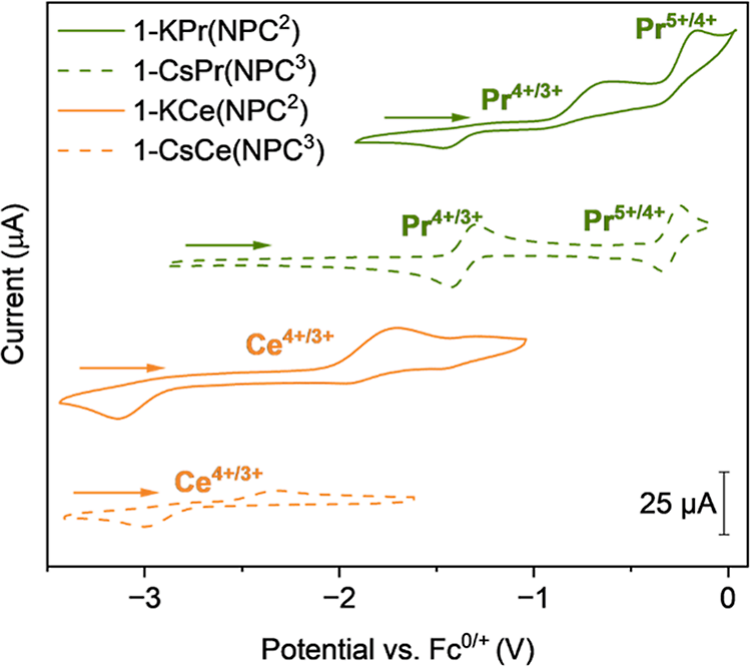
Cyclic voltammograms collected in a 0.1 M [^*n*^Bu_4_N]­[BPh_4_] in THF. Analyte concentration is 3 mM for **1-KPr­(NPC** ^**2**^ **)** and **1-KCe­(NPC** ^**2**^ **)**, and 1 mM for **1-CsPr­(NPC** ^**3**^ **)** and **1-CsCe­(NPC** ^**3**^ **)** at 200 mV/s. All potentials are referenced against Fc^0/+^.^1^

The influence of the alkali metal on oxidation potential has been well studied within the **NP** ^*^ framework.^27^ However, in the **NPC** ^**2**^ and **NPC** ^**3**^ systems, a direct comparative series was not established across alkali metals. In contrast to the studies of Ce **NP** ^*^ complexes, it is not established whether these alkali metal interactions persist in solution for **1-KCe­(NPC** ^**2**^ **)** and **1-CsCe­(NPC** ^**3**^ **)** with the data at hand. However, there is a large cathodic shift in the *E*_pa_ for **1-CsCe­(NPC** ^**3**^ **)** compared to **1-KCe­(NPC** ^**2**^ **)** (−2.26 V vs −1.69 V), suggesting that the identity of the counterion plays a role in the electrochemical properties of these molecules.

The Pr^4+/3+^ and Pr^5+/4+^ couples for **1-KPr­(NPC** ^**2**^ **)** exhibit irreversible behavior. The electrochemical redox potentials for the Pr^4+/3+^ couple of **1-KPr­(NPC** ^**2**^ **)** are *E*_pa1_ = −0.60 V and *E*_pc1_ = −1.47 V (*E*_pa1/pc1_ = first redox event), and for the Pr^5+/4+^ couple are an *E*_pa2_ = −0.16 V and an *E*_pc2_ = – 0.33 V (*E*_pa2/pc2_ = second redox event). In contrast, **1-CsPr­(NPC** ^**3**^ **)** exhibits quasi-reversible Pr^4+/3+^ and Pr^5+/4+^ redox couples but displays similar redox potentials (Table 2).^1^ Notably, the redox potentials for the Pr^4+/3+^ and Pr^5+/4+^ couples in **1-KPr­(NPC** ^**2**^ **)** are shifted to more positive values, suggesting that higher oxidation states are less accessible, which is in agreement with the irreversible CV waves and chemically unstable products. Much like the Ce^4+/3+^ couple, the *E*_pa_ value for the Pr^4+/3+^ couple in the **NPC** ^**2**^ complex is much more positive than the **NPC** ^**3**^ complex (i.e., −0.60 V vs. −1.26 V), suggesting a similar effect from the counterion.

**Table 2:** Electrochemical Potentials (V) for **1-Ln­(NPC** ^***x***^ **)** (*x* = 1, 2, 3) and **1-Ln­(NP** ^*^ **).** ^a^

	*E*_pa_	*E*_pc_	*E*_1/2_
**1-KPr(NPC** ^**2**^ **)**	–0.60/–0.16	–1.47/–0.33	–
**1-CsPr(NPC** ^**3**^ **)** (ref 1)	–1.26/–0.24	–1.45/–0.35	–1.35/–0.29
**1-KCe(NPC** ^**2**^ **)**	–1.69	–3.14	–
**1-CsCe(NPC** ^**3**^ **)** (ref 1)	–2.26	–3.01	–
**1-KCe(NP** ^*^ **)** (ref 25)	–1.44	–2.88	–

a^1,25^ Values before and after the slash correspond to the Pr^4+/3+^ and Pr^5+/4+^ couples, respectively (see Table S1 to view all redox potentials, including **2-Ln­(NPC** ^***x***^ **)** and **2-Ln­(NP** ^*^ **)**).

### Chemical Oxidation of 1-KPr­(NPC^2^)

Attempts to oxidize **1-KPr­(NPC** ^**2**^ **)** with 2 equiv of FcBArF_20_ in d_8_-THF were monitored by nuclear magnetic resonance (NMR). Following the reaction by ^31^P­{^1^H} NMR reveals resonances corresponding to **1-KPr­(NPC** ^**2**^ **)** (δ 268 ppm) and a second signal assigned to the doubly protonated ligand (δ 65 ppm) (Figure S25). Confirmation of the identity of this species was made by independent synthesis of **H**_**2**_ **NPC** ^**2**^. Consistent with the decomposition of the **NPC** ^**3**^ supported Pr^5+^ complex,^1^ attempts to generate **3-Pr­(NPC** ^**2**^ **)** in d_8_-THF revealed rapid degradation by ligand disproportionation into **2-Pr­(NPC** ^**2**^ **)** and the doubly protonated ligand **H**_**2**_ **NPC** ^**2**^. The spectroscopic evidence indicates that Pr^4+^(NPC^2^) is inaccessible and decomposes through either hydrogen atom transfer (HAT) or proton-coupled electron transfer (PCET) from solvent followed by ligand disproportionation, which parallels decomposition of the reported Pr^5+^ complex (Scheme S1 and Figures S24–S26).^1^

For a given central metal ion and oxidation state, the Ln–N bond lengths differ by less than ∼0.025 Å across the three NPC ligand frameworks, *e.g.*, Ln–N = 2.198 Å in **2-Ce­(NPC** ^**1**^ **)**, 2.203 Å in **2-Ce­(NPC** ^**2**^ **)**, and 2.209 Å in **2-Ce­(NPC** ^**3**^ **)**. From another perspective, the structural differences within the NPC ligand family can be rationalized by quantifying the steric bulk of each ligand. To this end, the G-parameter^44^ was calculated for the DFT optimized structures (Figures 4A and S37, Table S12), a metric that has been successfully applied in previous studies to characterize ligand interactions in organometallic and coordination complexes in terms of the percentage of the metal coordination sphere shielded by a given ligand.^45,46^ Essentially, the G-parameter can quantify the likelihood that an incoming solvent is sterically blocked from accessing the metal center; thus, higher values indicate greater steric protection. Among the three ligands examined across all complexes, **NPC** ^**3**^ consistently exhibits the largest value. For example, in the Pr^5+^ complexes, the G-parameter is 77.5% for the **NPC** ^**1**^ ligand, 79.0% for **NPC** ^**2**^, and 80.5% for **NPC** ^**3**^. Since the Ln–N bond lengths remain largely unchanged for complexes with the same metal ion and oxidation state, the observed increase in the G parameter is primarily attributed to the greater number of ^*t*^Bu groups bound to the P atom. With increased steric protection around the metal center, the N_im_ atoms in the first coordination sphere are also expected to be more shielded from solvent molecules. As previously shown for the Np^5+^/Pu^5+^ complexes supported by **NPC** ^**1**^, THF can participate in PCET reactions via protonation of the N_im_ atoms and reduction of the metal center.^47,48^ The enhanced steric protection provided by the ^*t*^Bu groups in **NPC** ^**3**^ also correlates with improved redox reversibility, rendering the Pr^4+/3+^ and Pr^5+/4+^ couples quasi-reversible, in contrast to the irreversible behavior observed with **NPC** ^**2**^.

**Figure 4:**
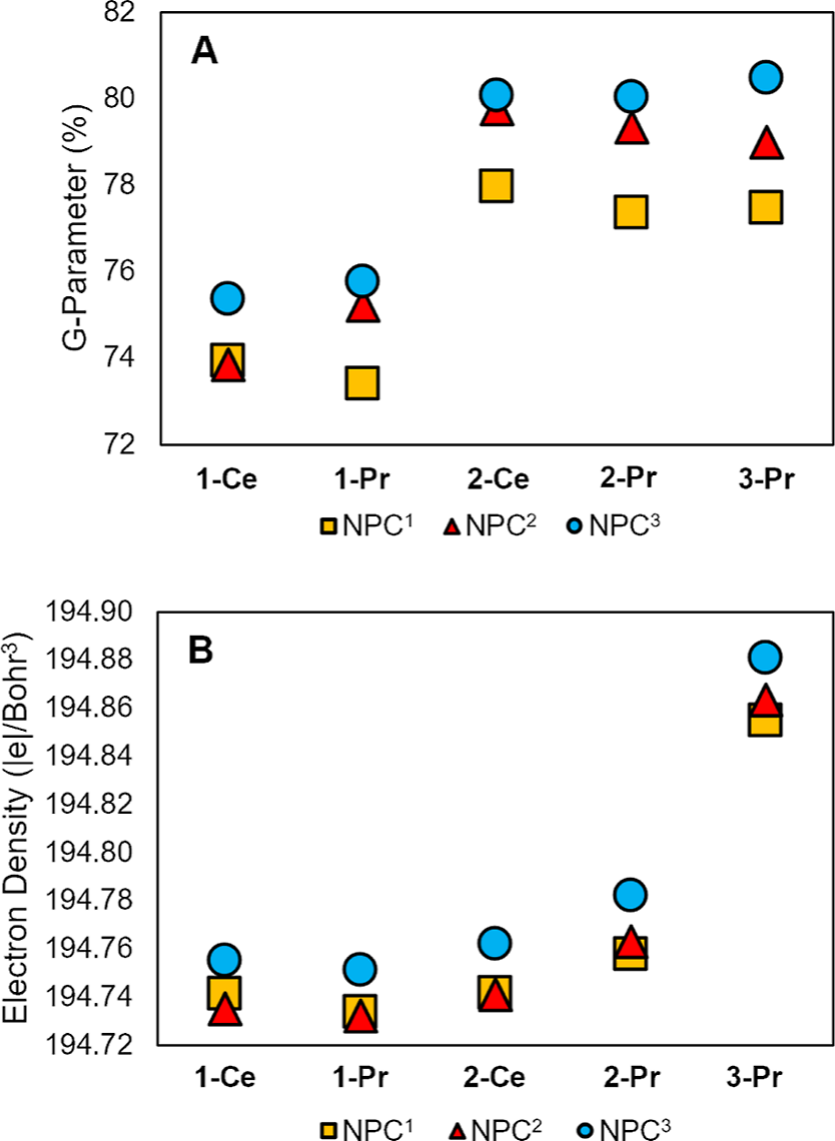
(A) G-Parameter and (B) the electron density at the N_im_ nuclear critical point calculated for **Ln­(NPC** ^**x**^ **)** complexes. See Figure S37 for G-parameter visualizations of selected complexes and Tables S12 and S15 for detailed values.

### DFT Calculations

Due to variations in steric profiles and the presence of different counterions in the 3+ oxidation state, it is challenging to directly assess the electron-donating ability of the **NPC** ^***x***^ (*x* = 1, 2, 3) ligands from experimental data alone. Moreover, comparison of the bond metrics across the **NPC** ^***x***^ series does not reveal clear structural differences among the oxidized products, as the complexes exhibit comparable coordination environments. This indicates that structural metrics alone do not account for the observed differences in the **NPC** ^***x***^ series’ ability to access higher Ln oxidation states, suggesting underlying changes in electronic structure upon oxidation in addition to the steric effects. To address these uncertainties and systematically evaluate all complexes, DFT calculations were carried out (see Supporting Information for Computational Details). Specifically, we computed the experimental **1-KPr­(NPC** ^**2**^ **)**, **1-KCe­(NPC** ^**2**^ **)**, and **2-Ce­(NPC** ^**2**^ **)** compounds, as well as theoretically modeled complexes **Pr** ^**4+**^ **(NPC** ^**2**^ **)** and **Pr** ^**5+**^ **(NPC** ^**2**^ **)**, and their respective **NPC** ^**1**^ and **NPC** ^**3**^ analogs, some of which have been reported previously (Tables S3–S5).^1,26^ For consistency across this set of models, the Ln^3+^ complexes were initially optimized without the counterion (additional models incorporating the counterion were also considered, *vide infra*). The optimized structures show good geometric agreement with the experimentally characterized systems, with errors of less than 1.9% for Ln–N and N–P bond lengths and less than 2.0% for τ_4_ values, supporting the reliability of the computational methodology.

Natural population analysis charges (Q_NPA_) on N_im_ show minimal variation of 0.01–0.03 *e* across the **NPC** ^**1**^, **NPC** ^**2**^, and **NPC** ^**3**^ ligands for a given oxidation state and metal identity (Figures S38–S39, Tables S13–S14). The N_im_ Q_NPA_ becomes slightly more positive with increasing oxidation state of Ln, *e.g.*, shifting from −1.59 in **1-Pr­(NPC** ^**3**^ **)** to −1.47 in **2-Pr­(NPC** ^**3**^ **)**, and further to −1.33 in **3-Pr­(NPC** ^**3**^ **)**. Conversely, the Ln Q_NPA_ slightly decreases upon oxidation, with the **NPC** ^**3**^ ligand yielding the lowest values, suggesting it is the most electron-donating ligand among those studied. This trend aligns with previous findings by Suresh and co-workers, who identified the ^*t*^Bu group as one of the most electron-donating substituents for phosphine ligands due to its favorable stereoelectronic profile.^49,50^ The enhanced steric protection provided by the ^*t*^Bu groups in **NPC** ^**3**^ is accompanied increased electron density at the N_im_ nuclear critical points (ρ @ N_im_ NCP) across all oxidation states, as determined by quantum theory of atoms in molecules (QTAIM) analysis (Figure 4B and Table S15). In previous works, we demonstrated that in **NPC** ^**1**^ complexes of U, Np, and Pu, the ρ @ N_im_ NCP correlates with the thermodynamic drive for PCET.^47,48^ The current QTAIM results further reveal that the ρ @ N_im_ NCP in **NPC** ^**3**^ increases more significantly with increasing metal oxidation state compared to the corresponding values in **NPC** ^**1**^ and **NPC** ^**2**^. A comparison of the Ce^4+^ and Pr^4+^ complexes shows slightly higher ρ @ N_im_ NCP values for the Pr species regardless of the NPC ligand framework, e.g., for **NPC** ^**3**^: 194.762 |e|/Bohr^3^ *vs.* 194.782 |e|/Bohr^3^. Notably, the ρ @ N_im_ NCP values in the **3-Pr­(NPC** ^**x**^ **)** complexes (194.855–194.881 |e|/Bohr^3^) are significantly greater than in any of the Ln^4+^ analogs (194.741–194.782 |e|/Bohr^3^), consistent with the higher reactivity observed for the Pr^5+^ species.

Molecular orbital (MO) energy diagrams provided further insight into the electronic properties of the NPC ligands (Figures S40–S44). A distinct trend emerges in the energies of the lowest unoccupied molecular orbitals (LUMOs) across the ligand series. In all cases, the metal-dominant LUMO is found to be highest in energy for **NPC** ^**3**^, regardless of the identity or oxidation state of the Ln, *e.g.*, Pr^4+^: −3.06 eV (**NPC** ^**1**^) *vs.* −3.07 (**NPC** ^**2**^) *vs*. −2.93 eV (**NPC** ^**3**^). This result suggests that the reduction of the metal center is slightly less favorable in **NPC** ^**3**^. While the highest occupied molecular orbital (HOMO) energies show no consistent trend across the complexes, a notable pattern is observed in the energy gap between the lowest singly occupied 4*f* orbital and the highest-energy ligand-dominant orbital. Previous studies proposed that the smaller 4*f*–ligand orbital energy gap may contribute to the enhanced stability of U^3+^ complexes.^51^ In the present Ce^3+^ and Pr^3+^ species, **NPC** ^**3**^ consistently exhibits the smallest 4*f*–ligand energy gap, e.g., Ce: 2.42 eV (**NPC** ^**1**^), 2.35 eV (**NPC** ^**2**^), 2.22 eV (**NPC** ^**3**^), suggesting enhanced electronic stability for the **NPC** ^**3**^-ligated complexes. In the Pr^4+^ complexes, the singly occupied 4*f*-dominant orbital shifts below the ligand-dominant MOs (Figure S43). Notably, this 4*f*-dominant orbital of **2-Pr­(NPC** ^**3**^ **)** lies slightly higher in energy than that in **2-Pr­(NPC** ^**1**^ **)** or **2-Pr­(NPC** ^**2**^ **)**, resulting in the smallest energy gap (1.10 eV *vs*. 1.08 eV *vs*. 1.03 eV). The reduced gap may contribute to the accessibility of the Pr^5+^ species, consistent with the slightly more negative calculated Pr^5+/4+^ redox potential: −0.30 V for **NPC** ^**3**^ compared to −0.24 V for **NPC** ^**2**^ and −0.20 V for **NPC** ^**1**^ (Figure S45 and Table S16). From a thermodynamic perspective, these findings suggest that the **NPC** ^**3**^ ligand provides a modest thermodynamic increase in stabilization of higher oxidation states relative to **NPC** ^**1**^ and **NPC** ^**2**^.

To evaluate the effects of intercalated alkali metal ions, all complexes were further optimized with either K^+^ or Cs^+^ cations in the form **[M** ^**+**^ **]­[Ln­(NPC** ^***x***^ **)]** (M = K, Cs; Ln = Ce, Pr; *x* = 1, 2, 3) (Tables S6–S11) with subsequent redox potential calculations. These calculations were performed under the assumption that the alkali metal cation remains associated with the complex throughout the redox processes on the electrochemical time scale (Figures S46–S48, Table S17). The computed Ln^4+/3+^ redox potentials for **1-KLn­(NPC** ^**2**^ **)** closely match the experimental *E*_pa_ values (Ce: −1.71 V (theor.) *vs*. −1.69 V (exp.); Pr: −0.68 V (theor) *vs*. −0.60 V (exp.)), supporting the K^+^ retention upon oxidation. In contrast, the computed Ln^4+/3+^ redox potentials for **1-CsLn­(NPC** ^**3**^ **)** show substantial deviations from the experimental *E*_pa_ values (Ce: −1.71 V (theor.) *vs*. −2.26 V (exp.); Pr: −0.70 V (theor.) *vs*. −1.26 V (exp.)), suggesting that Cs^+^ is likely ejected during the oxidation process. These findings are consistent with our previous study on **Ce­(NP** ^*^ **)** complexes, which also demonstrated retention of smaller and loss of larger alkali metal cations during oxidation. Comparing the three NPC ligands in **1-Ln­(NPC** ^***x***^ **)**, the redox potentials vary by only 0.14 V for both Ce and Pr, becoming slightly more positive from **NPC** ^**1**^ to **NPC** ^**3**^. This suggests slightly greater electronic stabilization in the **1-KLn­(NPC** ^**3**^ **)** complexes. This trend is consistent with the MO diagrams of **1-KLn­(NPC** ^***x***^ **)** (Figures S49 and S50), which show that the 4*f*-dominant orbitals gradually stabilize by 0.09–0.12 eV from **NPC** ^**1**^ to **NPC** ^**3**^. Additionally, when compared to the systems without counterions, it can be seen that K^+^ intercalation lowers the HOMO energy in **[K** ^**+**^ **]­[1-Ln­(NPC** ^**x**^ **)]** by 0.71–0.73 eV for both Ce and Pr analogs. A similar trend is observed with Cs^+^ as the intercalated counterion (Figures S51 and S52).

The calculated Ln^4+/3+^ redox potentials for the cation-free **Ln­(NPC** ^**x**^ **)** complexes (Table S16) are much closer to the experimentally observed values for the complexes containing intercalated Cs^+^. For example, the computed Ce^4+/3+^ redox potential for **Ce­(NPC** ^**3**^ **)** is −2.57 V, which falls between the experimental *E*_pa_ (−2.26 V) and *E*_pc_ (−3.01 V) for **1-CsCe­(NPC** ^**3**^ **)**. This result suggests that Cs^+^, which resides at a greater distance from the Ln center, is more readily ejected during oxidation, consistent with the more negative redox potentials observed compared to the K^+^-containing systems. Although the HOMO stabilization in the Cs^+^-intercalated species is comparable to that in the K^+^ analogues, the larger Cs^+^ ion is more favorable for stabilizing these systems as it leads to more negative redox potentials.

The calculated vertical/adiabatic detachment energies (VDE/ADE) and vertical/adiabatic electron affinities (VEA/AEA) benchmark the degree of structural rearrangement associated with the redox processes (vertical, none; adiabatic, total) (Table S18). For the Ln^4+/3+^ couples of the cation-free **Ln­(NPC** ^***x***^ **)** complexes, the differences between the vertical and adiabatic processes are generally largest for complexes with the **NPC** ^**1**^ ligand and smallest for those with **NPC** ^**3**^. For example, in Ce^4+/3+^, |VDE – ADE| decreases from 0.68 V (**NPC** ^**1**^) to 0.57 V (**NPC** ^**2**^) and 0.49 V (**NPC** ^**3**^). This indicates that complexes with the **NPC** ^**1**^ ligand undergo greater structural reorganization upon oxidation, likely due to the increased flexibility of the pyrrolidinyl rings. In contrast, the bulky ^*t*^Bu groups in **NPC** ^**3**^ impose greater rigidity on the secondary coordination sphere, limiting structural rearrangement. Inclusion of K^+^ or Cs^+^ preserves this pattern while slightly reducing |VDE–ADE| by an average of 0.03–0.08 V across the considered complexes. Overall, these results indicate that, for complexes retaining the alkali metal, its coordination enforces an asymmetric geometry for the Ln^3+^ species, whereas the Ln^4+^ analogue remains highly symmetric, thereby minimizing the extent of structural reorganization upon oxidation. This effect is more pronounced for the sterically rigid **NPC** ^**3**^ ligand, which exhibits the smallest structural response relative to the more flexible **NPC** ^**1**^ analogue.

To assess the thermodynamic stabilization afforded by the alkali cations, we calculated the energy required to eject K^+^ and Cs^+^ from **1 M** ^**+**^ **Ln­(NPC** ^***x***^ **)** following the dissociation pathway [Ln^q+^(NPC^*x*^)]­[M^+^] → [Ln^q+^(NPC^x^)] + [M^+^] (Figure 5 and Table S19). As only implicit solvation was included through polarized continuum model and no explicit solvent molecules were coordinated, the analysis focuses on relative energy values rather than absolute energies. The results show that retaining K^+^ is thermodynamically more favorable than retaining Cs^+^ by 10.4–12.7 kcal/mol for Ce^3+^ and 11.1–12.9 kcal/mol for Pr^3+^ across the three NPC ligands. Although Cs^+^ binding remains more favorable in all Ce^3+^ and Pr^3+^ complexes of **1-CsLn­(NPC** ^**x**^ **)** compared to their Ln^4+^ counterparts, it is notably easier to dissociate Cs^+^ from the **NPC** ^**3**^ ligand compared to **NPC** ^**1**^ and **NPC** ^**2**^, with energy differences of 3.0–6.9 kcal/mol in Ln^3+^ complexes. This trend likely arises from the partial negative charge on the nitrogen atoms in the pyrrolidine rings of **NPC** ^**1**^, which enhances interaction with the Cs^+^ cation. A similar trend is observed in the **1-KLn­(NPC** ^**x**^ **)** series, though with slightly smaller energy differences across the three ligands, 0.7–5.1 kcal/mol. Likewise, in the 4+ oxidation state, **2-Ln­(NPC** ^***x***^ **)**, ligands with pyrrolidine substituents (**NPC** ^**1**^ and **NPC** ^**2**^) exhibit relatively more favorable interactions with the cation than **NPC** ^**3**^, by 4.7–9.2 kcal/mol for K^+^ and 8.0–11.5 kcal/mol for Cs^+^. Taken together, these results suggest that the **NPC** ^**3**^ ligand offers the most favorable thermodynamic profile for cation ejection regardless of the oxidation state or metal identity.

**Figure 5:**
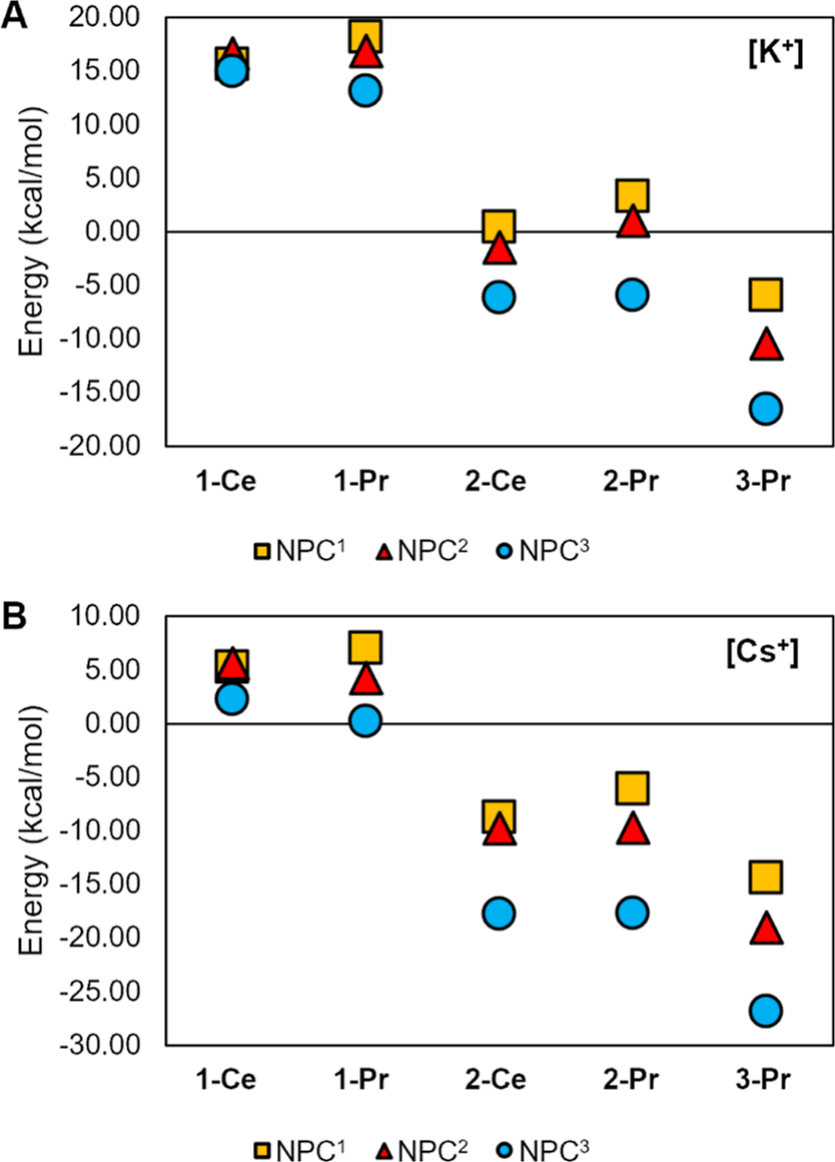
Energy (kcal/mol) required to eject (A) [K^+^] and (B) [Cs^+^] in the dissociation pathway [Ln^q+^(NPC^*x*^)]­[M^+^]→ [Ln^q+^(NPC^x^)] + [M^+^]. See Table S19 for detailed values.

## Conclusions

Achieving high-oxidation-states in lanthanide and actinide compounds requires a careful balance of electron donation, steric protection, and ligand redox stability. The strongly electron-donating NPC imidophosphorane ligand class can effectively support complexes featuring electron-deficient Ce and Pr centers. Ligand substitutions within the **NPC** ^***x***^ (*x* = 1, 2, 3) family result in subtle variations in redox potentials, differing by no more than 0.14 V for the Ln^4+/3+^ couples and 0.10 V for the Pr^5+/4+^ couples across the three ligands. This result aligns with the modest impact these substitutions have on the relative energies of the *f*-orbital manifold. Among them, **NPC** ^**3**^ stands out as the best ligand for stabilizing higher oxidation states due to a combination of electronic and steric factors. From a thermodynamic perspective, **NPC** ^**3**^ exhibits only slightly more negative Pr^5+/4+^ redox potentials, indicating somewhat easier oxidation compared to **NPC** ^**1**^ and **NPC** ^**2**^. Geometrically, the bulky ^*t*^Bu groups of **NPC** ^**3**^ provide enhanced steric protection, which may reduce undesired interactions with solvent molecules despite the ligand’s highest electron density at the N_im_ center across the three **NPC** ^**x**^ ligands. The enhanced steric profile of **NPC** ^**3**^ may also contribute to the improved reversibility of the Pr^4+/3+^ and Pr^5+/4+^ couples compared to **NPC** ^**2**^.

Our results also reinforce the critical role of counterions in modulating the redox behavior of the Ln^4+/3+^ couples. Consistent with previous studies, we demonstrate that alkali metal cations in **NPC** ^**x**^-stabilized complexes contribute to the electronic stabilization and significantly influence oxidation potentials, depending on their size and proximity to the metal center.^27,36,38^ Larger counterions, which tend to occupy the outer coordination sphere, shift redox potentials to more negative values, thereby facilitating more facile metal-centered oxidation. Additionally, the bulky alkyl substituents in **NPC** ^**3**^ and lack of secondary nitrogen sites to bind alkali metals enable easier counterion dissociation compared to **NPC** ^**1**^ and **NPC** ^**2**^ analogues, with minimal structural reorganization observed upon Ln^4+/3+^ oxidation.

## Experimental Details

### General Considerations

Unless otherwise noted, all reagents were obtained from commercial suppliers, and all syntheses and manipulations were conducted with the exclusion of oxygen and water using Schlenk techniques under Ar or in a N_2_ filled glovebox (Vigor, <0.1 ppm of O_2_/H_2_O). Benzyl potassium (KBn),^52^ di-*tert*-butyl­(pyrrolidinyl)­phosphine,^40^ and [H­(OEt_2_)]­[BArF_20_],^47^ CeI_3_(THF)_4_, and PrI_3_(THF)_4_ ^53^ were prepared as previously described. Further considerations and experimental details can be found in the Supporting Information

### Caution!

Trimethylsilyl azide (TMSN_3_) is toxic and explosive under certain conditions and should be handled in a well-ventilated fume hood or a glovebox with appropriate personal protective equipment (PPE).

### HNP­(^*t*^Bu)_2_(pyrr) (HNPC^2^)

Inside a glovebox, (^*t*^Bu)_2_(pyrr)P (3.512 g, 16 mmol) was transferred to a 100 mL Schlenk pear flask equipped with a Teflon stir bar and dissolved in 40 mL of toluene. Trimethylsilyl azide (4.3 mL, 33 mmol) was then added to the solution, and the flask was sealed. The flask was transferred to a Schlenk line, and the reaction mixture was stirred at reflux for 2 h. Volatiles were removed in vacuo to give a turbid, colorless oil. Inside a glovebox, the crude material was extracted in 20 mL of hexane and filtered through a fine-porosity frit packed with Celite. Volatiles were removed in vacuo from the colorless filtrate to yield the TMSNPC^2^ intermediate as a colorless oil. The intermediate was analyzed via NMR (see below) and was determined to be of sufficient purity to proceed to the second step. ^1^H NMR (400 MHz, C_6_D_6_): δ 3.09 (t, 4H), 1.43 (t, 4H), 1.15 (d, 8H), 0.38 (s, 9H). ^13^C­{^1^H} NMR (101 MHz, C_6_D_6_): δ 49.19, 39.69, 38.91, 28.07, 26.36, 4.76. ^31^P­{^1^H} NMR (162 MHz, C_6_D_6_): δ 29.75. Inside a glovebox, the oil was transferred to a Schlenk pear flask equipped with a Teflon stir bar. The vessel was cycled onto a Schlenk line and 25 mL of methanol (0.62 mol) was added via cannula, followed by 2 drops of concentrated, aqueous H_2_SO_4_. The reaction mixture was sealed and then stirred at room temperature for 24 h. The volatiles were then removed in vacuo to give the crude product as a colorless oil. The oil was transferred to a vial in the glovebox, and 2 mL of pentane and 2 mL of diethyl ether were added sequentially to give a cloudy, white suspension. The vial was placed in a −35 °C freezer overnight, during which time XRD-quality colorless crystals grew. The supernatant was decanted off, and the residual volatiles were removed in vacuo to give the title compound as a white crystalline solid (3.30 g, 88%). On warming to room temperature, the material becomes waxy. ^1^H NMR (500 MHz, C_6_D_6_): δ 3.70 (s, 1H), 3.13 (t, 4H), 1.43 (t, 4H), 1.21 (d, 18H). ^13^C­{^1^H} NMR (126 MHz, C_6_D_6_): δ 49.70, 39.06, 38.51, 28.10, 26.53. ^31^P­{^1^H} NMR (203 MHz, C_6_D_6_): δ 55.34. IR (ATR) ν [cm^–1^] = 2958 (s), 2901 (s), 2869 (s), 1977 (vw), 1589 (w), 1476 (m), 1399 (w), 1373 (w), 1295 (vw), 1257 (vw), 1200 (m), 1146 (m), 1124 (m), 1076 (vs), 1016 (vs), 991 (s), 962 (m), 945 (m), 872 (vw), 814 (s), 755 (vw), 725 (vw), 645 (m), 602 (s), 580 (m), 524 (m), 465 (m). Elemental analysis, C_12_H_27_N_2_P, found (calculated): C 63.02 (62.57), H 11.25 (11.82), N 12.32 (12.16).

### KNP­(^*t*^Bu)_2_(pyrr) (KNPC^2^)

Inside a glovebox, HNPC^2^ (575.8 mg, 2.50 mmol) was dissolved in 10 mL toluene. Then potassium benzyl (341.9 mg, 2.63 mmol) was added as a solid, and the mixture was stirred for 3 h. During the course of the reaction, a dark orange/brown precipitate was formed. The precipitate was filtered away on a coarse-porosity frit and washed with toluene (5–10 mL). The combined filtrates were concentrated to 5 mL in vacuo and cooled overnight in −35 °C freezer to yield colorless crystals of the title complex (581.6 mg, 83%). ^1^H NMR (400 MHz, THF-*d*_*8*_): δ 3.34 (t, 4H), 1.65 (t, 4H), 1.18 (d, 18H). ^13^C­{^1^H} NMR (101 MHz, THF-*d*_*8*_): δ 48.87, 39.77, 39.19, 29.23, 25.72. ^31^P­{^1^H} NMR (162 MHz, THF-*d*_*8*_): δ 10.76. IR (ATR) ν [cm^–1^] = 2942 (m), 2885 (m), 2856 (m), 2034 (vw), 1977 (vw), 1496 (vw), 1475­(vw), 1455 (vw), 1384 (vw), 1375 (vw), 1351 (vw), 1340 (vw), 1285 (vw), 1188 (m), 1153 (vs), 1127 (m), 1048 (m), 986 (s), 932 (w), 804 (s), 732 (m), 697 (m), 612 (s), 581 (s), 519 (m), 475 (m). Elemental analysis, KC_13.75_H_28_N_2_P, found (calculated): C 56.71 (56.66), H 9.63 (9.68), N 9.78 (9.61).

### [H_2_NP­(^*t*^Bu)_2_(pyrr)]­[BArF_20_] (H_2_NPC^2^)

Inside the glovebox, HNPC^2^ (46 mg, 198 μmol) was dissolved in 2 mL Et_2_O in a 20 mL scintillation vial charged with a Teflon stir bar. [H­(OEt_2_)_2_]­[BArF_20_] (164 mg, 198 μmol) was dissolved in 3 mL of Et_2_O and added to the reaction vial, and the reaction mixture was stirred for 1 h. Volatiles were removed in vacuo, resulting in white solids. The solid was triturated with *n*-pentane (1 mL × 3). The solid was brought up in 1 mL of Et_2_O and filtered through a pipet packed with Celite and glass fiber filter paper. The solution was concentrated in vacuo to 0.2 mL and placed in a −35 °C freezer overnight to yield clear needle XRD quality crystals (174 mg, 95%). ^1^H NMR (400 MHz, THF-*d*_*8*_): δ 1.45 (d, 18H), 1.95 (t, 4H), 3.44 (t, 4H), 4.97 (s, 2H). ^13^C­{^1^H} NMR (101 MHz, THF-*d*_*8*_): δ 50.87, 39.83, 39.20, 27.06.^19^F­{^1^H} NMR (376 MHz, THF-*d*_*8*_): δ −133.06, −165.50, −168.89. ^31^P­{^1^H} NMR (162 MHz, THF-*d*_*8*_): δ 69.25. IR (ATR) ν [cm^–1^] = 2942 (s), 2862 (s), 2804 (m), 1643 (w), 1591 (vw), 1513 (m), 1491 (w), 1462 (s), 1370 (m), 1275 (w), 1251 (w), 1209 (w), 1103 (s), 996 (m), 978 (s), 910 (w), 812 (vw), 774 (w), 756 (w), 746 (vw), 684 (w), 661 (w), 603 (vw), 563 (m). Elemental analysis, C_44_H_40_BF_24_N_2_P, found (calculated): C 48.45 (48.28), H 3.71 (3.68), N 2.84 (2.56).

### K­[Ce^3+^NP^*t*^Bu_2_pyrr]_4_ (1-KCe­(NPC^2^))

Inside a glovebox, CeI_3_THF_4_ (151 mg, 187 μmol, 1.0 equiv) was added to a 20 mL scintillation vial charged with a glass stir bar and 1 mL diethyl ether was then added. KNPC^2^ (201 mg, 749 μmol, 4.0 equiv) was added as a solid, and any residual ligand was transferred as slurry in 1 mL diethyl ether. The reaction mixture was stirred overnight and filtered through a pipet filter packed with glass filter paper and Celite. The solvent was concentrated in vacuo, and the resulting pale-yellow oil was triturated with *n*-pentane (1 mL × 3). The residue was taken up in 2 mL of *n*-pentane and filtered through a pipet packed with Celite and glass fiber filter paper. The solution was concentrated in vacuo and then 5–10 drops of diethyl ether were added, and the solution was then placed in a −35 °C freezer overnight, during which time pale-yellow XRD quality crystal grew (194 mg, 94%). ^1^H NMR (400 MHz, THF-d_8_): δ 0.59 (s, 16H), 0.30 (d, 72H), −0.68 (s, 16H). ^13^C­{^1^H} NMR (126 MHz, C_6_D_6_): δ 44.10, 29.17, 26.61, 25.18. ^31^P­{^1^H} NMR (162 MHz, C_6_D_6_): δ 99.77. IR (ATR) ν [cm^–1^] = 3015 (m), 2946 (m), 2863 (m), 2147 (vw), 2129 (vw), 2042 (vw), 1971 (vw), 1587 (w), 1473 (m), 1379 (m), 1354 (w), 1303 (w), 1289 (ww),1127 (s), 1053 (s), 994 (s), 808 (s), 742 (w), 701 (w), 625 (s), 593 (s), 539 (s), 502 (w) 475 (s),413 (w). Elemental analysis, KCeC_48_H_104_N_8_P_4_, found (calculated): C 52.83 (52.57), H 9.80 (9.56), N 9.72 (10.22).

### [Ce^4+^NP^*t*^Bu_2_pyrr]_4_ (2-Ce­(NPC^2^))

Inside a glovebox, CeI_3_THF_4_ (145 mg, 179 μmol) was added to a 20 mL scintillation vial charged with a Teflon stir bar and 1 mL diethyl ether. KNPC^2^ (193 mg, 718 μmol) was added as a solid, and any residual ligand was transferred as a slurry in 1 mL diethyl ether. The reaction mixture was stirred overnight, then AgI (44 mg, 189 μmol) was added and stirred for 3 h. The reaction mixture was then filtered through a pipet filter packed with glass filter paper and Celite. The solvent was removed in vacuo, and the resulting pale-yellow oil was triturated with *n*-pentane (1 mL × 3). The waxy residue was taken up in 2 mL of *n*-pentane and concentrated in vacuo and placed in a −35 °C freezer overnight, during which time, bright yellow/orange XRD quality crystal grew to give the title compound (101 mg, 53%). ^1^H NMR (500 MHz, C_6_D_6_): δ 3.56 (t, 16H), 1.70 (t, 16H), 1.47 (d, 72H). ^13^C­{^1^H} NMR (126 MHz, C_6_D_6_): δ 49.39, 40.74, 40.21, 29.17, 26.56. ^31^P­{^1^H} NMR (202 MHz, C_6_D_6_): δ 7.58. IR (ATR) ν [cm^–1^] = 3016 (m), 2950 (m), 2864 (m), 2211 (vw), 2131 (vw), 2046 (vw), 1304 (w), 1088 (s), 998 (s), 809 (s), 633 (s), 602 (m), 556 (m), 507 (m), 474 (w), 411 (w). Elemental analysis, CeC_48_H_104_N_8_P_4_, found (calculated): C 54.33 (54.52), H 10.12 (9.91), N 10.55 (10.60).

### K­[Pr^3+^NP^*t*^Bu_2_pyrr]_4_ (1-KPr­(NPC^2^))

Inside a glovebox, PrI_3_THF_4_ (145 mg, 309 μmol) was added to a 20 mL scintillation vial charged with a Teflon stir bar and 1 mL diethyl ether. KNPC^2^ (192 mg, 716 μmol) was added as a solid, and any residual ligand was transferred as a slurry in 1 mL diethyl ether. The reaction mixture was stirred overnight and filtered through a pipet filter packed with glass filter paper and Celite. The solvent was concentrated in vacuo, and the resulting pale-yellow oil was triturated with *n*-pentane (1 mL × 3). The waxy residue was taken up in 2 mL of *n*-pentane and filtered through a pipet packed with Celite and glass fiber filter paper. The solution was concentrated in vacuo and then 5–10 drops of diethyl ether were added and the reaction mixture was placed in a −35 °C freezer overnight. During this time light-yellow XRD quality crystal grew to give the title compound (125 mg, 64%). ^1^H NMR (400 MHz, C_6_D_6_): δ −0.38 (t, 16H), −1.78 (d, 72H), −3.12 (t, 16H). ^13^C­{^1^H} NMR (126 MHz, C_6_D_6_): δ 48.04,38.48, 23.37, 23.27. ^31^P­{^1^H} NMR (162 MHz, C_6_D_6_): δ 274. IR (cm^–1^) = 3016 (m), 2209 (vw), 2164 (vw), 2134 (vw), 2030 (vw), 2017 (vw), 1975 (vw), 1304 (m), 577 (m), 503 (m), 438 (w), 421 (w), 410 (w). Elemental analysis, C_48_H_104_KN_8_P_4_Pr, found (calculated): C 50.88 (52.54), H 9.49(9.55), N 9.49 (10.21). Carbon is low on multiple analyses. University of Iowa elemental analysis facility cannot use a catalyst with air-sensitive samples, and the burn temperature is likely to low for complete combustion.

### Computational Details

All [Ln­(NPC^*x*^)]^*q*^ and [M^+^]­[Ln­(NPC^*x*^)]^q+1^ (M = K, Cs; *x* = 1, 2, 3; Ln = Ce and *q* = −1, 0; and Ln = Pr and *q* = −1, 0, 1) complexes were fully optimized in the gas phase without any constraints. Starting geometries were based on the XRD structures, when available. For Ln^4+^ and Pr^5+^ complexes which included an alkali metal counterion, the starting geometry was based on the optimized geometry of the Ln^3+^ complex with the intercalated alkali cation. The starting geometry for the Pr^3+^(NPC^1^) and Pr^4+^(NPC^1^) complexes were based on the optimized geometry for the Ce^3+^(NPC^1^) and Ce^4+^(NPC^1^) complexes while the starting geometry for the Pr^5+^(NPC^1^) complex was the optimized Pr^4+^(NPC^1^) complex. Geometry optimizations were carried out using the TPSSh^54,55^ DFT functional as implemented in the Gaussian16 software package (version A.03).^56^ The ECP28MWB^57^ small core quasi-relativistic pseudopotential and ECP28MWB_ANO^58,59^ basis set was used to describe Ce and Pr in all complexes. The ECP46MWB small core quasi-relativistic pseudopotential^60^ and ECP46MWB basis set^61^ was used to describe the Cs^+^ cation. All remaining atoms were described with the all-electron Pople basis set 6–311G­(d).^62^ This computational protocol has previously yielded excellent agreement with experimental observables for closely related NPC systems, including accurate reproduction of solid-state structures, redox potentials, and spectroscopic properties across lanthanide and actinide complexes, thereby supporting its reliability in describing the lanthanide NPC systems investigated in this work.^1,15,26,27,47,48^

G-parameters were calculated via the Solid-G program.^44^ In this methodology, the ligand solid angle $\left(\Omega =\frac{A}{r^{2}}\right)$ is used to calculate the percentage of a sphere of arbitrary radius shielded by a ligand, $G=100\frac{\Omega }{4\pi }$ , which provides a quantifiable definition for steric bulk. Specifically, G_M_(complex) describes the percentage of the metal M coordination sphere shielded by all ligands.

Natural population analysis (NPA) charges were calculated via the natural bond orbital version 7 (NBO7) code of Foster and Weinhold^63,64^ as implemented in Gaussian 16. The electron density at the N atom was calculated via the quantum theory of atoms in molecules (QTAIM)^65^ as implemented in Multiwfn.^66^ Molecular orbital diagrams were plotted using single point THF calculations from Gaussian and the program Chemissian v4.67.^67^

Redox potentials were calculated via the revised Born–Haber cycle^68^ with all redox potentials referenced to a calculated absolute half-cell potential of a ferrocene couple Fc^0/+^ in THF using the self-consistent reaction field approach based on the integral equation formalism of the polarized continuum model for implicit solvent.^69–71^ The TPSSh functional and LANL08 ^72,73^ basis set was used for Fe, while C/H atoms were described by the 6–311G­(d) basis set.

Metal-ion ejection thermodynamics were evaluated using single-point electronic energies computed in THF, combined with zero-point and thermal corrections obtained from gas-phase optimized geometries. The following chemical equation was used to calculate the adiabatic dissociation energy [Ln^q+^(NPC^*x*^)]­[M^+^] --> [Ln^q+^(NPC^*x*^)] + [M^+^].

## Supplementary Material



## Notes

Experimental considerations, NMR and UV–vis–NIR spectra, electrochemical, crystallographic data, computational methodology, G-parameter calculation, and natural population analysis (PDF). The optimized geometries (XYZ coordinates) for all computed structures have been deposited at the figshare repository and are accessible (via DOI: 10.6084/m9.figshare.30338485 PDF)
