# Supplementary material for: Ligand Design Criteria for the Stability of High Oxidation State Praseodymium Complexes
Source: Inorg Chem. 2026 Jan 13;65(3):1991–2001. doi: 10.1021/acs.inorgchem.5c05024 (PMC12848976; doi:10.1021/acs.inorgchem.5c05024)
Supplement: Supplementary file 1 [file ic5c05024_si_001.pdf]

Supporting Information for

Ligand Design Criteria for the Stability of High Oxidation State Praseodymium Complexes

Tyler-Rayne Nero,[†] Chad M. Studvick,[§] Andrew C. Boggiano,[†] Maximilian G. Bernbeck,[†] Ivan A. Popov,[*#] and Henry S. La Pierre[*†‡]

[†]School of Chemistry and Biochemistry, Georgia Institute of Technology, Atlanta, Georgia 30332-0400, United States

[‡]Nuclear and Radiological Engineering and Medical Physics Program in the School of Mechanical Engineering, Georgia Institute of Technology, Atlanta, Georgia 30332-0400, United States

[§]Department of Chemistry, The University of Akron, Akron, Ohio 44325-3601, United States

[#]Washington State University, Department of Chemistry, Pullman, Washington 99164-4630, United States

*Email: hsl@gatech.edu; ivan.popov@wsu.edu

Table of Contents

General Considerations **2**

Synthetic Methods **3**

NMR Spectroscopy **4**

Electrochemistry **14**

UV-vis-NIR Spectra **18**

Single-Crystal X-ray Diffraction (SC-XRD) **19**

Computational Details **22**

References **40**

General Considerations

Unless otherwise noted, all reagents were obtained from commercial suppliers and all syntheses and manipulations were conducted with the exclusion of oxygen and water using Schlenk techniques under Ar or in a N_2 filled glovebox (Vigor, <0.1 ppm O_2/H_2O). The glovebox is equipped with two -35 °C freezers and a cold well. All glassware and cannulas/needles were stored in an oven overnight (>8h) at a temperature of ca. 160 °C before use.

Materials: All reagents were obtained from commercial suppliers and used as received except as described below. Celite and molecular sieves were heated under vacuum at a temperature >250 °C for a minimum of 24 h. C_6D_6 (Cambridge Isotope Laboratories or Sigma-Aldrich) was stored over 3 Å molecular sieves and then vacuum transferred from purple sodium/benzophenone and stored over fresh 3 Å molecular sieves before use. THF-d_8 (Cambridge) was degassed by three freeze-pump-thaw cycles and vacuum transferred from purple sodium/benzophenone, then stored over fresh 3 Å molecular sieves prior to use. Diethyl ether, n-pentane, hexane, toluene, and tetrahydrofuran (THF) were purged with UHP-grade argon (Airgas) and passed through columns containing Q-5/alumina and molecular sieves in a solvent purification system (JC Meyer Solvent Systems, Pure Process Technology), then stored over 10% v/v 3 Å molecular sieves in media bottles inside the glovebox. Methanol was dried by refluxing over magnesium turnings activated with iodine overnight under argon and then distilled and stored over 3 Å molecular sieves. Potassium *tert*-butoxide was sublimed before use in the synthesis of benzyl potassium. Graphite was flame dried in a Schlenk flask under vacuum on a Schlenk line several times before use in preparing KC_8. KC_8 was prepared by melting a stoichiometric (1:8 K:C) amount of potassium over graphite inside the glovebox. Silver iodide (AgI, Strem) was ground to a fine powder using a mortar and pestle in the absence of light before use. Cargile Type-NVH immersion oil was degassed on a Schlenk line by stirring under an active vacuum with gentle heating (to facilitate stirring, ca. 40 °C) overnight before use.

Nuclear Magnetic Resonance (NMR) Spectroscopy: NMR spectra were obtained on a Bruker Avance III 400 or 500 MHz spectrometer at 298 K unless otherwise noted. ^{1}H, ^{13}C{^{1}H}, and ^{31}P{^{1}H} NMR chemical shifts are reported in δ, parts per million. ^{1}H NMR are referenced to the residual ^{1}H resonances of the solvent. ^{13}C{^{1}H} NMR spectra are referenced to the resonance of the deuterated solvent. ^{31}P{^{1}H} spectra are externally referenced to H_3PO_4. Peak position is listed, followed by peak multiplicity, integration value, and proton assignment, where applicable. Multiplicity and shape are indicated by one or more of the following abbreviations: s (singlet); d (doublet); t (triplet); q (quartet); dd (doublet of doublets); td (triplet of doublets); m (multiplet); br (broad).

Optical Spectroscopy: UV-visible-NIR spectroscopy was performed in small-volume screw-cap quartz cuvettes (Starna Scientific) with a 1 cm path length on a Hitachi UH4150 UV-vis-NIR scanning spectrophotometer. ATR infrared measurements were performed on powder samples using a Bruker ALPHA FTIR spectrometer from 400 to 4000 cm^{-1} inside a dinitrogen glovebox.

Cyclic Voltammetry (CV): Electrochemical data were measured using a Pine WaveDriver 20 Bipotentiostat/Galvanostat. Measurements were performed in a glovebox under an atmosphere of N_2 with a glassy carbon working electrode (3 mm diameter), a bare silver wire reference electrode in a fritted capillary filled with the corresponding electrolyte solution, and a platinum wire counter electrode at ambient temperature (~25 °C). The fritted capillary was stored in an electrolyte solution when not in use, and the inner solution was replaced with a fresh electrolyte solution before use. The glassy carbon and silver wire electrodes were polished before use. Electrolyte solutions were prepared in THF. Measurements were made in positive feedback iR compensation mode (~500 Ω for 0.1 M $[^nBu_4N][BPh_4]$ in THF). Voltammograms were externally referenced by adding a small amount of decamethylferrocene at the end of each experiment. All potentials are reported *vs*. Fc/Fc^+, using the conversion of decamethylferrocene E°' = -0.5 V *vs*. Fc/Fc^+ for measurements using $[^nBu_4N][BPh_4]$, in THF.[1]

Combustion Analysis: Elemental analysis data (C, H, N) were collected at the University of California Berkeley Microanalytical Facility (Berkeley, CA) and the Exeter Analytical CE-440 elemental analyzer at the University of Iowa MATFab Facility.

Single-Crystal X-ray Diffraction (SC-XRD): X-ray structural determinations were performed at the Georgia Institute of Technology X-ray Crystallography Facility on a Bruker D8 Venture diffractometer. Crystals for X-ray analysis were coated in Cargille Type-NVH immersion oil inside a glovebox and brought to the diffractometer in a capped 20 mL scintillation vial.

Further Synthetic Methods

Chemical Oxidation of 1-KPr(NPC^2). Inside a glovebox, **1-KPr(NPC^2)** (19.2 mg, 17.5 μmol) was dissolved in 0.7 mL THF-d_8 in a 20 mL scintillation vial. The solution was transferred to a 4 mL vial, containing $FcBArF_{20}$ (30 mg, 34.7 μmol) and agitated by hand for 10 min. The reaction mixture was transferred to the NMR tube and brought to the NMR facility.

^{1}H NMR (400 MHz, THF-d_8) δ 4.12 (s, 72H) 3.16 (s, 16H), 1.04 (s, 16H), -0.29 (s, 25H), -0.84 (s, 5H), -2.37 (s, 5H). ^{31}P{^{1}H} NMR (162 MHz, THF-d_8) δ 268 and 65. ^{19}F{^{1}H} (376 MHz, THF-d_8) -131.23, -163.37, -167.15.

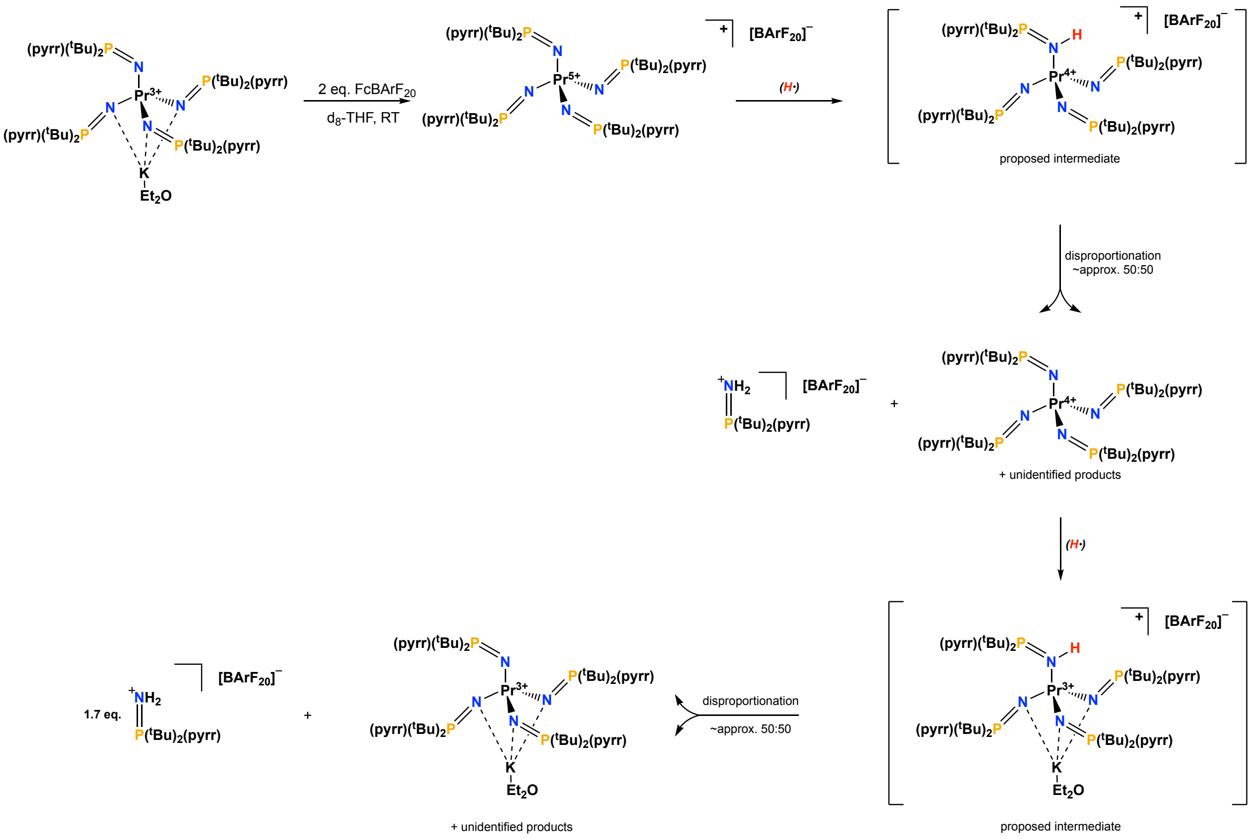

Scheme S1. Proposed disproportionation pathway of **1-KPr(NPC²)**.

NMR Spectroscopy

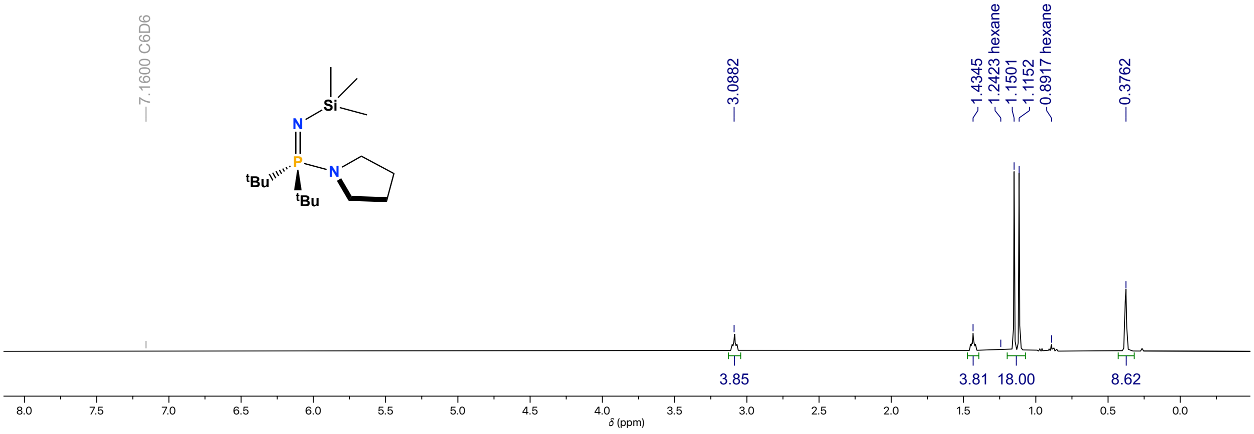

Figure S1. ¹H NMR of **TMSNPC²** intermediate in C_6D_6. Residual solvent is annotated in the spectrum.

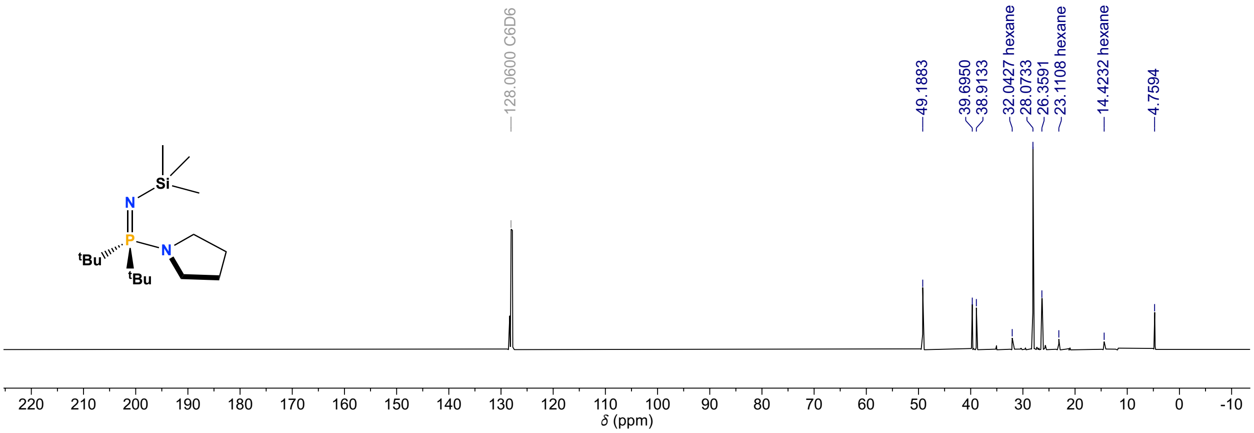

Figure S2. $^{13}C\{^{1}H\}$ NMR of **TMSNPC²** intermediate in C_6D_6. Residual solvent is annotated in the spectrum.

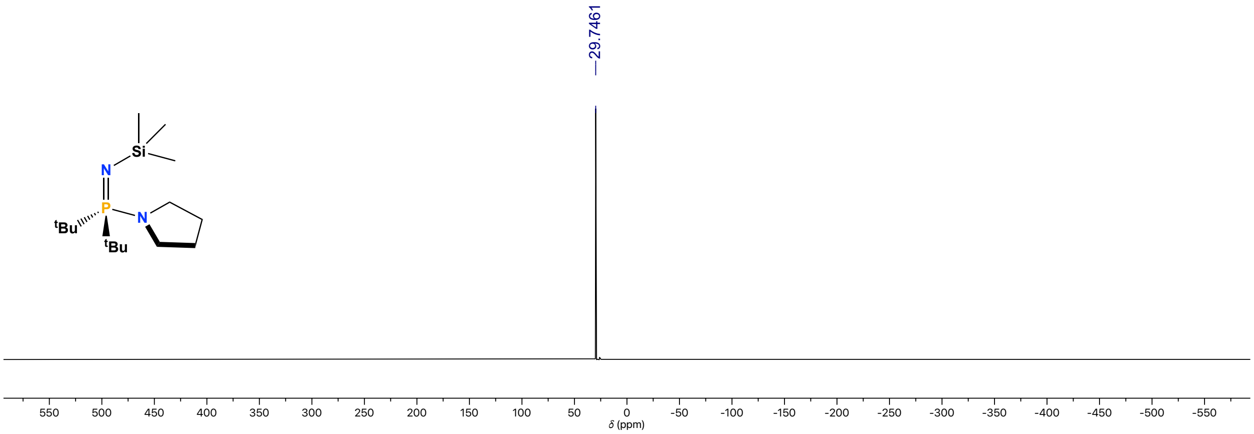

Figure S3. $^{31}P\{^{1}H\}$ NMR of **TMSNPC²** intermediate in C_6D_6.

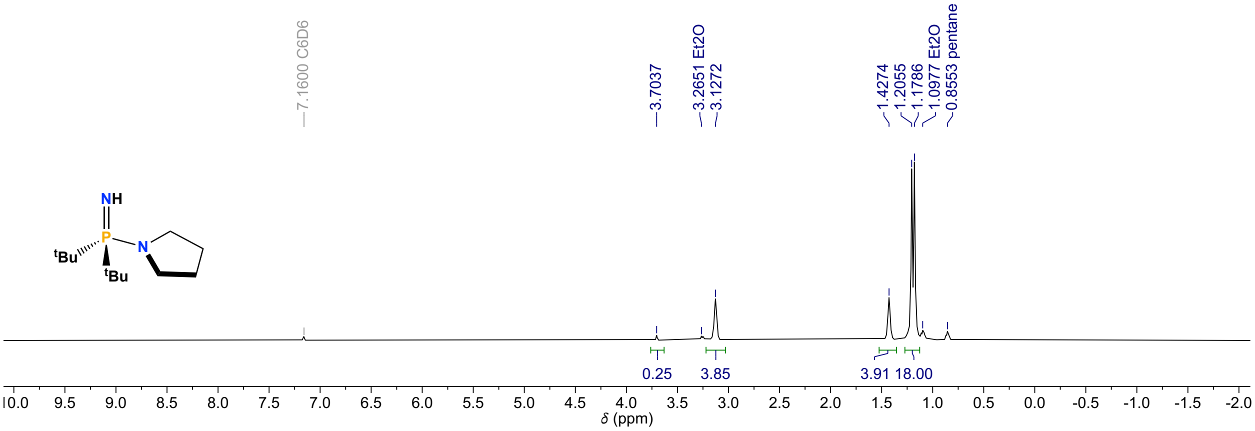

Figure S4. ^{1}H NMR of **HNPC²** in C_6D_6. Residual solvent is annotated in the spectrum.

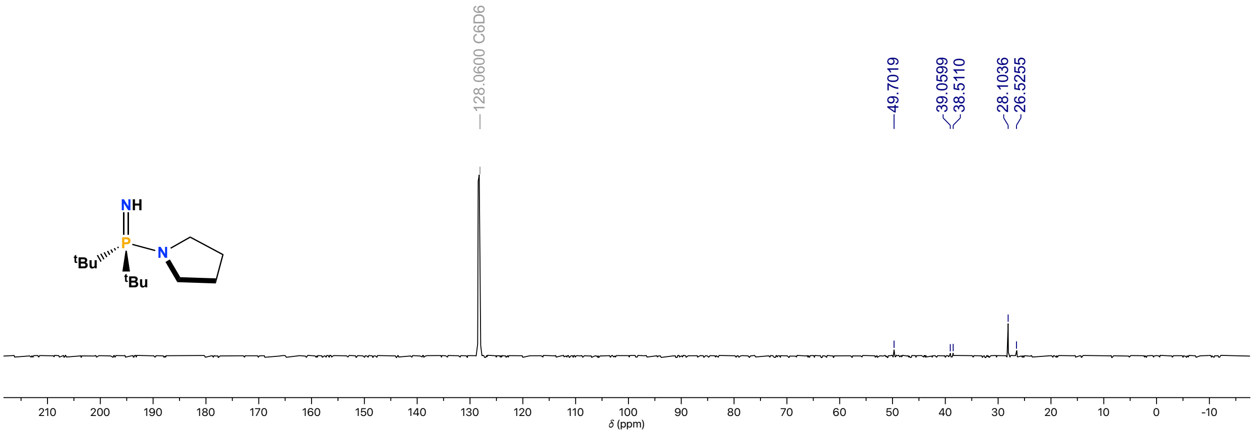

Figure S5. ${}^{13}C\{{}^{1}H\}$ NMR of **HNPC²** in C_6D_6.

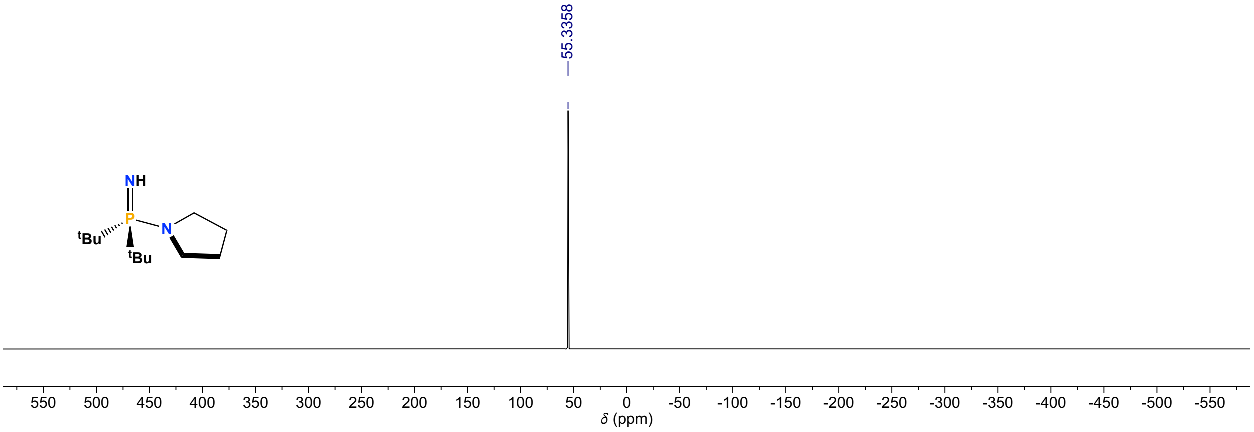

Figure S6. ${}^{31}P\{{}^{1}H\}$ NMR of **HNPC²** in C_6D_6.

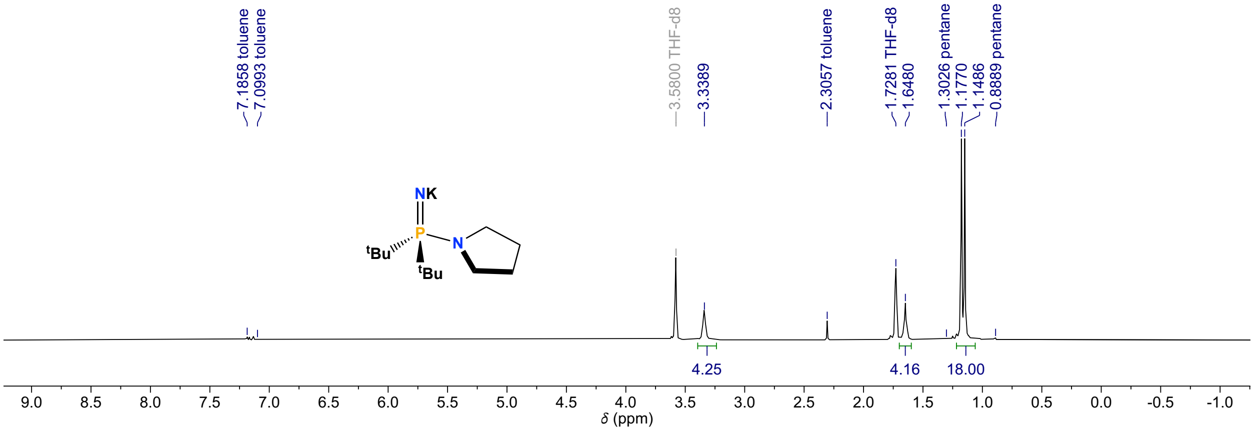

Figure S7. ${}^{1}H$ NMR of **KNPC²** in THF-d_8. Residual solvent is annotated in the spectrum.

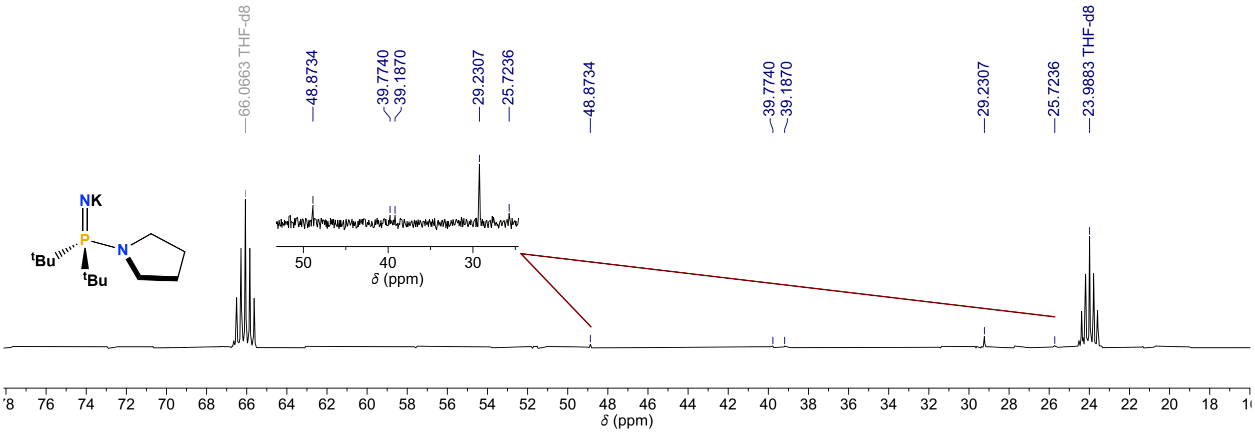

Figure S8. $^{13}C\{^{1}H\}$ NMR of **KNPC²** in THF-d_8.

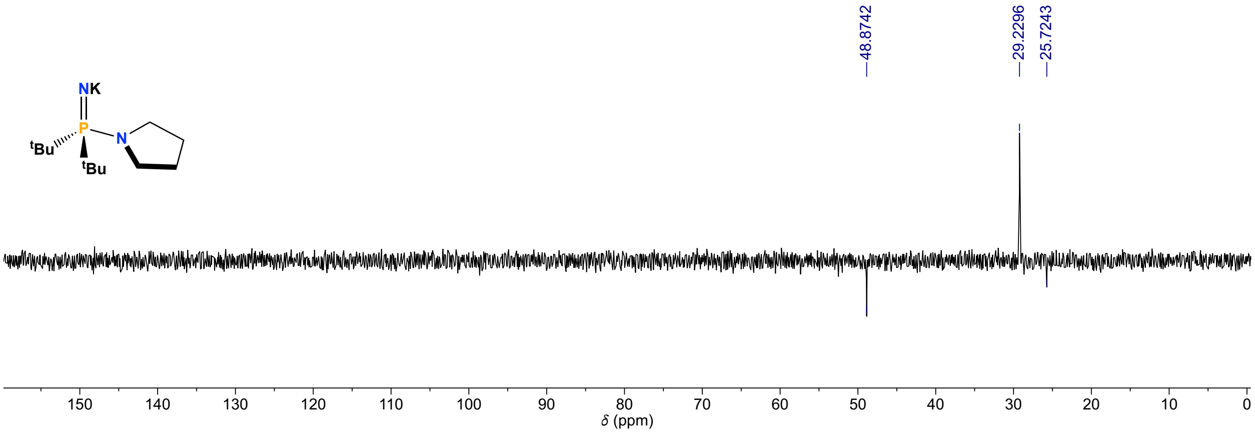

Figure S9. Dept-135 NMR spectrum of **KNPC²** in THF-d_8.

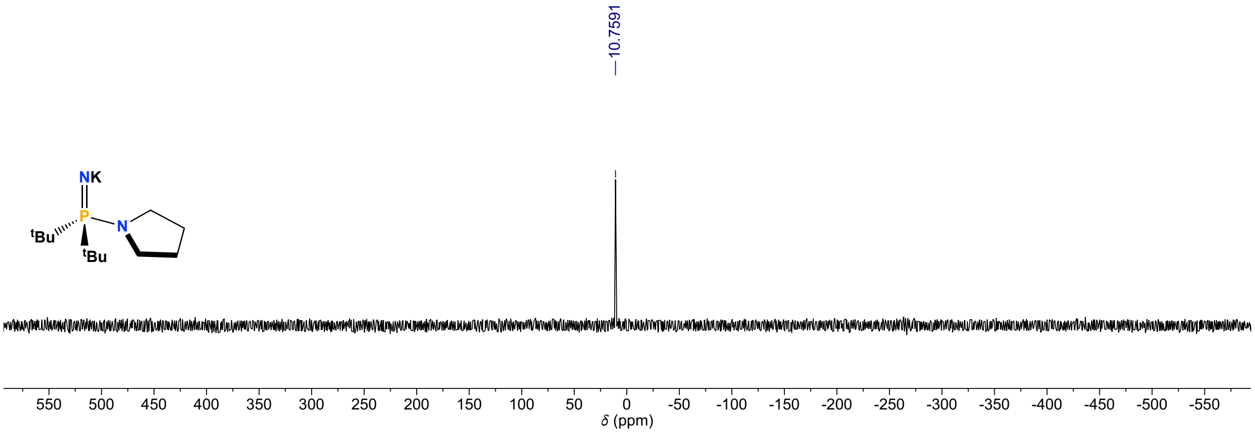

Figure S10. $^{31}P\{^{1}H\}$ NMR of **KNPC²** in THF-d_8.

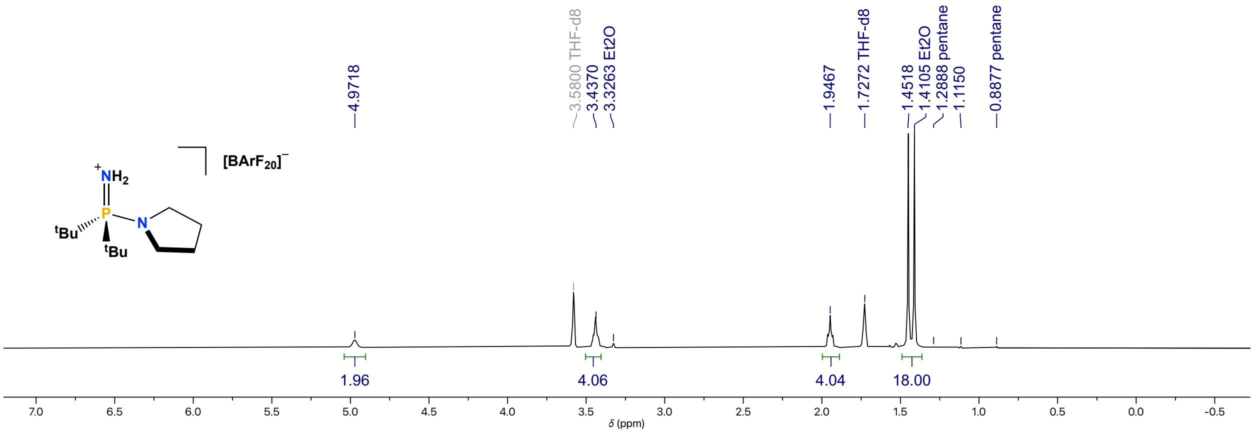

Figure S11. ^{1}H NMR of **[H$_2$NPC^2][BArF$_{20}$]** in THF-d_8. Residual solvent is annotated in the spectrum.

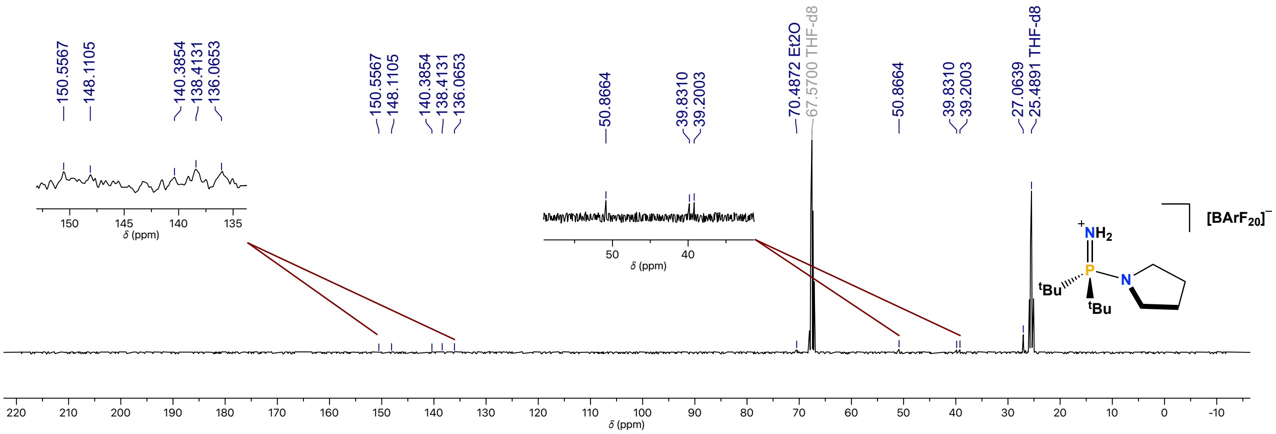

Figure S12. $^{13}C\{^{1}H\}$ NMR of **[H$_2$NPC^2][BArF$_{20}$]** in THF-d_8. Residual solvent is annotated in the spectrum.

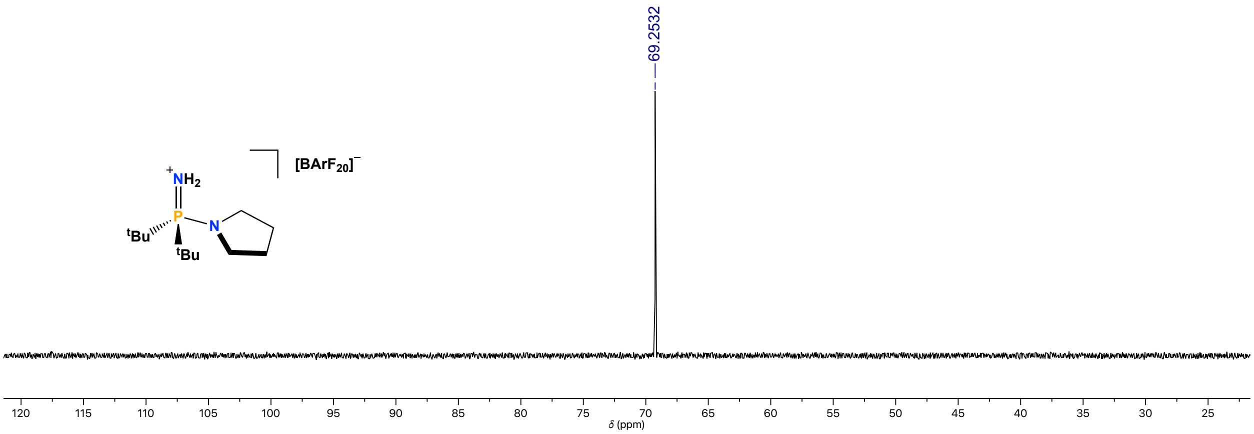

Figure S13. $^{31}P\{^{1}H\}$ NMR of **[H$_2$NPC^2][BArF$_{20}$]** in THF-d_8.

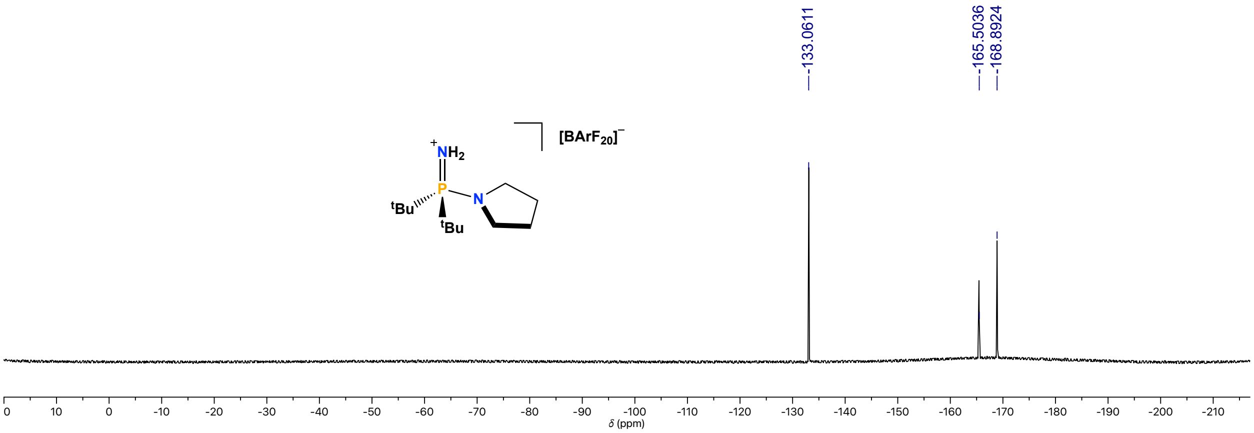

Figure S14. $^{19}F\{^{1}H\}$ NMR of **[H_2NPC^2][BArF$_{20}$]** in THF-d_8.

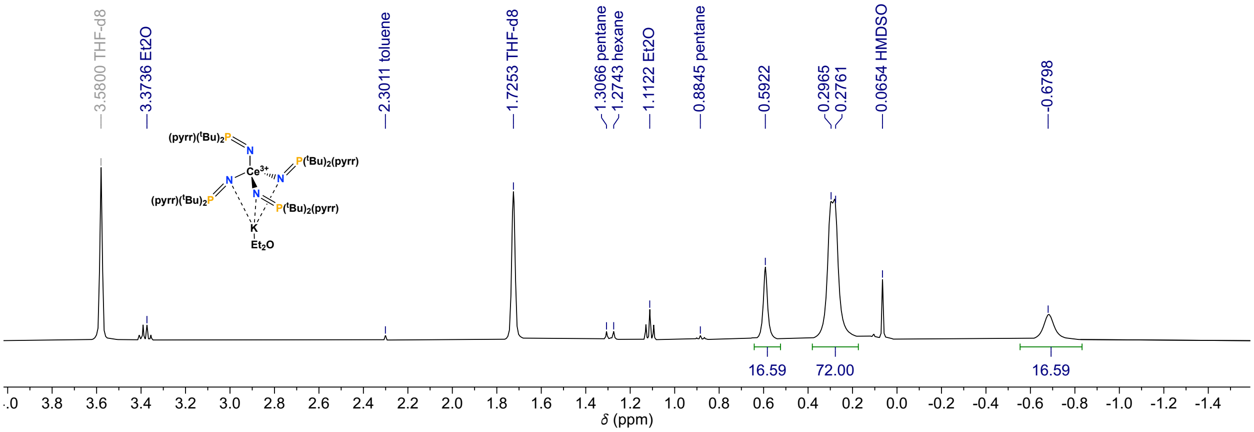

Figure S15. ^{1}H NMR of **1-KCe(NPC^2)** in THF-d_8. Residual solvent is annotated in the spectrum.

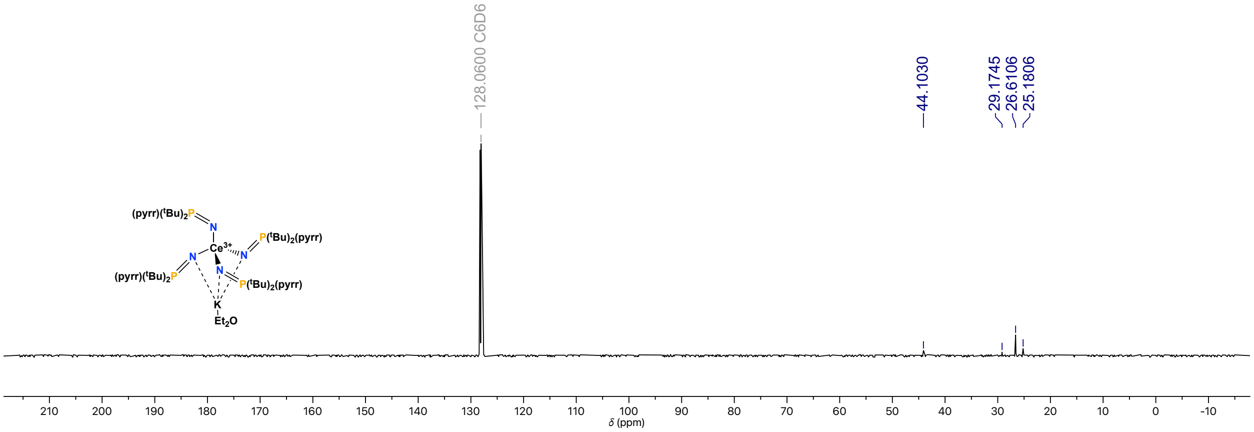

Figure S16. $^{13}C\{^{1}H\}$ NMR of **1-KCe(NPC^2)** in C_6D_6.

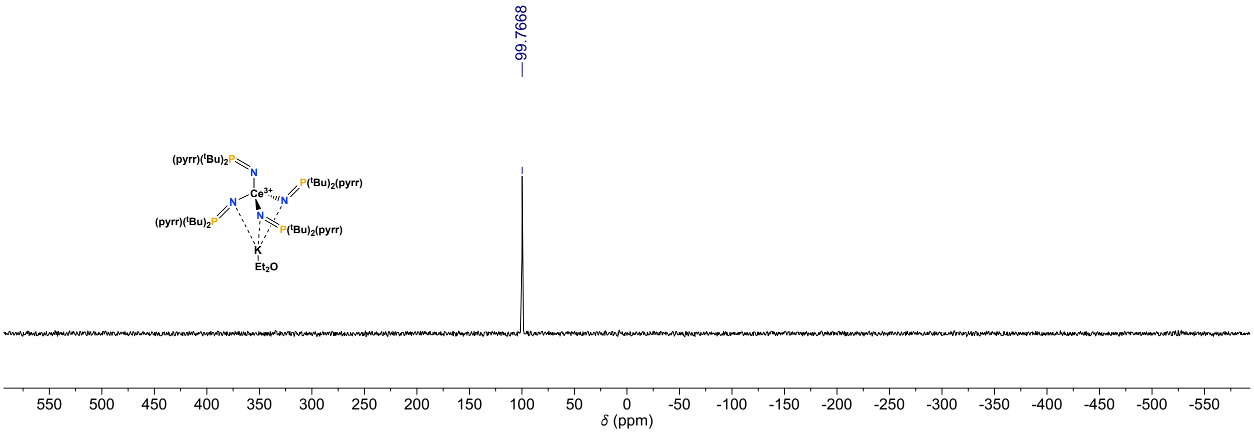

Figure S17. $^{31}P\{^{1}H\}$ NMR of **1-KCe(NPC^{2})** in C_6D_6.

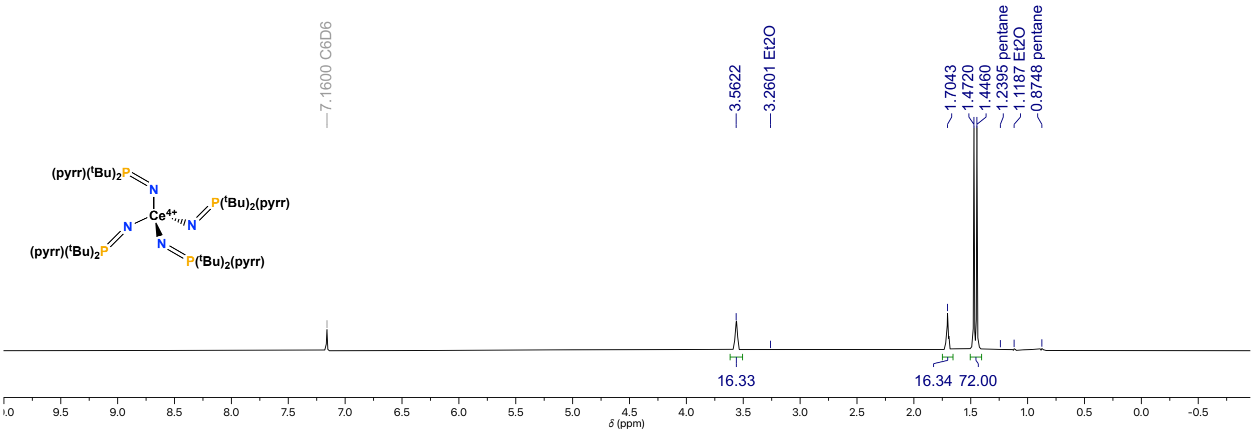

Figure S18. ^{1}H NMR of **2-Ce(NPC^{2})** in C_6D_6. Residual solvent is annotated in the spectrum.

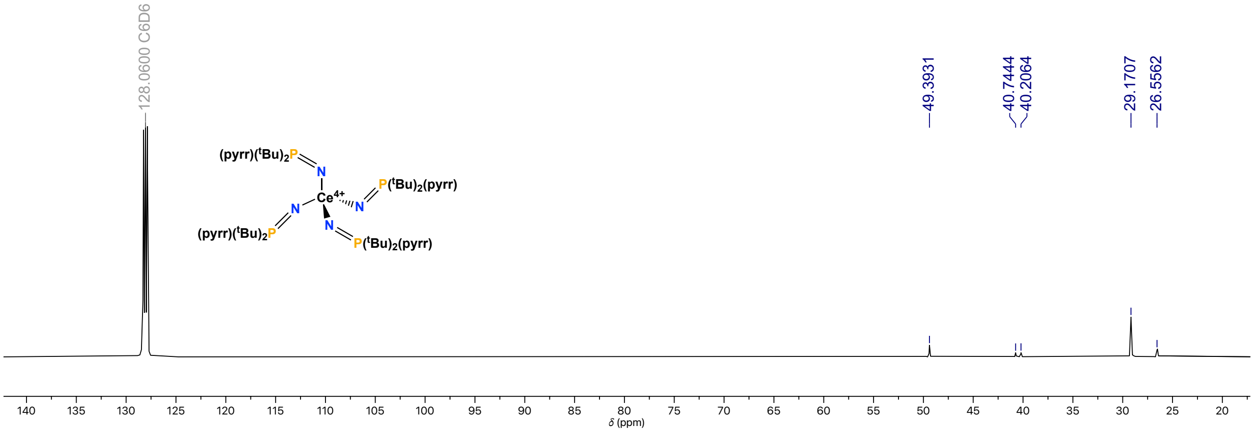

Figure S19. $^{13}C\{^{1}H\}$ NMR of **2-Ce(NPC^{2})** in C_6D_6.

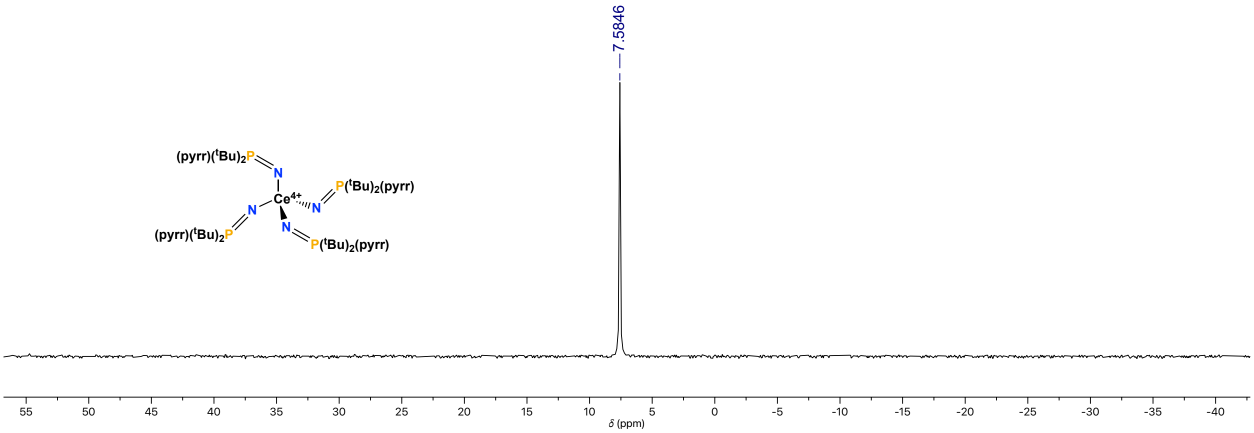

Figure S20. $^{31}P\{^1H\}$ NMR of **2-Ce(NPC²)** in C_6D_6.

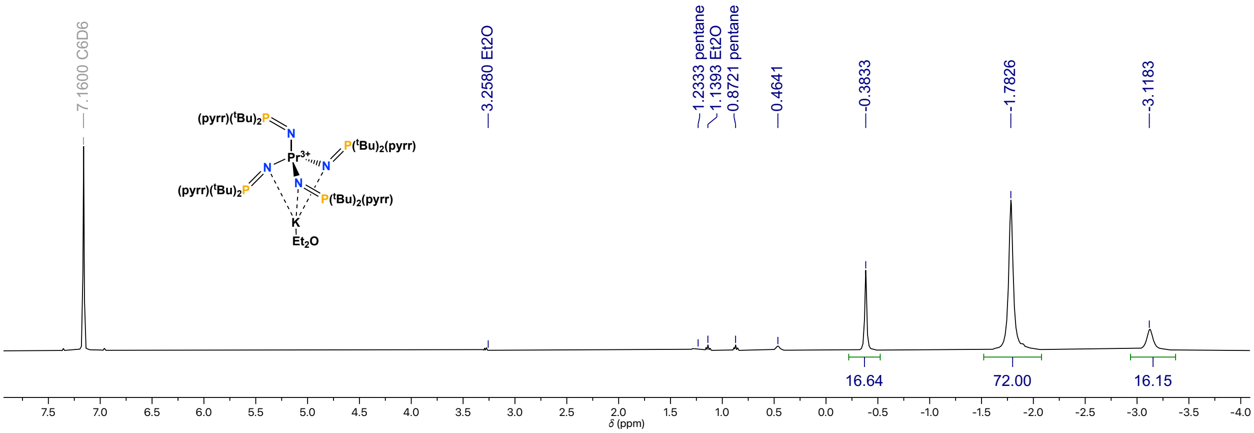

Figure S21. ^1H NMR of **1-KPr(NPC²)** in C_6D_6. Residual solvent is annotated in the spectrum.

PR3+(NPC2)_EA.13.fid

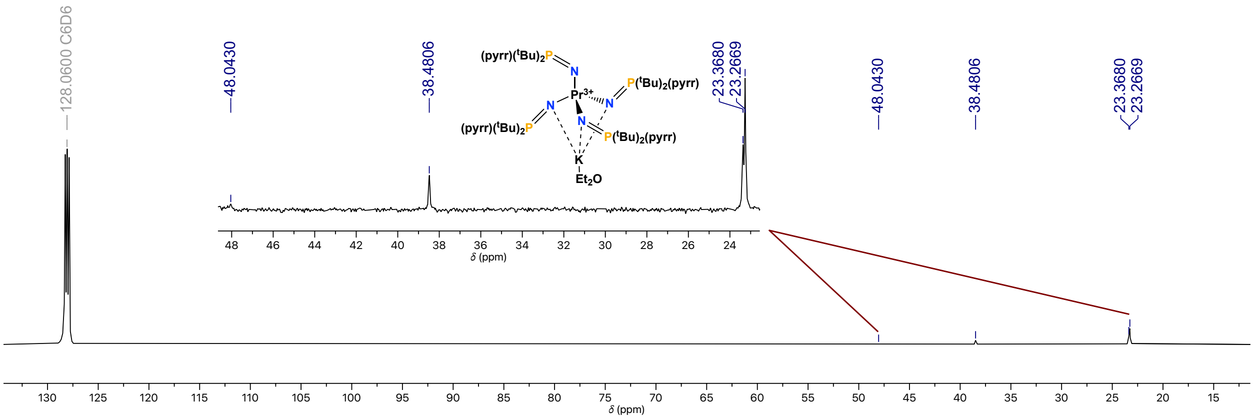

Figure S22. $^{13}C\{^1H\}$ NMR of **1-KPr(NPC²)** in C_6D_6.

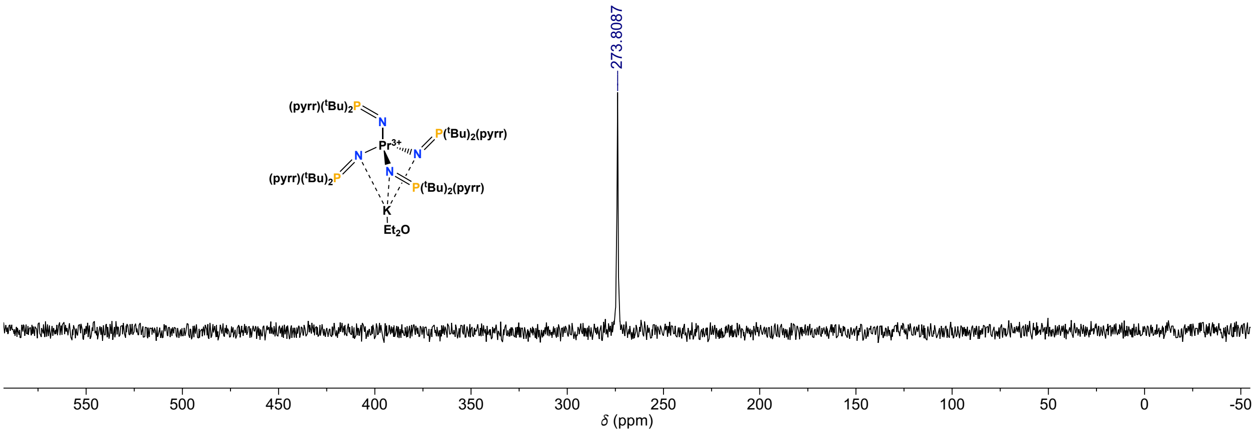

Figure S23. $^{31}P\{^1H\}$ NMR of **1-KPr(NPC²)** in C_6D_6.

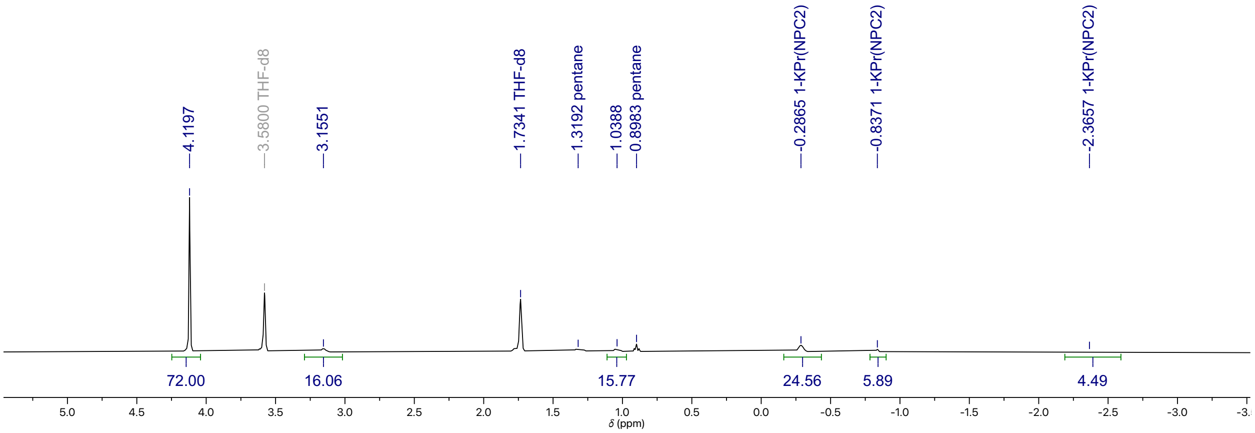

Figure S24. ^1H NMR of the reaction between **1-KPr(NPC²)** and 2 equiv. [Fc][BArF$_{20}$] in THF-d_8.

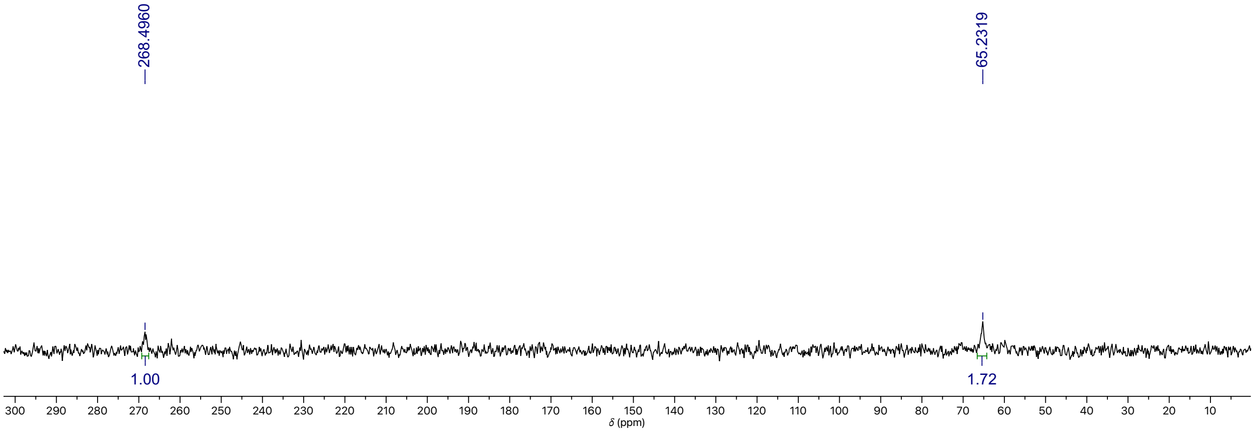

Figure S25. $^{31}P\{^1H\}$ NMR of the reaction between **1-KPr(NPC²)** and [Fc][BArF$_{20}$] in THF-d_8.

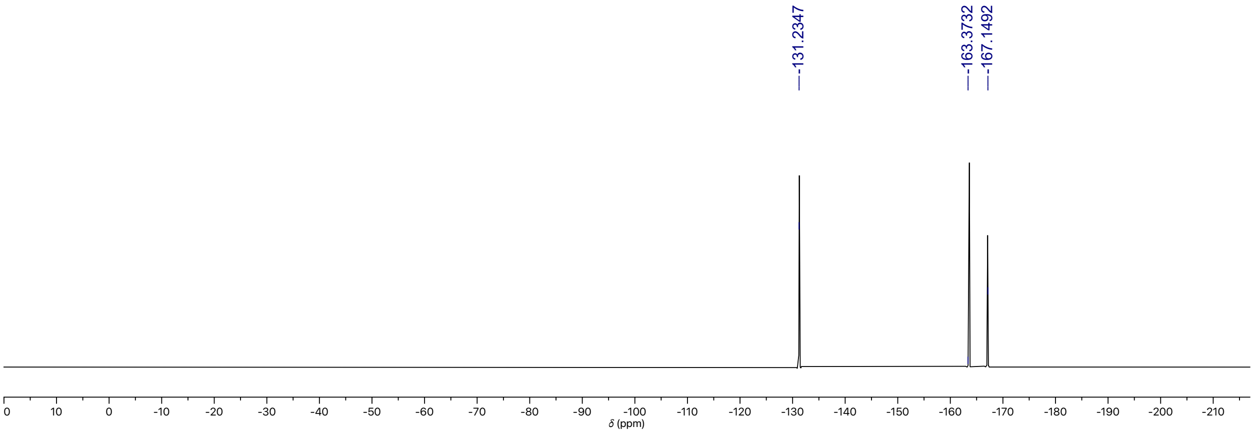

Figure S26. $^{19}F\{^{1}H\}$ NMR of the reaction between **1-KPr(NPC²)** and [Fc][BArF₂₀] in THF-d_8.

Electrochemistry

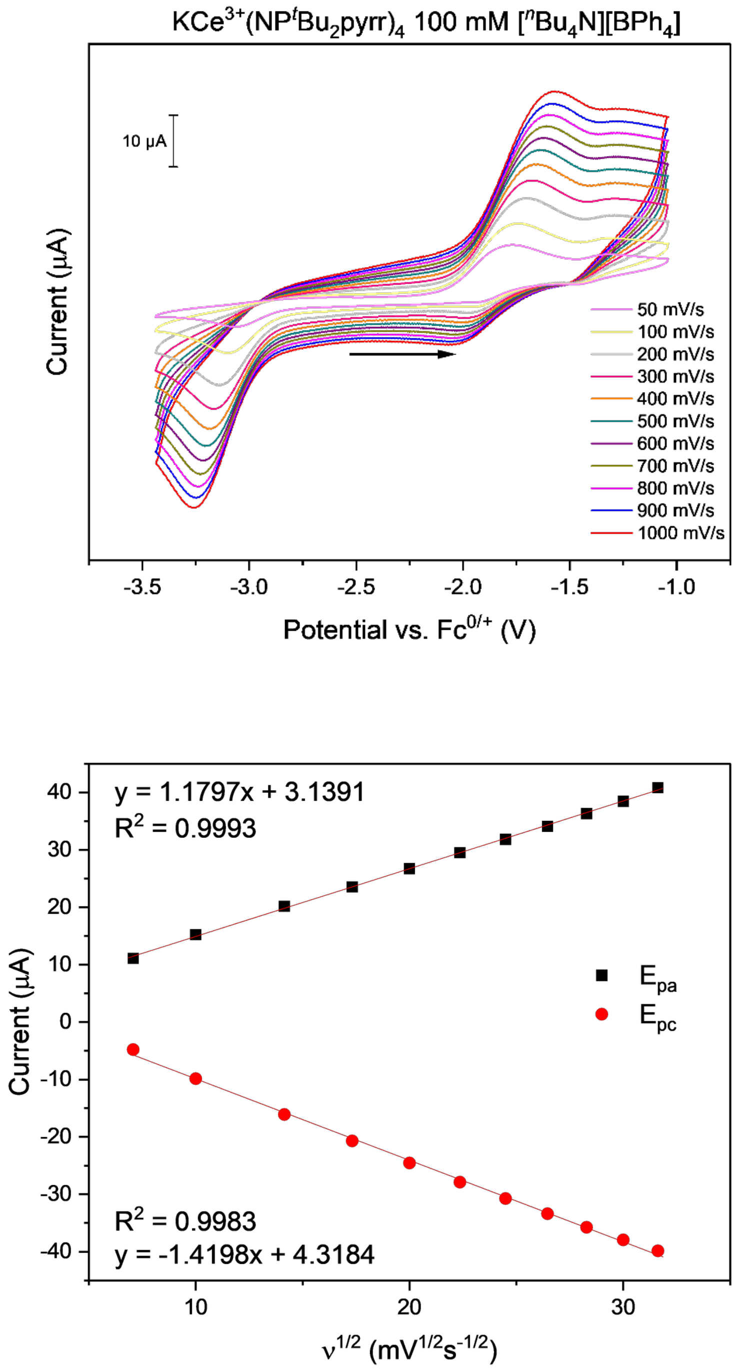

Figure S27. Scan-rate dependence of cyclic voltammogram of 3 mM **1-KCe(NPC²)** in 100 mM $[^nBu_4N][BPh_4]$ in hexane (top) and Randles-Sevcik plot (bottom).

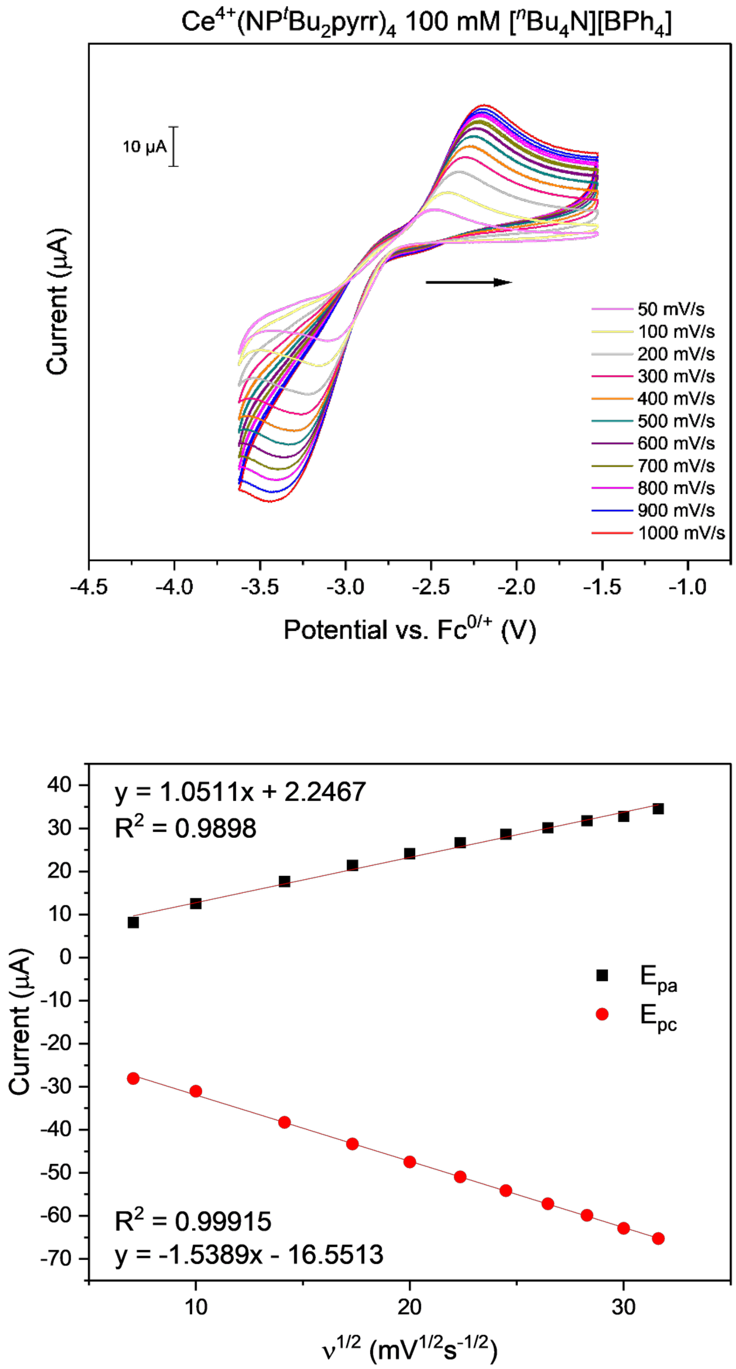

Figure S28. Scan-rate dependence of cyclic voltammogram of 3 mM **2-Ce(NPC²)** in 100 mM [^{n}Bu$_4$N][BPh$_4$] in THF (top) and Randles-Sevcik plot (bottom).

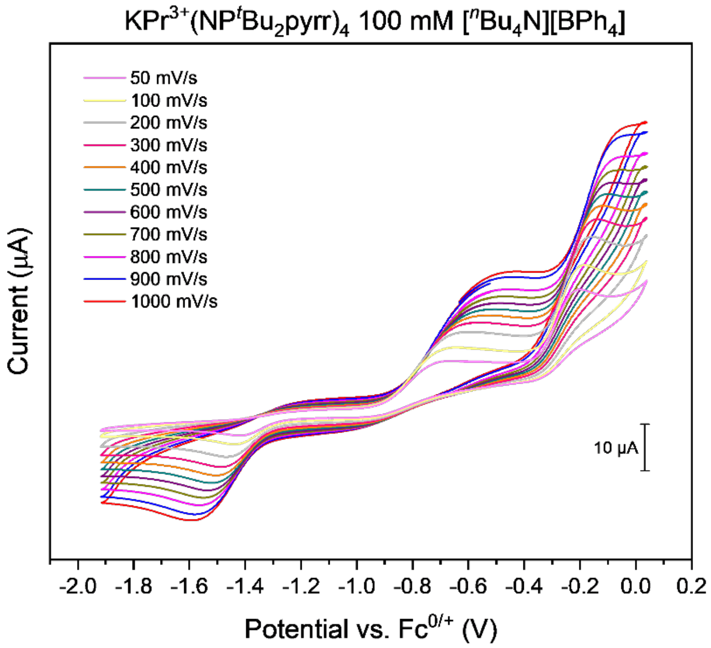

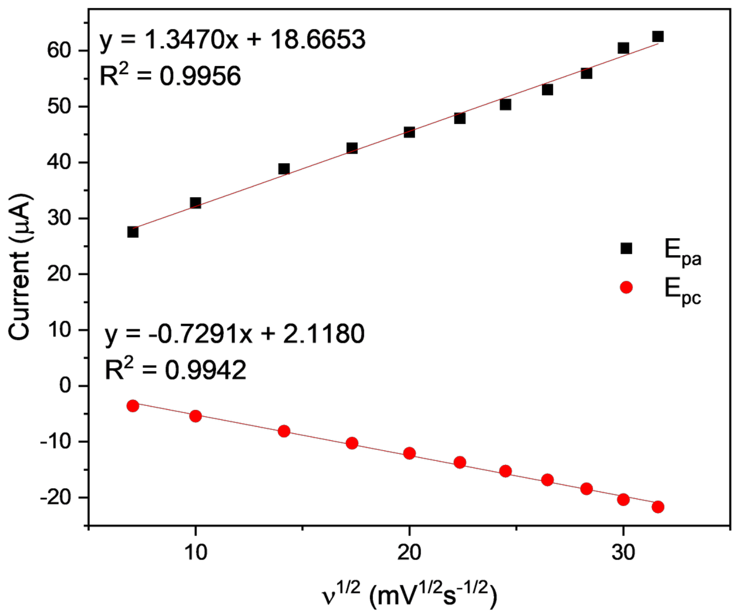

Figure S29. Scan-rate dependence of cyclic voltammogram of 3 mM **1-KPr(NPC²)** in 100 mM [^{n}Bu$_4$N][BPh$_4$] in THF (top) and Randles-Sevcik plot (bottom).

Table S1. Electrochemical potentials (V *vs*. $Fc^{0/+}$) for 1-Ln(NPC^x), 1-Ln(NP^*), 2-Ln(NPC^x), and 2-Ln(NP^*) (x = 1, 2, 3) at 200 mV/s.[2-4]

	E_{pa1} (V)	E_{pc1} (V)	$E_{1/2}$ (V)	E_{pa2} (V)	E_{pc2} (V)	$E_{1/2}$ (V)
**1-KPr(NPC^2)**	-0.60	-1.47	-	-0.16	-0.33	-
**1-CsPr(NPC^3)**	-1.26	-1.45	-1.35	-0.24	-0.35	-0.29
**1-KPr(NP^*)**	-0.72	-1.48	-	-	-	-
**1-KCe(NPC^2)**	-1.69	-3.14	-	-	-	-
**1-CsCe(NPC^3)**	-2.26	-3.01	-	-	-	-
**1-KCe(NP^*)**	-1.44	-2.88	-	-	-	-
**2-Ce(NPC^1)**	-2.28	-2.91	-2.60	-	-	-
**2-Ce(NPC^2)**	-2.33	-3.22	-	-	-	-
**2-Ce(NPC^3)**	-2.33	-3.01	-	-	-	-
**2-Ce(NP^*)**	-1.63	-2.86	-	-	-	-

UV-vis-NIR Spectra

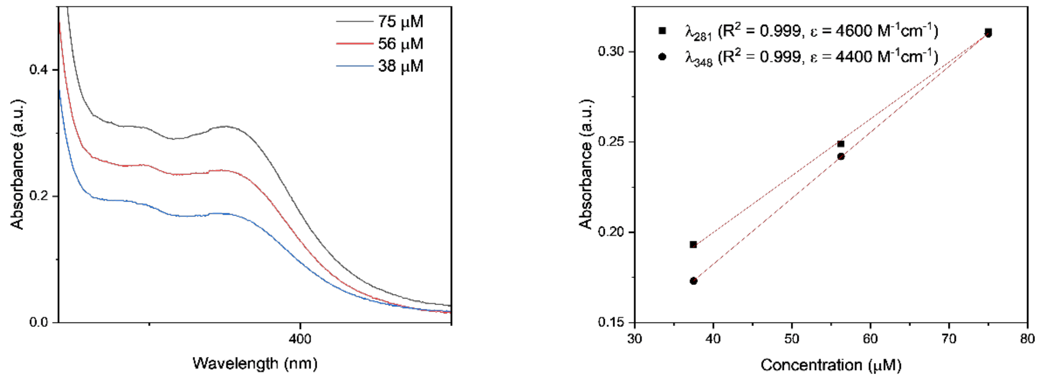

Figure S30. UV-vis-NIR spectrum of **1-KCe(NPC²)** in THF at multiple concentrations (left) and linear regression analysis to determine molar absorptivity coefficients (right).

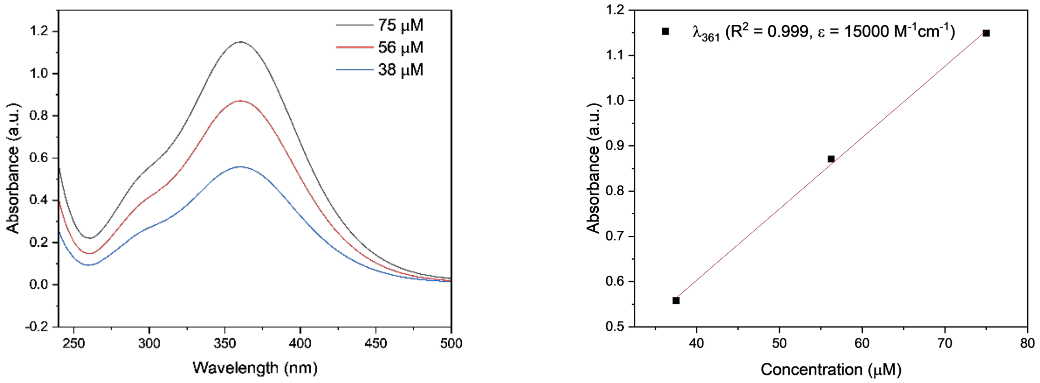

Figure S31. UV-vis-NIR spectrum of **2-Ce(NPC²)** in THF at multiple concentrations (left) and linear regression analysis to determine molar absorptivity coefficients (right).

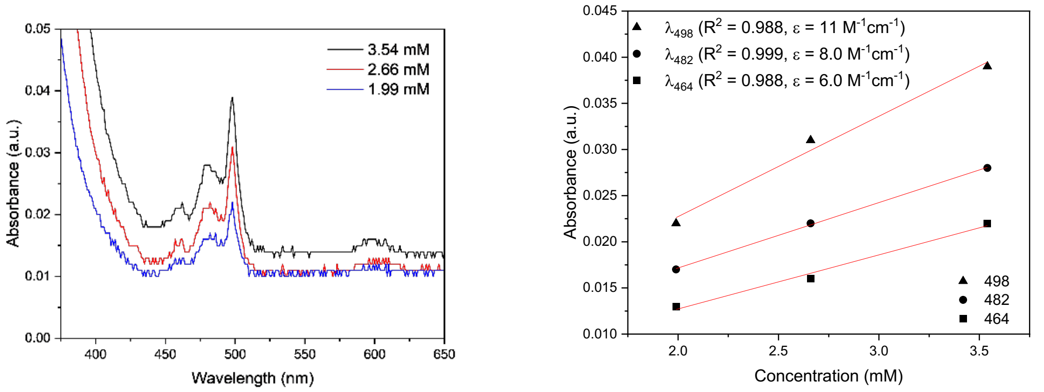

Figure S32. UV-vis-NIR spectrum of **1-KPr(NPC²)** in THF at multiple concentrations (left) and linear regression analysis to determine molar absorptivity coefficients (right). This spectrum

is at the solubility limit of the compound, exceeding both the cuvette and instrument detection limits in the UV–vis measurement.

Single-Crystal X-ray Diffraction (SC-XRD)

Table S2. Crystallographic parameters and metrics.

	1-KPr(NPC^2)	1-KCe(NPC^2)	2-Ce(NPC^2)	KNPC^2
Formula	$C_{50}H_{109}KN_8O_{0.50}P_4Pr$	$C_{52}H_{114}CeKN_8OP_4$	$C_{48}H_{104}CeN_8P_4$	$C_{55}H_{112}KN_4P_4$
Formula Weight/ gmol^{-1}	1134.34	1170.61	1057.39	1165.80
Collection Temperature/K	100.00	100.00	100.00	100.00
Space Group	$P2_1/n$	$Pca2_1$	$P\overline{1}$ (2)	$Pa\overline{3}$
System	monoclinic	orthorhombic	triclinic	Cubic
Resolution/Å	0.80	0.80	0.70	0.70
a/Å	14.1100(8)	26.4037(12)	12.5986(4)	23.4982(6)
b/Å	20.3149(12)	14.4212(6)	12.6119(4)	23.4982(6)
c/Å	21.9281(11)	33.0187(13)	40.6393(13)	23.4982(6)
α/°	90	90	94.724(2)	90
β/°	93.323(2)	90	95.049(2)	90
γ/°	90	90	116.6700(10)	90
Volume/Å^3	6275.0(6)	12572.6(9)	5693.8(3)	12974.9(10)
Z[Z']	4[1]	8[2]	4[2]	8[0.3]
ρ/gcm^{-3}	1.201	1.237	1.234	1.194
μ/mm^{-1}	0.982	0.932	0.949	0.413
F(000)	2428	5016	2264	5072
Crystal Size/mm^3	0.687×0.368×0.314	0.588×0.522×0.144	0.296×0.166×0.154	0.356x0.216x0.203
Radiation Type	MoK_α (λ=0.71073 Å)	MoK_α (λ=0.71073 Å)	MoK_α (λ=0.71073 Å)	MoK_α (λ=0.71073 Å)
Physical Description	Clear colorless block	clear yellow plank	clear yellow block	Clear colorless block
Collection 2θ range/°	3.52 to 52.74	3.32 to 52.74	3.65 to 61.02	3.88 to 60.99
Index Ranges	$-18 \le h \le 18$ $0 \le k \le 26$ $0 \le l \le 28$	$0 \le h \le 33$ $0 \le k \le 18$ $-41 \le l \le 0$	$-17 \le h \le 17$ $-18 \le k \le 18$ $-58 \le l \le 58$	$-33 \le h \le 33$ $-33 \le k \le 33$ $-33 \le l \le 33$
Reflections Collected	12822	13028/12613	331649	404790
Independent Reflections	12822 R_{int} = 0.1350 R_{sigma} = 0.0302	13028 R_{int} = 0.1195 R_{sigma} = 0.0310	34695 R_{int} = 0.0848 R_{sigma} = 0.0394	6620 R_{int} = 0.1474 R_{sigma} = 0.0284
Data/Restraints/Parameters	12822/1272/1168	13028/294/1458	34695/1833/2015	6620/312/362
Goodness of Fit	1.156	1.139	1.169	1.104
Final R Indices (I ≥ 2σ)	R_1 = 0.0644 wR_2 = 0.1637	R_1 = 0.0346 wR_2 = 0.0716	R_1 = 0.0428 wR_2 = 0.0959	R_1 = 0.0670 wR_2 = 0.1376
Final R Indices (all data)	R_1 = 0.0703 wR_2 = 0.1677	R_1 = 0.0370 wR_2 = 0.0723	R_1 = 0.0478 wR_2 = 0.0982	R_1 = 0.0854 wR_2 = 0.1470
Largest Diff. Peak/Hole/eA^3	1.23/-1.50	0.44/-0.61	1.00/-1.28	0.75/-0.93
Completeness to 2θ/%	99.8	99.9	100.0	99.9
CCDC Number	2479614	2479616	2479615	2487617

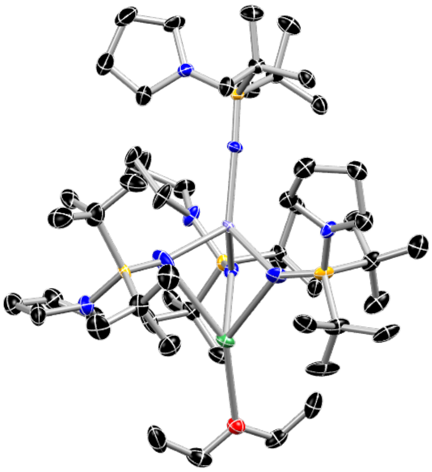

Figure S33. Molecular structure of **1-KCe(NPC²)**. Thermal ellipsoids are shown at 50% probability (C = black, N = blue, P = orange, Ce = purple, K = green, O = red). Hydrogen atoms and disorder are omitted for clarity.

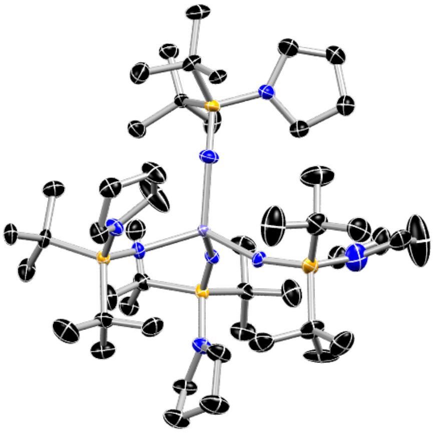

Figure S34. Molecular structure of **2-Ce(NPC²)**. Thermal ellipsoids are shown at 50% probability (C = black, N = blue, P = orange, Ce = purple). Hydrogen atoms and disorder are omitted for clarity.

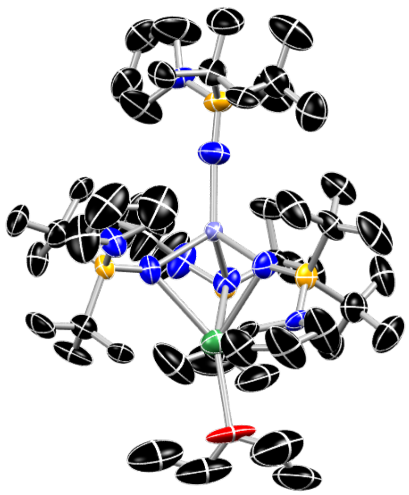

Figure S35. Molecular structure of **1-KPr(NPC[2])**. Thermal ellipsoids are shown at 50% probability (C = black, N = blue, P = orange, Pr = purple, K = green, O = red). Hydrogen atoms and disorder are omitted for clarity.

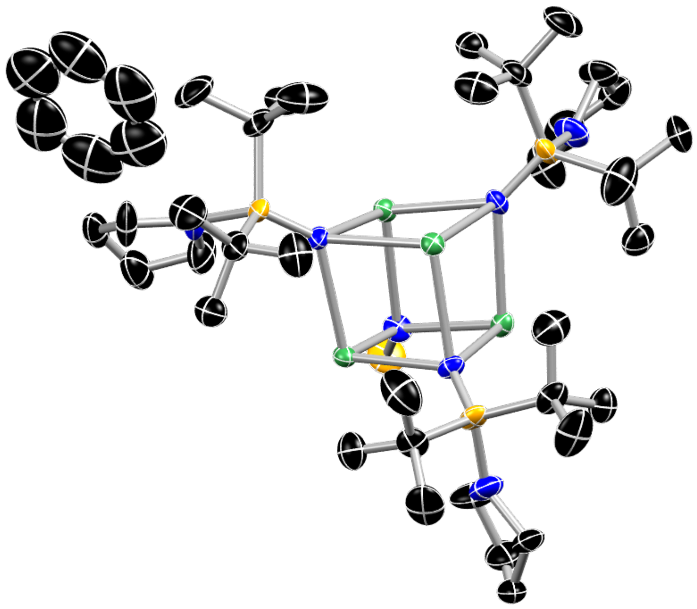

Figure S36. Molecular structure of **KNPC[2]**. Thermal ellipsoids are shown at 50% probability (C = black, N = blue, P = orange, K = green). Hydrogen atoms and toluene disorder are omitted for clarity.

Further Computational Details

Table S3. Optimized bond lengths (Å) and angles (°) for **Ln(NPC¹)**. Experimental values, when available, are shown in brackets.[5]

	1-Ce(NPC¹)	2-Ce(NPC¹)	1-Pr(NPC¹)	2-Pr(NPC¹)	3-Pr(NPC¹)
Ln–N	2.341 [2.315(5)]	2.198 [2.160(6)]	2.320	2.200	2.208
Ln---P	3.873	3.756	3.857	3.761	3.792
N–P	1.541 [1.540(18)]	1.562 [1.539(1)]	1.540	1.563	1.588
Ln–N–P	173.2 [147.6(3)]	175.4 [157(7)]	176.2	176.8	175.1
τ_4 $\theta_{N–Ln–N}$	0.98	0.99	0.96	0.98	0.95
$\sum_{\Delta 109.5}$ $\theta_{N–Ln–N}$	6.6	4.1	10.1	6.3	19.2
$\sum_{\Delta 109.5}$ $\theta_{P–Ln–P}$	9.2	5.0	10.8	8.2	14.7

Table S4. Optimized bond lengths (Å) and angles (°) for **Ln(NPC²)**. Experimental values, when available, are shown in brackets.

	1-Ce(NPC²)	2-Ce(NPC²)	1-Pr(NPC²)	2-Pr(NPC²)	3-Pr(NPC²)
Ln–N	2.340	2.203 [2.16(4)]	2.324	2.211	2.233
Ln---P	3.882	3.767 [3.67(5)]	3.860	3.773	3.820
N–P	1.546	1.569 [1.57(4)]	1.547	1.571	1.599
Ln–N–P	175.5	174.3 [158(7)]	172.2	172.8	175.3
τ_4 $\theta_{N–Ln–N}$	0.99	0.99 [0.97]	0.98	0.99	0.97
$\sum_{\Delta 109.5}$ $\theta_{N–Ln–N}$	2.0	3.2 [10(2)]	6.2	3.8	8.9
$\sum_{\Delta 109.5}$ $\theta_{P–Ln–P}$	1.1 [24(5)]	2.7 [19(4)]	3.8 [12(3)]	8.5	5.6

Table S5. Optimized bond lengths (Å) and angles (°) for **Ln(NPC³)**. Experimental values, when available, are shown in brackets.[2]

	1-Ce(NPC³)	2-Ce(NPC³)	1-Pr(NPC³)	2-Pr(NPC³)	3-Pr(NPC³)
Ln–N	2.339	2.209 [2.176(4)]	2.327	2.212 [2.179(3)]	2.219 [2.25(5)]
Ln---P	3.895	3.787 [3.70(1)]	3.882	3.790 [3.69(1)]	3.826 [3.80(3)]
N–P	1.556	1.579 [1.562(3)]	1.555	1.580 [1.561(1)]	1.607 [1.57(1)]
Ln–N–P	179.9	176.7 [163(1)]	179.3	176.6 [162(1)]	177.2 [166(3)]
τ_4 $\theta_{N\text{–}Ln\text{–}N}$	1.00	1.00 [0.98]	0.99	1.00 [0.97]	0.98 [1.00]
$\sum_{\Delta 109.5}$ $\theta_{N\text{–}Ln\text{–}N}$	0.2	1.2 [6.9(5)]	3.9	1.3 [9.94(12)]	9.4 [20.7(6)]
$\sum_{\Delta 109.5}$ $\theta_{P\text{–}Ln\text{–}P}$	0.2	0.9 [6(2)]	2.2	2.1 [9(2)]	4.4 [8(2)]

Table S6. Optimized bond lengths (Å) and angles (°) for **KLn(NPC¹)**. Measurements are shown with N_{capped} values before the slash and $N_{terminal}$ values after here and elsewhere.

	1-KCe(NPC¹)	2-KCe(NPC¹)	1-KPr(NPC¹)	2-KPr(NPC¹)	3-KPr(NPC¹)
Ln–M	3.196	3.225	3.191	3.221	3.285
Ln–N	2.356/2.309	2.220/2.169	2.339/2.287	2.223/2.168	2.210/2.361
Ln---P	1.553/1.553	1.575/1.581	3.778/3.834	3.723/3.744	1.595/1.606
N–P	3.802/3.858	3.707/3.744	1.553/1.553	1.577/1.582	3.711/3.962
Ln–N–P	153.1/174.6	155.6/173.4	151.7/173.6	157.2/173.1	154.6/174.2
τ_4 $\theta_{N\text{–}Ln\text{–}N}$	0.81	0.84	0.81	0.84	0.81
$\sum_{\Delta 109.5}$ $\theta_{N\text{–}Ln\text{–}N}$	82.8	69.2	83.1	65.7	71.7
$\sum_{\Delta 109.5}$ $\theta_{P\text{–}Ln\text{–}P}$	12.8	5.5	14.4	6.4	10.6

Table S7. Optimized bond lengths (Å) and angles (°) for **KLn(NPC²)**. Experimental values, when available, are shown in brackets.

	1-KCe(NPC²)	2-KCe(NPC²)	1-KPr(NPC²)	2-KPr(NPC²)	3-KPr(NPC²)
Ln–M	3.182	3.216	3.190	3.249	3.303
Ln–N	2.366/2.301 [2.34(5)/2.32(4)]	2.234/2.173	2.346/2.290 [2.31(1)/2.29(6)]	2.227/2.198	2.212/2.469
Ln---P	3.829/3.862 [3.73(10)]	1.586/1.593	3.824/3.844 [3.75(5)]	3.755/3.789	1.607/1.635
N–P	1.561/1.562 [1.55(1)]	3.753/3.764	1.561/1.562 [1.53(2)]	1.585/1.594	3.744/4.103
Ln–N–P	154.6/176.5 [152(18)]	158.9/176.4	156.5/172.2 [158(1)]	161.2/175.4	157.3/177.1
τ_4 $\theta_{N\text{–}Ln\text{–}N}$	0.82 [0.92]	0.85	0.81 [0.83]	0.84	0.81
$\sum_{\Delta 109.5}$ $\theta_{N\text{–}Ln\text{–}N}$	81.0 [84(15)]	65.6	80.6 [77(14)]	61.9	69.7
$\sum_{\Delta 109.5}$ $\theta_{P\text{–}Ln\text{–}P}$	15.1 [24(5)]	10.0	18.1 [12(3)]	12.4	16.9

Table S8. Optimized bond lengths (Å) and angles (°) for **KLn(NPC³)**.

	1-KCe(NPC³)	2-KCe(NPC³)	1-KPr(NPC³)	2-KPr(NPC³)	3-KPr(NPC³)
Ln–M	3.180	3.200	3.176	3.194	3.268
Ln–N	2.376/2.311	2.246/2.185	2.361/2.289	2.257/2.191	2.238/2.443
Ln---P	1.572/1.573	1.598/1.605	3.851/3.862	3.798/3.794	1.618/1.649
N–P	3.861/3.884	3.785/3.789	1.572/1.573	1.599/1.603	3.794/4.091
Ln–N–P	155.5/180.0	159.8/180.0	155.6/179.5	159.8/179.3	159.3/178.4
τ_4 $\theta_{N\text{–}Ln\text{–}N}$	0.83	0.86	0.82	0.86	0.85
$\sum_{\Delta 109.5}$ $\theta_{N\text{–}Ln\text{–}N}$	79.4	63.5	78.7	63.9	66.1
$\sum_{\Delta 109.5}$ $\theta_{P\text{–}Ln\text{–}P}$	20.5	13.3	20.3	13.4	11.8

Table S9. Optimized bond lengths (Å) and angles (°) for **CsLn(NPC¹)**.

	1-CsCe(NPC¹)	2-CsCe(NPC¹)	1-CsPr(NPC¹)	2-CsPr(NPC¹)	3-CsPr(NPC¹)
Ln–M	3.571	3.666	3.569	3.650	3.797
Ln–N	2.361/2.313	2.225/2.173	2.345/2.289	2.229/2.175	2.205/2.392
Ln---P	1.552/1.553	1.574/1.580	3.799/3.837	3.731/3.749	1.592/1.608
N–P	3.814/3.861	3.723/3.748	1.551/1.552	1.575/1.579	3.711/3.989
Ln–N–P	154.0/174.0	157.2/174.6	154.2/174.1	157.5/174.2	155.5/171.3
τ_4 $\theta_{N\text{–}Ln\text{–}N}$	0.85	0.90	0.86	0.89	0.86
$\sum_{\Delta 109.5}$ $\theta_{N\text{–}Ln\text{–}N}$	60.0	44.4	59.4	43.3	43.3
$\sum_{\Delta 109.5}$ $\theta_{P\text{–}Ln\text{–}P}$	8.6	14.5	10.6	14.3	29.5

Table S10. Optimized bond lengths (Å) and angles (°) for **CsLn(NPC²)**. Measurements are shown with N_{capped} values before the slash and $N_{terminal}$ values after.

	1-CsCe(NPC²)	2-CsCe(NPC²)	1-CsPr(NPC²)	2-CsPr(NPC²)	3-CsPr(NPC²)
Ln–M	3.572	3.729	3.582	3.726	3.849
Ln–N	2.369/2.314	2.236/2.185	2.352/2.302	2.238/2.219	2.197/2.508
Ln---P	1.559/1.563	1.583/1.591	3.831/3.860	3.761/3.811	1.604/1.635
N–P	3.834/3.874	3.751/3.772	1.560/1.562	1.584/1.593	3.739/4.143
Ln–N–P	155.2/176.0	160.1/174.7	156.7/174.9	160.7/177.1	159.7/178.0
τ_4 $\theta_{N\text{–}Ln\text{–}N}$	0.86	0.90	0.87	0.88	0.89
$\sum_{\Delta 109.5}$ $\theta_{N\text{–}Ln\text{–}N}$	60.2	42.3	57.7	39.8	34.9
$\sum_{\Delta 109.5}$ $\theta_{P\text{–}Ln\text{–}P}$	15.4	15.9	6.9	16.1	23.8

Table S11. Optimized bond lengths (Å) and angles (°) for **CsLn(NPC^3)**. Experimental values, when available, are shown in brackets.[2]

	**1-CsCe(NPC^3)**	**2-CsCe(NPC^3)**	**1-CsPr(NPC^3)**	**2-CsPr(NPC^3)**	**3-CsPr(NPC^3)**
Ln–M	3.567	3.658	3.564	3.650	3.810
Ln–N	2.382/2.320<br>[2.34(1)]	2.254/2.198	2.367/2.300<br>[2.33(1)]	2.264/2.209	2.235/2.489
Ln---P	3.883/3.894<br>[3.82(4)]	1.597/1.606	3.869/3.873<br>[3.81(3)]	3.817/3.814	1.617/1.651
N–P	1.570/1.574<br>[1.552(2)]	3.806/3.804	1.570/1.574<br>[1.552(1)]	1.598/1.605	3.808/4.140
Ln–N–P	158.0/179.9<br>[160(12)]	162.0/180.0	158.2/179.4<br>[160(1)]	162.2/179.5	162.6/179.0
$\tau_4\ \theta_{N\text{–}Ln\text{–}N}$	0.87<br>[0.85]	0.91	0.87<br>[0.85]	0.91	0.90
$\sum_{\Delta 109.5}\ \theta_{N\text{–}Ln\text{–}N}$	57.0<br>[61.92(15)]	42.5	56.5<br>[60.94(12)]	41.4	35.3
$\sum_{\Delta 109.5}\ \theta_{P\text{–}Ln\text{–}P}$	4.0<br>[5(1)]	1.6	3.9<br>[5(1)]	2.5	8.6

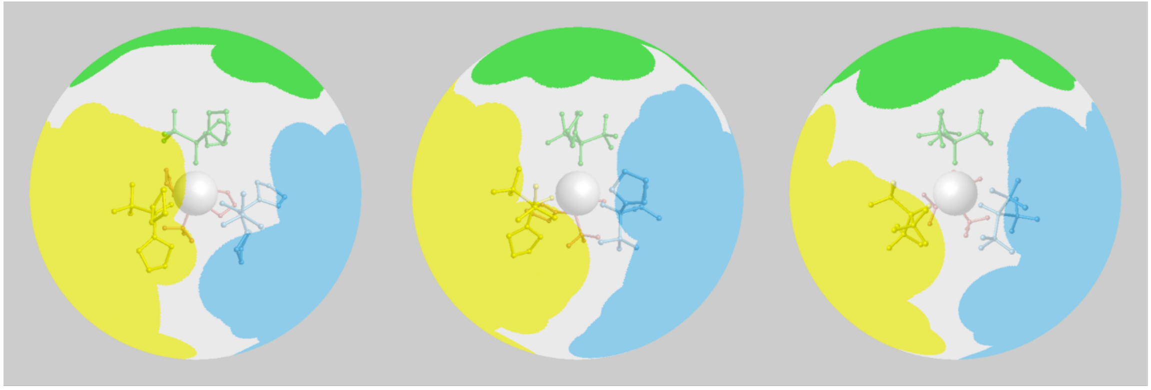

1-Pr(NPC^{1}) **1-Pr(NPC^{2})** **1-Pr(NPC^{3})**

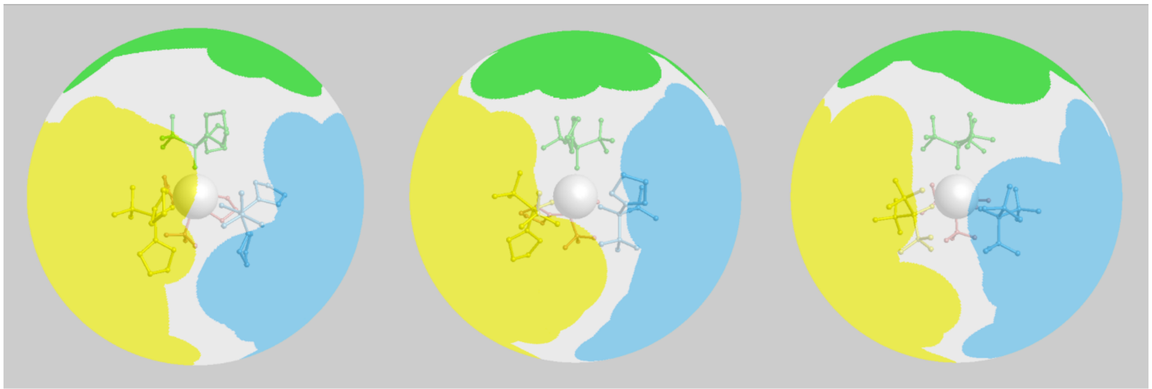

2-Pr(NPC^{1}) **2-Pr(NPC^{2})** **2-Pr(NPC^{3})**

Figure S37. Visualization of the G-parameter for **1-Pr(NPC^{x})** and **2-Pr(NPC^{x})** complexes.

Table S12. G_M(complex, %) computed for **Ln(NPC^{x})** complexes.

Ligand	1-Ce(NPC^{x})	1-Pr(NPC^{x})	2-Ce(NPC^{x})	2-Pr(NPC^{x})	3-Pr(NPC^{x})
NPC^{1}	73.9	73.4	78.0	77.4	77.5
NPC^{2}	73.8	75.2	79.8	79.3	79.0
NPC^{3}	75.4	75.8	80.1	80.0	80.5

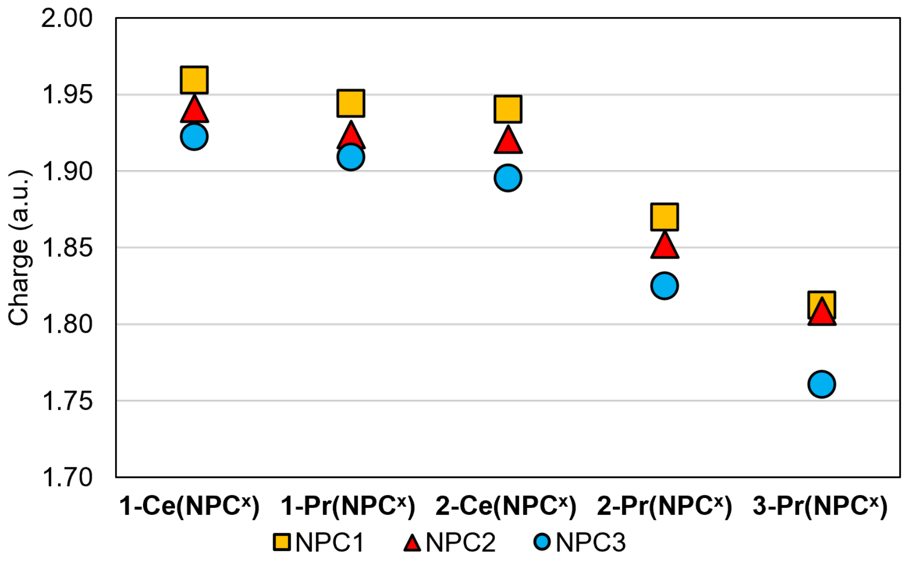

Figure S38. NPA charge (Q_{NPA}) calculated for the Ln atom in Ln(NPC^x) complexes. See Table S12 for detailed values.

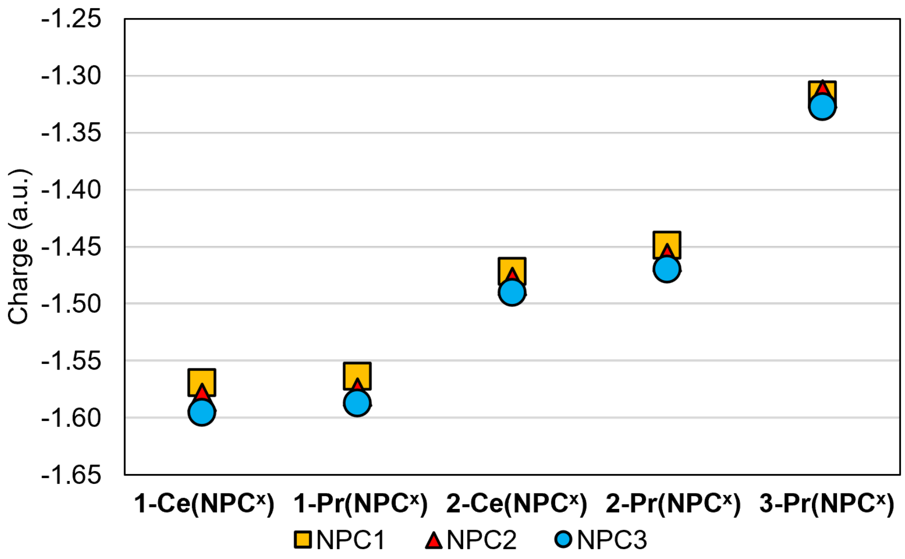

Figure S39. NPA charge (Q_{NPA}) calculated for the N_{im} atom in Ln(NPC^x) complexes. See Table S13 for detailed values.

Table S13. NPA charge (Q_{NPA}) calculated for the Ln atom in Ln(NPC^x) complexes (a.u.).

Ligand	1-Ce(NPC^x)	1-Pr(NPC^x)	2-Ce(NPC^x)	2-Pr(NPC^x)	3-Pr(NPC^x)
NPC^1	1.96	1.94	1.94	1.87	1.81
NPC^2	1.94	1.92	1.92	1.85	1.81
NPC^3	1.92	1.91	1.90	1.83	1.76

Table S14. NPA charge (Q_{NPA}) calculated for the imine N atom (N_{im}) in Ln(NPC^x) complexes (a.u.).

Ligand	1-Ce(NPC^x)	1-Pr(NPC^x)	2-Ce(NPC^x)	2-Pr(NPC^x)	3-Pr(NPC^x)
NPC^1	-1.58	-1.56	-1.47	-1.45	-1.32
NPC^2	-1.58	-1.58	-1.48	-1.46	-1.32
NPC^3	-1.60	-1.59	-1.49	-1.47	-1.33

Table S15. Electron density (ρ) at the N_{im} nuclear critical point (NCP) from QTAIM (|e|/Bohr^3).

Ligand	1-Ce(NPC^x)	1-Pr(NPC^x)	2-Ce(NPC^x)	2-Pr(NPC^x)	3-Pr(NPC^x)
NPC^1	194.741	194.734	194.741	194.758	194.855
NPC^2	194.735	194.732	194.741	194.763	194.863
NPC^3	194.755	194.751	194.762	194.782	194.881

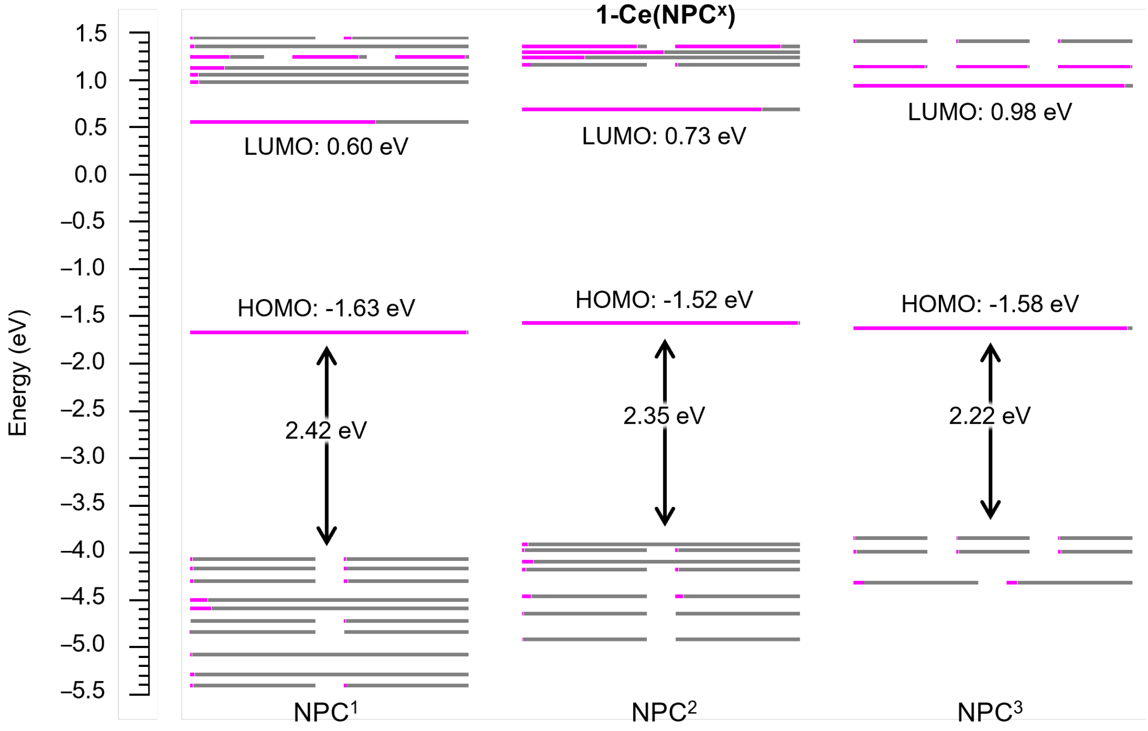

Figure S40. MO energy diagram for **1-Ce(NPC^x)** complexes. The pink portion of the energy bar represents the contribution of the lanthanide orbitals, and the gray portion represents the contribution of the ligand orbitals, here and elsewhere in the SI. Degeneracy of the orbitals is set to 0.05 eV, with degenerate orbitals depicted using smaller sticks, here and elsewhere in the SI.

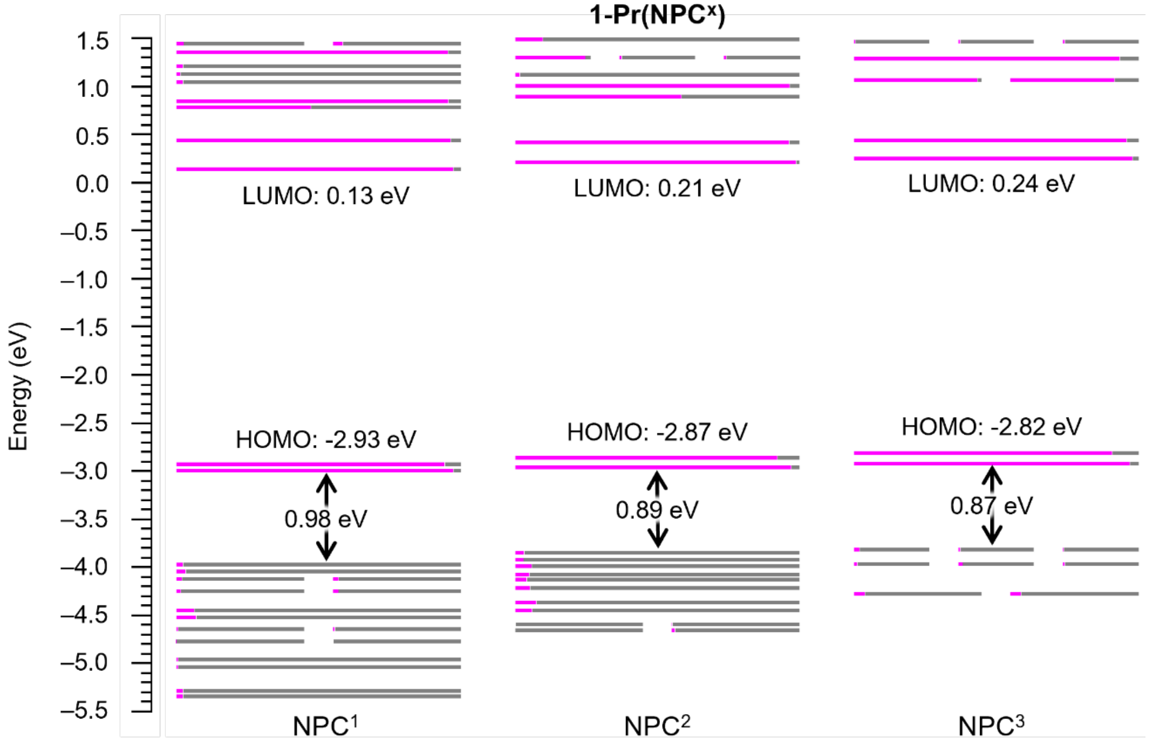

Figure S41. MO energy diagram for **1-Pr(NPC^x)** complexes.

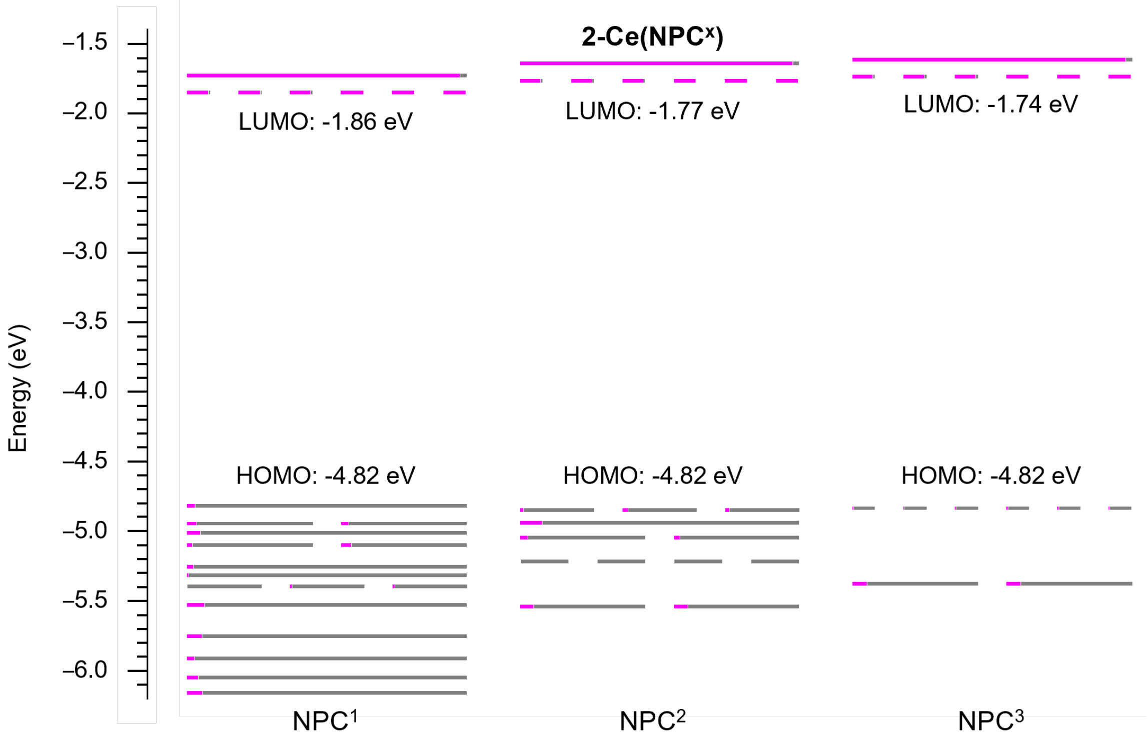

Figure S42. MO energy diagram for **2-Ce(NPC^x)** complexes.

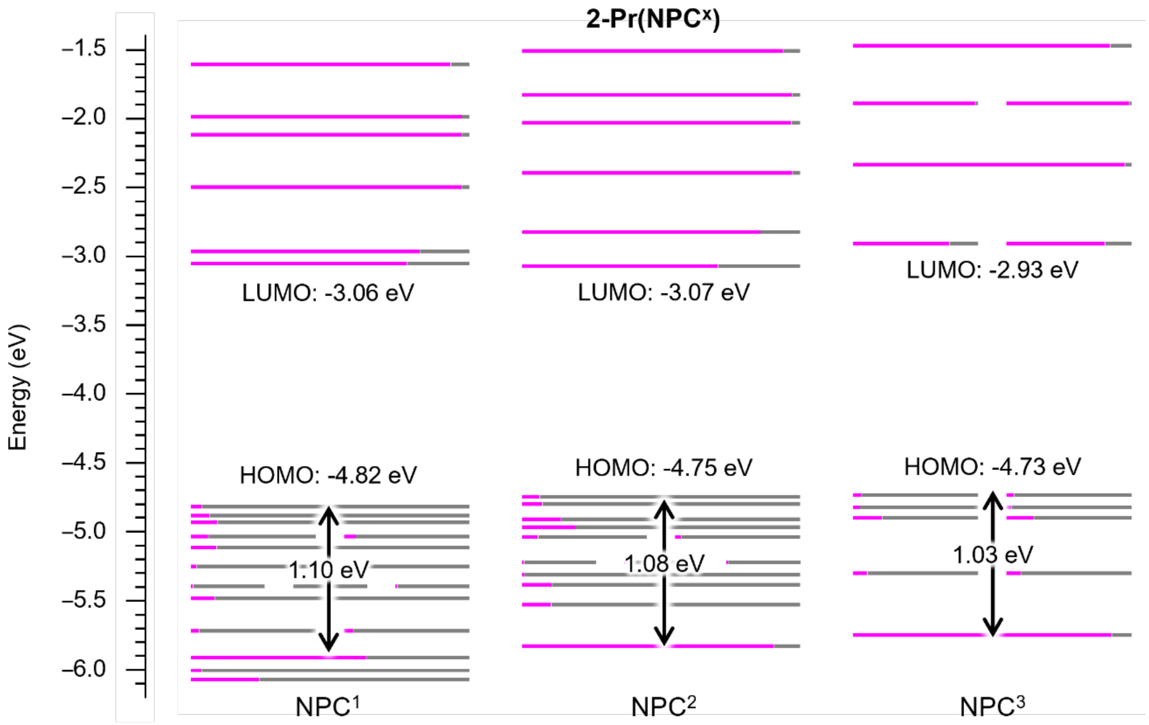

Figure S43. MO energy diagram for **2-Pr(NPCˣ)** complexes.

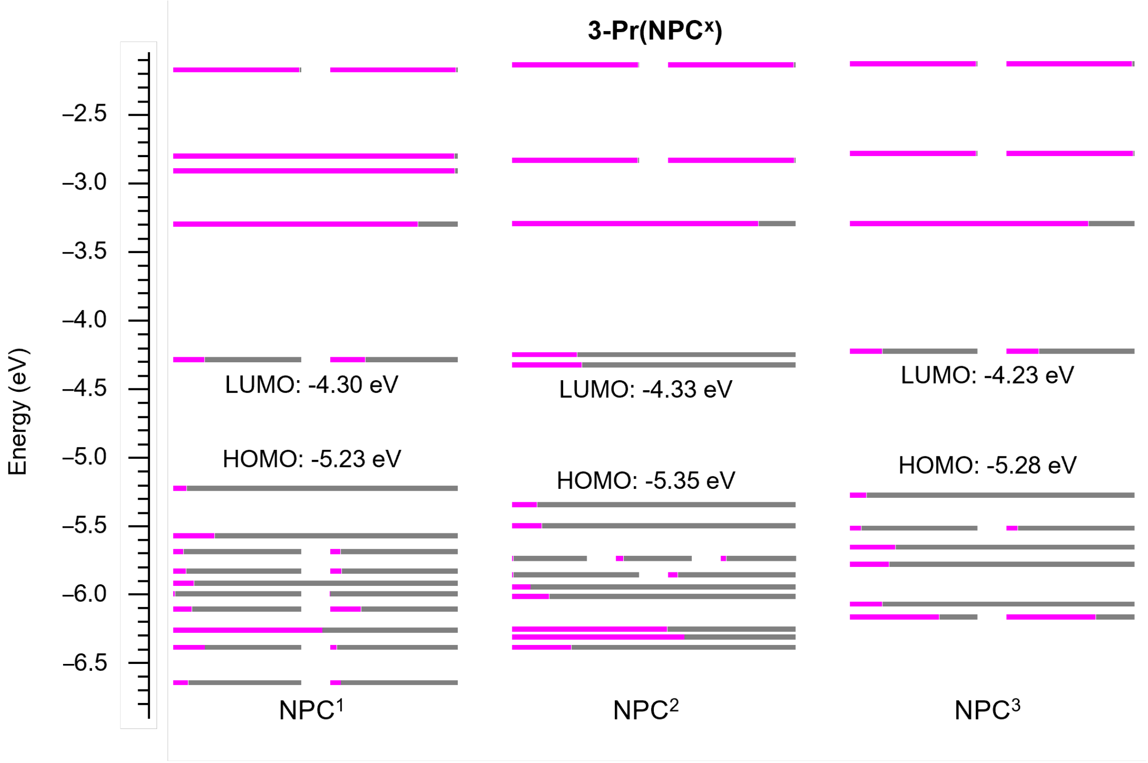

Figure S44. MO energy diagram for **3-Pr(NPCˣ)** complexes.

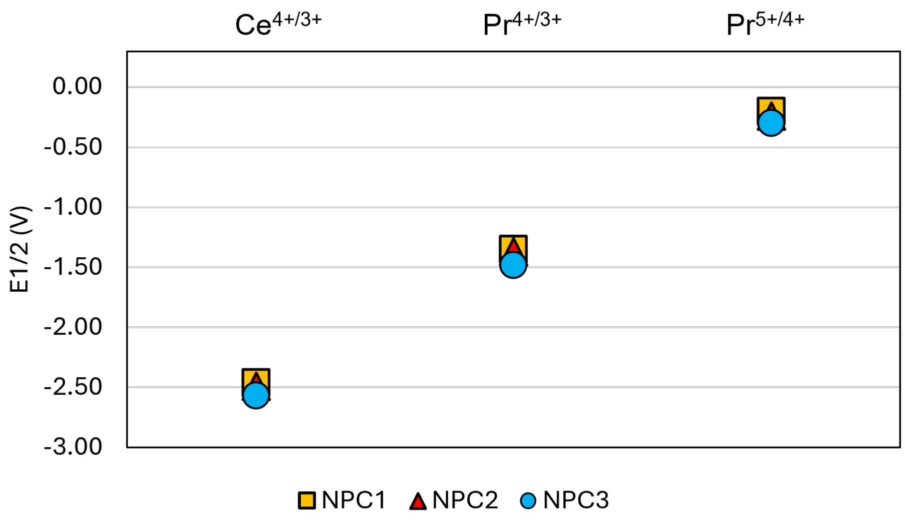

Figure S45. Calculated redox potentials (V) for **1-Ln(NPC^x)**. See Table S15 for detailed values.

Table S16. Calculated redox potentials (V) for **Ln(NPC^x)**. Experimental E_{pa}/E_{pc} values are shown below in parentheses when available. The trivalent experimental NPC^2 complexes include a [K$^+$] cation and the trivalent experimental NPC^3 complexes include a [Cs$^+$] cation.

Ligand	$Ce^{4+/3+}$	$Pr^{4+/3+}$	$Pr^{5+/4+}$
NPC^1	-2.46<br>(-2.28/-2.91)	-1.35	-0.20
NPC^2	-2.49<br>(-1.69/-3.14)	-1.37<br>(-0.60/-1.47)	-0.24<br>(-0.16/-0.33)
NPC^3	-2.57<br>(-2.26/-3.01)	-1.48<br>(-1.26/-1.45)	-0.30<br>(-0.24/-0.35)

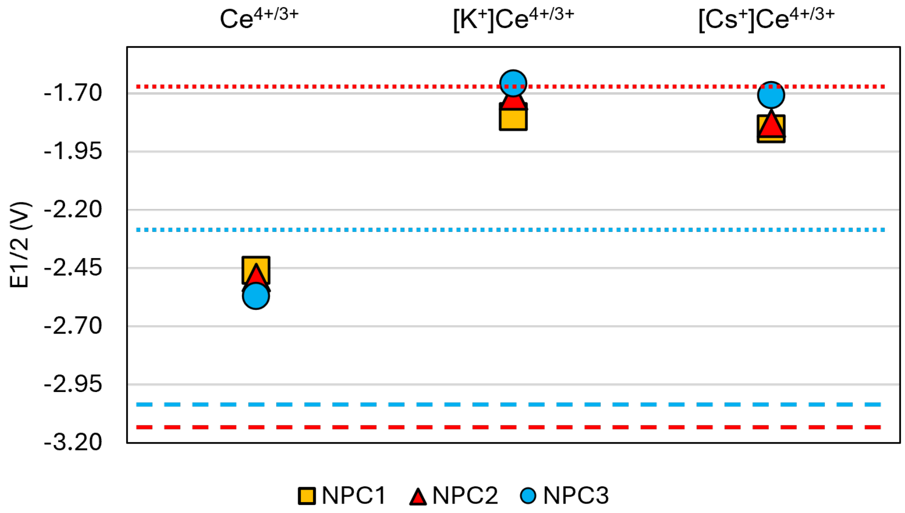

Figure S46. Graphical representation of the calculated redox potentials (V) for **1-M$^+$Ce(NPC^x)**. Experimental E_{pa} values are shown with dotted lines while E_{pc} values are shown with dashed lines with the color corresponding to the ligand with the same color marker. The trivalent experimental NPC^2 complexes include a [K$^+$] cation and the trivalent experimental NPC^3 complexes include a [Cs$^+$] cation.

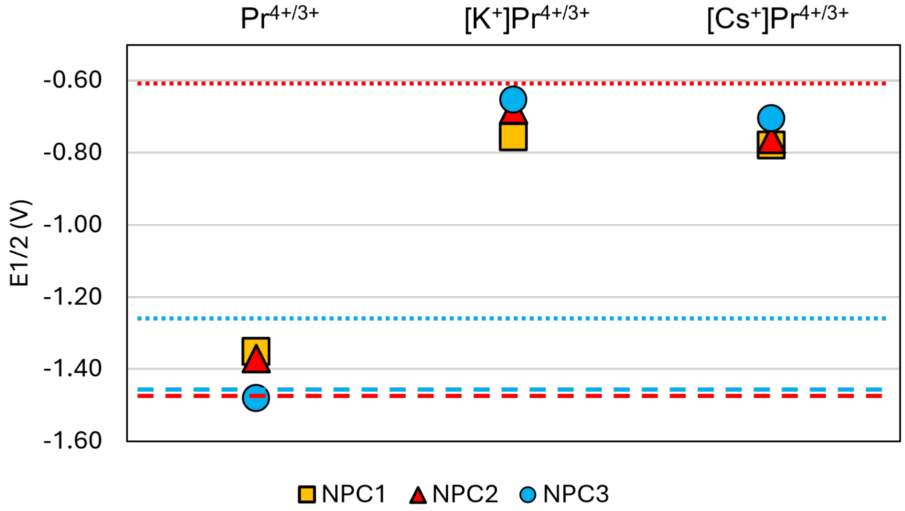

Figure S47. Graphical representation of the calculated redox potentials (V) for **1-M$^+$Pr(NPC^x)**. Experimental E_{pa} values are shown with dotted lines while E_{pc} values are shown with dashed lines with the color corresponding to the ligand with the same color marker. The trivalent experimental NPC^2 complexes include a [K$^+$] cation and the trivalent experimental NPC^3 complexes include a [Cs$^+$] cation.

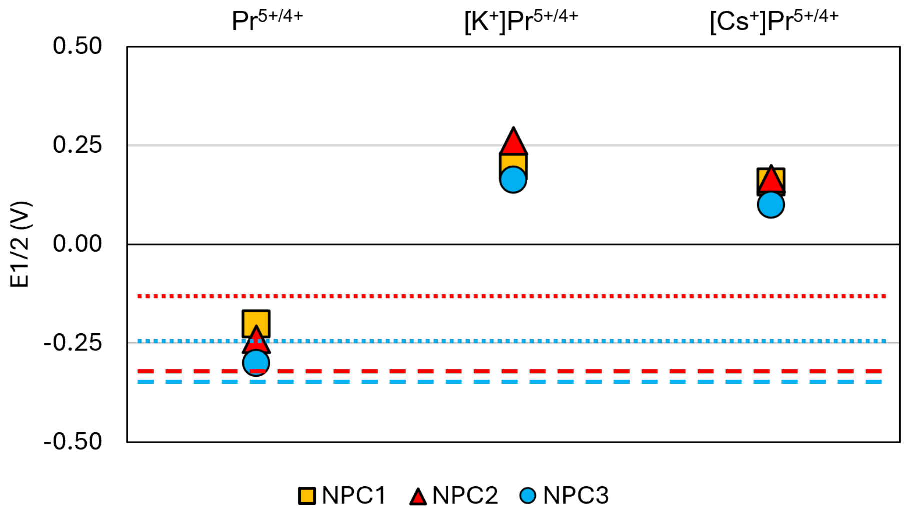

Figure S48. Graphical representation of the calculated redox potentials (V) for **2-M$^+$Pr(NPC^x)**. Experimental E_{pa} values are shown with dotted lines while E_{pc} values are shown with dashed lines with the color corresponding to the ligand with the same color marker.

Table S17. Calculated redox potentials (V) for **M$^+$Ln(NPC^x)** (M = K, Cs). The calculation assumes the cation is retained throughout the whole process and the molecule is allowed to adiabatically relax. Calculations were calibrated by using an isodesmic reaction with the ferrocene couple, *i.e.*, **1-M$^+$Ln(NPC^x)** + Fc^+ → **2-M$^+$Ln(NPC^x)** + Fc^0. Experimental E_{pa}/E_{pc} values are shown below when available.

Ligands	$KCe^{4+/3+}$	$CsCe^{4+/3+}$	$KPr^{4+/3+}$	$CsPr^{4+/3+}$	$KPr^{5+/4+}$	$CsPr^{5+/4+}$
NPC^1	-1.80	-1.85	-0.76	-0.78	+0.20	+0.16
	(-2.28/-2.91)					
NPC^2	-1.71	-1.83	-0.68	-0.76	+0.26	+0.17
	(-1.69/-3.14)		(-0.60/-1.47)		(-0.16/-0.33)	
NPC^3	-1.66	-1.71	-0.65	-0.70	+0.16	+0.10
	(-2.26/-3.01)		(-1.26/-1.45)		(-0.24/-0.35)	

Table S18. Calculated vertical/adiabatic detachment energy (VDE/ADE) and vertical/adiabatic electron affinity (VEA/AEA) for **$M^{+}Ln(NPC^{x})$** (M = None, K, Cs). All are in units of V. Vertical energies assume no rearrangement upon oxidation/reduction while adiabatic energies assume total relaxation upon oxidation/reduction.

Cation	Ligand	Redox Couple	VDE	VEA	ADE	AEA	|VDE - ADE|	|AEA - VEA|
None	NPC^{1}	Ce^{4+}/Ce^{3+}	-2.01	-3.39	-2.69	-2.69	0.68	0.70
None	NPC^{2}	Ce^{4+}/Ce^{3+}	-2.14	-3.48	-2.71	-2.79	0.57	0.69
None	NPC^{3}	Ce^{4+}/Ce^{3+}	-2.32	-3.49	-2.81	-2.79	0.49	0.70
K	NPC^{1}	Ce^{4+}/Ce^{3+}	-1.44	-2.91	-1.99	-1.99	0.55	0.92
K	NPC^{2}	Ce^{4+}/Ce^{3+}	-1.43	-2.70	-1.92	-1.92	0.49	0.77
K	NPC^{3}	Ce^{4+}/Ce^{3+}	-1.44	-2.73	-1.89	-1.89	0.45	0.84
Cs	NPC^{1}	Ce^{4+}/Ce^{3+}	-1.47	-2.80	-2.06	-2.06	0.59	0.74
Cs	NPC^{2}	Ce^{4+}/Ce^{3+}	-1.47	-2.75	-2.05	-2.05	0.59	0.69
Cs	NPC^{3}	Ce^{4+}/Ce^{3+}	-1.48	-2.76	-1.96	-1.96	0.48	0.80
None	NPC^{1}	Pr^{4+}/Pr^{3+}	-1.07	-2.11	-1.58	-1.58	0.51	0.53
None	NPC^{2}	Pr^{4+}/Pr^{3+}	-1.22	-2.12	-1.62	-1.66	0.40	0.46
None	NPC^{3}	Pr^{4+}/Pr^{3+}	-1.28	-2.14	-1.65	-1.70	0.37	0.45
K	NPC^{1}	Pr^{4+}/Pr^{3+}	-0.42	-1.49	-0.88	-0.88	0.46	0.60
K	NPC^{2}	Pr^{4+}/Pr^{3+}	-0.47	-1.49	-0.87	-0.87	0.39	0.63
K	NPC^{3}	Pr^{4+}/Pr^{3+}	-0.62	-1.29	-0.84	-0.84	0.22	0.45
Cs	NPC^{1}	Pr^{4+}/Pr^{3+}	-0.46	-1.53	-0.96	-0.96	0.50	0.56
Cs	NPC^{2}	Pr^{4+}/Pr^{3+}	-0.57	-1.51	-0.97	-0.97	0.39	0.54
Cs	NPC^{3}	Pr^{4+}/Pr^{3+}	-0.62	-1.33	-0.91	-0.91	0.29	0.41
None	NPC^{1}	Pr^{5+}/Pr^{4+}	0.01	-0.37	-0.26	-0.26	0.26	0.12
None	NPC^{2}	Pr^{5+}/Pr^{4+}	-0.10	-0.40	-0.30	-0.30	0.20	0.10
None	NPC^{3}	Pr^{5+}/Pr^{4+}	-0.19	-0.58	-0.35	-0.35	0.16	0.23

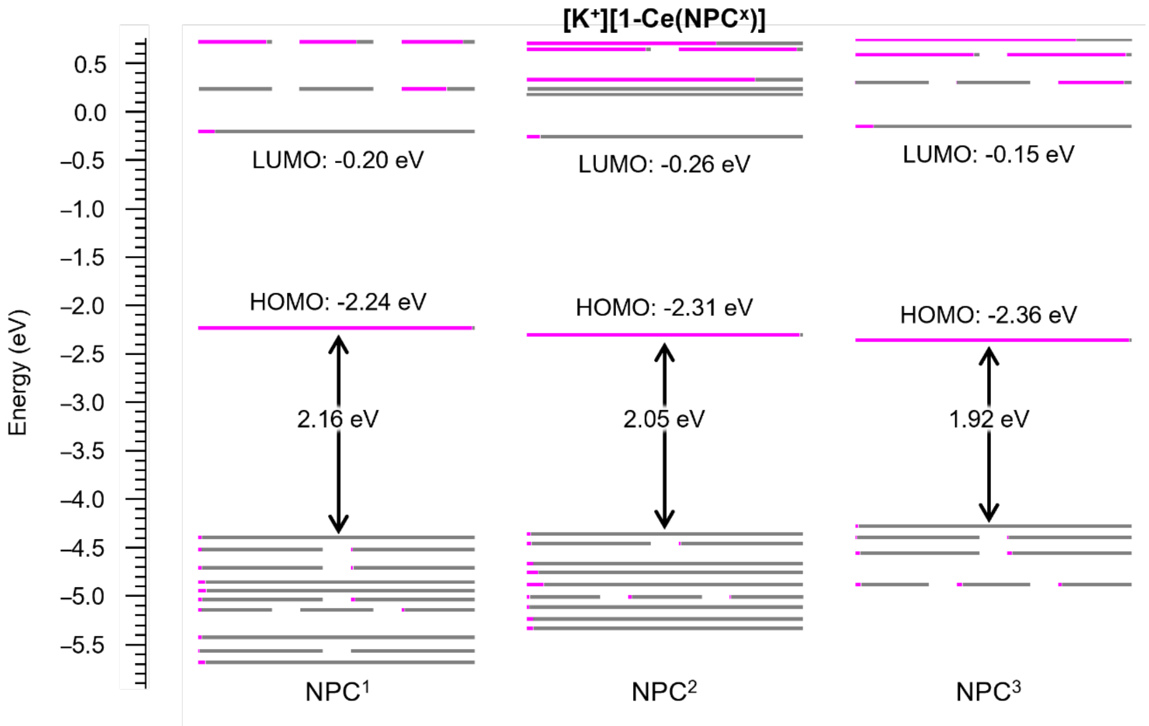

Figure S49. MO energy diagram for **1-KCe(NPC^x)** complexes.

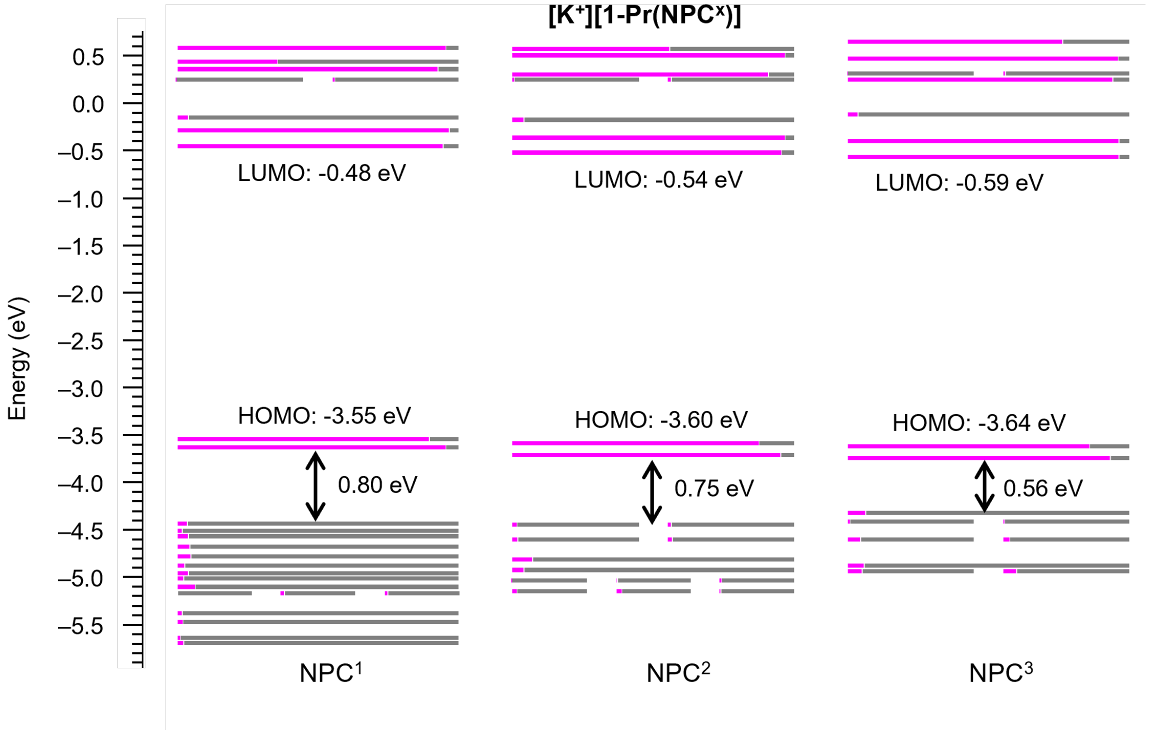

Figure S50. MO energy diagram for **1-KPr(NPC^x)** complexes.

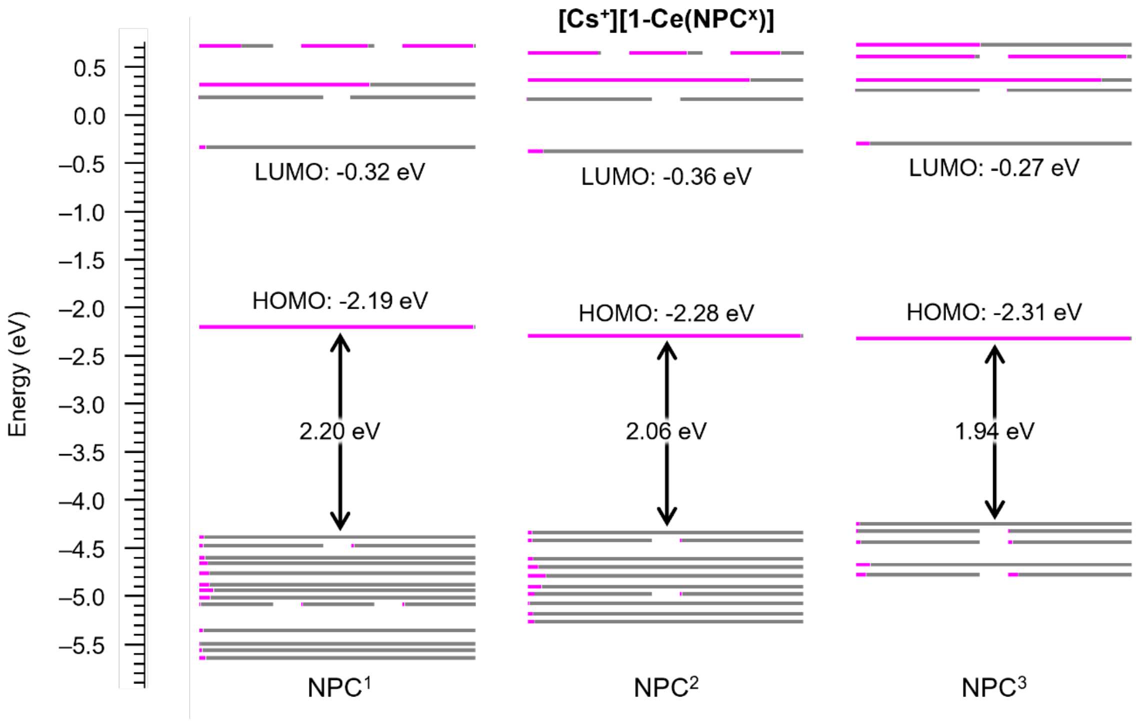

Figure S51. MO energy diagram for **1-CsCe(NPC^x)** complexes.

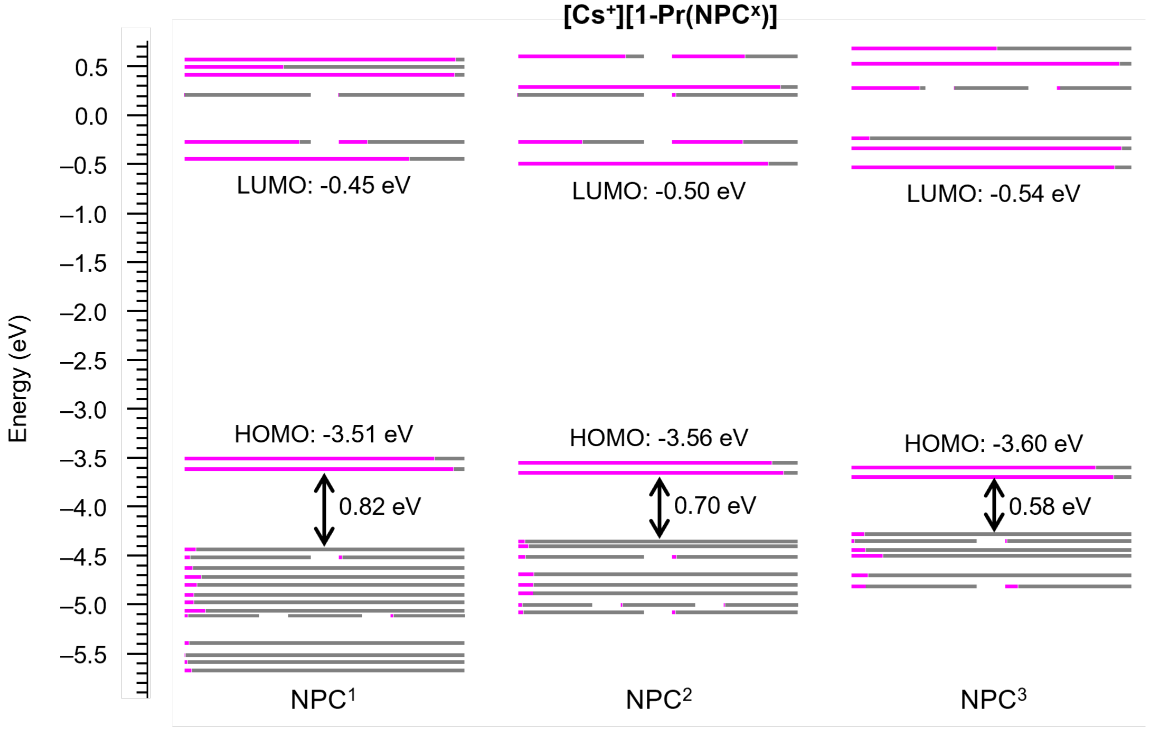

Figure S52. MO energy diagram for **1-CsPr(NPC^x)** complexes.

Table S19. Energy (kcal/mol) required to eject the [K^+] and [Cs^+] ions intercalated with the **Ln(NPC^x)** complexes. The following chemical equation was used to measure the adiabatic dissociation energy [Ln^{q+}(NPC^x)][M^+] → [Ln^{q+}(NPC^x)] + [M^+].

[M^+][Ligand]	**[1-Ce(NPC^x)]**	**[1-Pr(NPC^x)]**	**[2-Ce(NPC^x)]**	**[2-Pr(NPC^x)]**	**[3-Pr(NPC^x)]**
[K^+][NPC^1]	15.62	18.17	0.42	3.28	-5.92
[K^+][NPC^2]	16.61	16.88	-1.45	1.06	-10.43
[K^+][NPC^3]	14.96	13.11	-6.19	-5.94	-16.54
[Cs^+][NPC^1]	5.24	7.05	-8.77	-6.14	-14.40
[Cs^+][NPC^2]	5.63	4.22	-9.74	-9.72	-19.01
[Cs^+][NPC^3]	2.24	0.20	-17.74	-17.68	-26.83

References

(1) Wedal, J. C.; Barlow, J. M.; Ziller, J. W.; Yang, J. Y.; Evans, W. J. Electrochemical studies of tris(cyclopentadienyl)thorium and uranium complexes in the +2, +3, and +4 oxidation states. *Chem. Sci.* **2021**, *12* (24), 8501-8511.
(2) Boggiano, A. C.; Studvick, C. M.; Roy Chowdhury, S.; Niklas, J. E.; Tateyama, H.; Wu, H.; Leisen, J. E.; Kleemiss, F.; Vlaisavljevich, B.; Popov, I. A.; La Pierre, H. S. Praseodymium in the Formal +5 Oxidation State. *Nat. Chem.* **2025**, *17*, 1005–1010.
(3) Rice, N. T.; Popov, I. A.; Russo, D. R.; Gompa, T. P.; Ramanathan, A.; Bacsa, J.; Batista, E. R.; Yang, P.; La Pierre, H. S. Comparison of tetravalent cerium and terbium ions in a conserved, homoleptic imidophosphorane ligand field. *Chem. Sci.* **2020**, *11* (24), 6149-6159.
(4) Rice, N. T.; Popov, I. A.; Carlson, R. K.; Greer, S. M.; Boggiano, A. C.; Stein, B. W.; Bacsa, J.; Batista, E. R.; Yang, P.; La Pierre, H. S. Spectroscopic and electrochemical characterization of a Pr^{4+} imidophosphorane complex and the redox chemistry of Nd^{3+} and Dy^{3+} complexes. *Dalton Trans.* **2022**, *51* (17), 6696-6706.
(5) Otte, K. S.; Niklas, J. E.; Studvick, C. M.; Boggiano, A. C.; Bacsa, J.; Popov, I. A.; La Pierre, H. S. Divergent Stabilities of Tetravalent Cerium, Uranium, and Neptunium Imidophosphorane Complexes. *Angew. Chem. Int. Ed.* **2023**, *62* (34), e202306580.
